# Improving the efficiency and effectiveness of an industrial SARS-CoV-2 diagnostic facility

**DOI:** 10.1038/s41598-022-06873-6

**Published:** 2022-02-24

**Authors:** Julie A. Douthwaite, Christopher A. Brown, John R. Ferdinand, Rahul Sharma, Jane Elliott, Molly A. Taylor, Nancy T. Malintan, Hannah Duvoisin, Thomas Hill, Oona Delpuech, Alexandra L. Orton, Haidee Pitt, Fred Kuenzi, Simon Fish, David J. Nicholls, Anna Cuthbert, Ian Richards, Giles Ratcliffe, Abhishek Upadhyay, Abigail Marklew, Craig Hewitt, Douglas Ross-Thriepland, Christopher Brankin, Matthieu Chodorge, Gareth Browne, Palwinder K. Mander, Ruud M. DeWildt, Shane Weaver, Penny A. Smee, Joost van Kempen, Jon G. Bartlett, Paula M. Allen, Emma L. Koppe, Charlotte A. Ashby, Julian D. Phipps, Nalini Mehta, David J. Brierley, David G. Tew, Melanie V. Leveridge, Stuart M. Baddeley, Ian G. Goodfellow, Clive Green, Chris Abell, Andy Neely, Ian Waddell, Steve Rees, Patrick H. Maxwell, Menelas N. Pangalos, Rob Howes, Roger Clark

**Affiliations:** 1 BioPharmaceuticals R&D, AstraZeneca, Cambridge, UK; 2 Charles River Laboratories, Chesterford Research Park, Saffron Walden, UK; 3 Department of Medicine, University of Cambridge, Cambridge, UK; 4 GSK R&D Tech, Stevenage, UK; 5 GSK R&D, Stevenage, UK; 6 Division of Virology, Department of Pathology, University of Cambridge, Cambridge, UK; 7 Vice Chancellor’s Office, University of Cambridge, Cambridge, UK; 8 School of Clinical Medicine, University of Cambridge, Cambridge, UK

**Keywords:** High-throughput screening, Infectious-disease diagnostics

## Abstract

On 11th March 2020, the UK government announced plans for the scaling of COVID-19 testing, and on 27th March 2020 it was announced that a new alliance of private sector and academic collaborative laboratories were being created to generate the testing capacity required. The Cambridge COVID-19 Testing Centre (CCTC) was established during April 2020 through collaboration between AstraZeneca, GlaxoSmithKline, and the University of Cambridge, with Charles River Laboratories joining the collaboration at the end of July 2020. The CCTC lab operation focussed on the optimised use of automation, introduction of novel technologies and process modelling to enable a testing capacity of 22,000 tests per day. Here we describe the optimisation of the laboratory process through the continued exploitation of internal performance metrics, while introducing new technologies including the Heat Inactivation of clinical samples upon receipt into the laboratory and a Direct to PCR protocol that removed the requirement for the RNA extraction step. We anticipate that these methods will have value in driving continued efficiency and effectiveness within all large scale viral diagnostic testing laboratories.

## Introduction

Following the declaration of SARS-CoV-2 as a pandemic by the World Health Organisation on 11th March 2020^1^, governments across the world announced unprecedented measures to mitigate the spread of the virus through their populations. Alongside the reduction of social contacts and isolation of symptomatic individuals, a key tool in the pandemic response was the expansion of diagnostic facilities to detect the spread of the disease and then to contain it in an effort to mitigate healthcare infrastructure being overwhelmed—a strategy that has proved effective in countries including New Zealand, South Korea, and Iceland, among others^2–7^. In the UK, the Secretary of State for Health announced plans for the creation of a national network of new laboratories (so-called Lighthouse Laboratories) that would rapidly scale-up capacity for delivering RT-qPCR analysis of clinical samples^8^. The Cambridge COVID-19 Testing Centre (CCTC) was established during April 2020 as part of this network, through collaboration between AstraZeneca (AZ), GlaxoSmithKline (GSK), and the University of Cambridge.

The initial challenges of rapidly deploying smaller-scale facilities have been documented by others^9–12^ however the scale of testing required at the CCTC necessitated a different way of thinking to ensure capacity and throughput could be achieved in a relatively small footprint, whilst ensuring the quality of the process was maintained or improved. The CCTC partner groups built on their expertise in automated laboratory screening/profiling processes to achieve this taking the CCTC from concept to operational testing facility within the Anne McLaren Building, Cambridge in just 6 weeks (Supplementary Fig. S1).

The CCTC is unique in many ways to other local and national COVID-19 diagnostic laboratories, as in addition to processing the normal single swab samples, the centre also evolved a process for pooled patient swab samples from the University of Cambridge Asymptomatic Testing Programme for its students^13,14^ and testing of staff samples from the local NHS Trust hospital (Addenbrooke’s Hospital—the largest in the East of England). The centre also employed a dedicated technology development team involved in research activities leading to pioneering innovations for improving the safety, efficiency, robustness, and portability of the COVID-19 screening process.

Initial deployment with a focus on rapidly expanding capacity meant that the CCTC adopted the standard RT-qPCR process already established in many facilities, but from the outset condensed the PCR reaction into a 384-well format, thereby reducing the PCR machine requirement by fourfold. Whilst this assay process delivered high quality in a robust manner there was still room for continued optimisation in terms of efficiency. As the initial volunteer workforce began to return to their home organisations, Charles River Laboratories entered the CCTC collaboration in July 2020, providing a large-scale sustainable scientific workforce to continue diagnostic testing. All partners worked together thereafter to further develop technologies and iteratively improve performance as an analytical facility.

## Results

### Analysing and improving the operational process through modelling

Following the establishment of the facility in May 2020, the focus turned to enhancing our efficiency and effectiveness of operation. The critical first step in this journey was to establish and agree Key Performance Indicators (KPI) setting a baseline against which to measure performance. These KPI focused on four areas:

1. *Quality* In-Process (IP) void rate  ≤ 0.5% of samples voided through process errors in the laboratory
2. *Capacity* The ability to process up to 22,000 samples in a 24-h period
3. *Turn Around Time (TAT)* > 80% of samples having results reported within 24 h of bio-sampling (in response to the target set by the UK Prime Minister)^7,15^.
4. *Safety* No reportable Safety, Health and Environmental incidents of any description.

**Figure 1:**
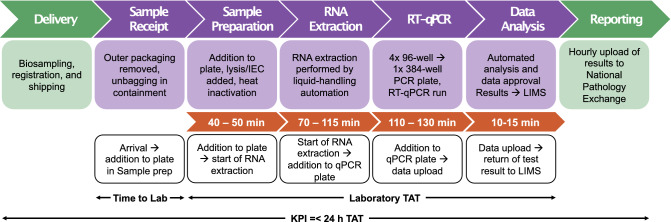
Standard laboratory process showing the journey of a sample from bio-sampling to result. Purified SARS Cov-2 RNA from clinical swab samples is detected by RT-qPCR targeting the ORF1ab gene. Flow diagram at the top describes each step; the internal laboratory procedure shown in purple, and the external processes in green. The time recorded for each step to occur is highlighted in orange. Definition of the Laboratory Information Management System (LIMS) timestamps described in this manuscript are outlined in the white boxes.

In comparison to other testing facilities within the Lighthouse Laboratory Network and globally^9,10^, the CCTC had a relatively constrained footprint of separate rooms within an already operational laboratory facility. With this restricted footprint, process modelling was essential to correctly ascertain the optimal number of automated platforms and staffing deployment to deliver the workflow at each station across the facility (Fig. 1), first basing the model on best estimates. As the laboratory process matured over the first months of operation, the model was refined through the feedback of real-world empirical data, building in more rulesets that served to highlight weaknesses in the logic and iteratively improved the predictive power of the model^16^. The initial CCTC laboratory process was able to achieve a capacity of more than 10,000 samples/day. Over the summer months in 2020, the CCTC strived to double its daily capacity to 22,000 samples/day. We were however conscious that simply adding more staff would not be the most efficient solution to the problem of delivering against our four KPI. Social distancing, both within the laboratory and the wider site, was crucial to maintain, in addition to the understanding that once a certain team size is reached, the addition of further resource can make processes less efficient due to sub-optimal communication and reporting^17,18^.

Our modelling determined that for continuous CCTC operation, the optimal staffing should be as described in Table 1a and equipped with 17 class-II Biological Safety Cabinets (BSC), 11 Beckman Coulter Biomek liquid handlers (i5/i7), and 9 Roche RT-qPCR Light Cycler^®^480 II instruments. We initially deployed two nine-hour shifts of ~ 40 staff, across all days of the week, however after refining our model based on the changes in sample delivery regime to a ‘start/stop’ model, we calculated performance would achieve an average of 17,000 samples/day with a maximum of 20,000 samples/day. This difference from our target capacity was tightly linked to the assumption within our initial modelling that a constant flow of samples would be maintained, providing an endless stream of work during operating hours. In reality, this was rarely achieved due to the varying time of sample delivery to the lab; for example, Testing Sites would perform a large amount of bio-sampling towards the end of each day, and therefore large consignments of samples would be received by the lab at the end of the evening shift, leaving no time for them to be fully processed during that working day.

**Table 1:** Staffing and capacity modelling.

(a) Optimal staffing numbers				
Station	Morning	Evening	Night	
*SP*	30	30	5	
*RNA*	4	4	2	
*PCR*	5	5	2	

(b) Capacity process modelling				
Model includes		Capacity*I*thousands		
RNA extraction	Unbagging in containment	Average	Maximum	Bottleneck
Yes	Yes	21	24	RNA
No	Yes	23	24	Unbagging in containment
No	No	29	30	PCR

(**a**) Optimal staffing numbers as defined by the process modelling described. (**b**) Capacity process modelling predictions—assuming a continuous process aligned to staffing numbers shown in (a); predicted process bottlenecks are highlighted.

*SP *Sample Preparation Team, *RNA *RNA Extraction Team, *PCR *RT-qPCR Team, *Unbagging in containment* removal of sample secondary containment within a BSC.

In our focus on efficiency, we exploited our modelling to identify bottlenecks in our laboratory process and strategically implement improvements on that process. The introduction of a night shift allowed a 24-h operation that avoided in-process samples being held overnight, and therefore our process model could be adapted back from ‘start/stop’ to ‘continuous’—now predicting a maximum operating capacity of 24,000 samples/day (Table 1b).

The modelling continued to highlight a major bottleneck in the process at the RNA extraction step driven by the fixed number of liquid-handling robots in the RNA extraction lab. Removing the requirement for RNA extraction altogether would both reduce the laboratory footprint and make the process more economical, transferring the bottleneck to the labour-intensive step of removing secondary packaging within a BSC. A theoretical removal of the requirement for BSC containment at the stage of secondary packaging, allowing this to occur on the open bench, was predicted to expand our capacity to an average of 29,000 samples/day. Intrinsically linked to capacity the theoretical TAT of a sample was calculated as: 3 h 50 min–5 h 10 min. However empirical data on timings gathered through the initial phase of the CCTC operation showed that our mean end-to-end laboratory processing time was in fact 8 h 35 min.

To address both the capacity and TAT bottlenecks, our technology development focussed towards two key innovations:

1. The removal of RNA extraction (so called Direct to PCR; D2PCR) to create a more economical and efficient process whilst reducing the laboratory footprint.
2. Heat Inactivation of viable samples upon receipt prior to entry into the laboratory environment to circumvent the requirement of BSC containment at the point of secondary packaging removal^19^.

We describe the validation of Heat Inactivation of viral samples at scale, within a separate manuscript currently under preparation, and it is not discussed further here.

### Decreasing turnaround time though assay modification

Alongside the introduction of Heat Inactivation, we also explored experimentally the scope for a Direct to PCR assay (D2PCR) that removed the requirement for RNA extraction shown through our modelling to be a capacity and rate-limiting step due to the physical laboratory space available to accommodate the required liquid-handling robotic platforms and the long run-time of the protocol (70–115 min).

The RNA extraction step of the COVID-19 testing workflow achieves two purposes: first the Guanidinium Isothiocyanate (GITC) content of the RNA extraction lysis buffer serves to inactivate potentially viable virus. At the time of establishing the CCTC, GITC-mediated virus inactivation was the most well understood, and therefore the preferred method^20,21^. Second, RNA extraction purifies and concentrates viral RNA in advance of RT-qPCR detection. However, advances in RT-qPCR reagents lead to the possibility of performing RT-qPCR directly on crude samples, and when coupled with heat inactivation, the very real possibility of removing any RNA extraction step completely. In our workflow, the omission of RNA extraction was calculated to reduce the laboratory TAT on samples by 2 h, whilst simultaneously increasing overall capacity of the laboratory by repurposing staff and facilities into sample receipt and preparation. This D2PCR approach has other significant advantages, in particular a reduction in the use of laboratory consumables, including a 50% reduction in the number of pipette tips, further reducing the cost of the assay, reducing waste and at a point when the global supply-chain for reagents, labware, and equipment could not keep up with demand, facilitating centre operation. Further to this, RNA extraction methods use large amounts of solvents that require bespoke storage and disposal. Use of D2PCR for detection of COVID-19 has been previously demonstrated^22–24^. Herein we present a clinically approved high-throughput methodology, developed using the *Genesig® Real Time PCR COVID-19 High Throughput HT-CE kit V2.0* targeting the same ORF1ab region of SARS-Cov-2 and containing an optimised buffer formulation which overcomes sample-mediated PCR inhibition.

**Figure 2:**
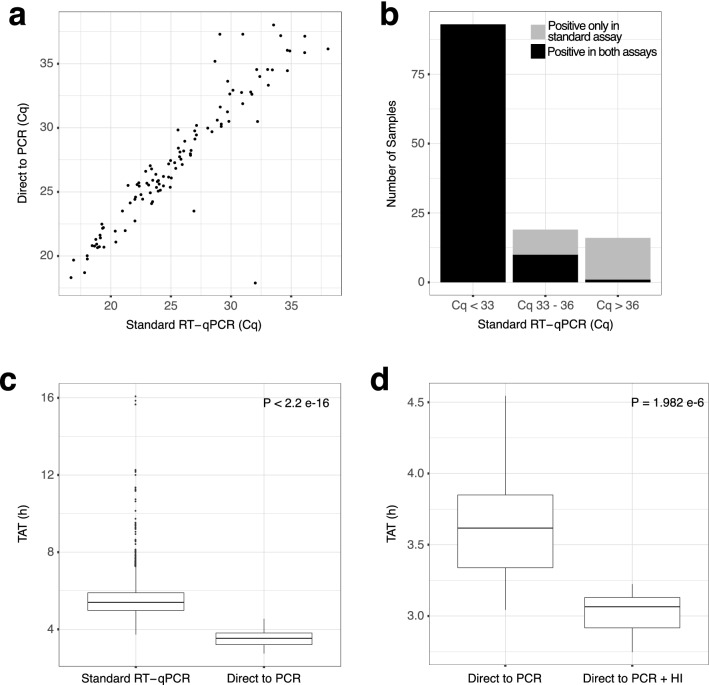
Direct to PCR (D2PCR) concordance data. (**a**) Cq values for samples tested as positive in both D2PCR and standard assay, showing typical increase of 2–4 Cq units with D2PCR. (**b**) Concordance of test results. Samples which tested positive in either the standard assay or the D2PCR assay binned depending on the standard assay Cq. Graph indicates the number of samples tested and the concordance by Cq bin. (**c**) Lab TAT for standard assay (including RNA extraction) compared to D2PCR. For standard test all test results generated within March 2021 are shown, for D2PCR all data from a trial run over 3 days is shown. P value calculated using Wilcox test. (**d**) Lab TAT for D2PCR, comparing Heat Inactivation during the lab process (via thermal cycler) with Heat Inactivation prior to lab entry. P value calculated using Wilcox test. Figure prepared using R with ggplot2 v 3.3.2 [CRAN—Package ggplot2 (r-project.org)].

Validation of the D2PCR process for clinical testing was carried out as described in the methods, comparing the D2PCR method directly with the standard RNA extraction-based protocol. All samples with a positive result in the standard assay, with a Cq value of 33 or lower, tested positive using the D2PCR assay (Fig. 2a,b) with a concordance rate of 100%. For weaker positive samples with Cq values of between 33 and 36, the concordance rate was 52.6%, while very weak positives (Cq > 36 in standard assay) were mostly not detected (6.25% positive to positive detection rate) (Fig. 2b). This shift in the limit of detection was expected based on the D2PCR using fourfold less RNA input than the standard assay (due to the lack of concentration effect from RNA extraction), as well as some likely impact of interference on PCR efficiency from the crude sample matrix. The significance of individuals with high Cq positive results within wider public health response is a matter of current debate, however it is likely that this is reflective of low-level viral RNA relating to individuals early or late in their course of infection, even when they are no longer infectious to others^25^. Data were reviewed by our Clinical Lead and wider Public Health England boards, where it was agreed that the reduced sensitivity at extremely low viral-load levels was acceptable and the D2PCR methodology was formally approved for clinical sample testing.

Beyond the benefits of cost, reagents, footprint, and waste reduction we assessed the effect that the D2PCR method would have on TAT in our operational laboratory. When we examined the laboratory TAT in this pilot study, we found the samples had a median time to completion of 3 h 32 min. When compared against all other samples processed in the same month (March 2021) using the standard laboratory process, we found this represented a median time saving of 1 h 52 min (Fig. 2c). As mentioned above, we have also developed and deployed a method for heat inactivation of samples before they enter the lab. The combination of D2PCR with heat inactivation led to a further median in lab time saving of 33 min (Fig. 2d).

### Exploiting operational data using an informatics-based approach

Across the Lighthouse Laboratory Network, the end-to-end laboratory process was supported by a Laboratory Information Management System (LIMS) that provided the backbone of data management within the labs. A LIMS is fundamental to management of data flow within a testing laboratory such as the CCTC dealing with several thousand samples per day—mapping the lifecycle of individual patient samples as they progress through the physical laboratory process. As patient samples undergo transformation and compression from individual vials to multiwell microtitre plates, onwards through plate-plate transfers, the LIMS records that lineage and captures various timestamps throughout the process (Fig. 1). These timestamps are not only imperative to the detailed tracking of individual samples based on an anonymised barcode, but also provide a rich data set with which to view performance of the process in a real-time fashion. However due to the nature of the LIMS environment and requirement to ensure change-control was centralised across the lab network, the ability to be agile with development of aligned local IT tools to exploit the data was crucial.

The combination of a core *Customisable Off The Shelf* product aligned with associated tools developed in an agile methodology to bring immediate benefit in exploitation of operational data is well proven to deliver results quickly^26^. To this end we targeted two user bases who we thought would be best placed to interact with these data—delivering tools appropriate for each (Supplementary Fig. S2). Firstly, we provided the laboratory management team with data regarding past performance to examine areas for improvement (Centre Performance Overview tool & Shift Lead dashboard; Supplementary Figs. S3, S4). Secondly, we provided the scientists in the laboratory with dashboards to enable real-time feedback on performance against key performance indicators (Supplementary Fig. S5).

### Visualization of retrospective and real-time operational data

The *Centre Performance Overview* tool provides a retrospective view of the laboratory TAT, broken down by station and time of day, with multiple interactive methods of viewing the data. Visualisation of where and when samples were being delayed focussed our attention, enabling adoption of working practices aimed at reducing any bottleneck. Key process inefficiencies were quickly identified at the handovers between stations and shifts, which could be addressed through process change without requiring significant modification to the SOPs for the individual workstations. Visualising TAT data in this fashion also highlighted the importance of maintaining staff levels at defined minimum numbers in certain teams to avoid new process bottlenecks arising—ensuring that the CCTC management team could work with operational Shift Leads to rebalance resource appropriately. Viewing the flow of data through the centre in this holistic fashion also enabled informed discussion with the upstream Department of Health & Social Care (DHSC) logistics teams around optimal sample delivery schedules to achieve the best TAT.

To complement the retrospective executive view, it was crucial to provide real-time, non-interactive dashboards, providing quantitative feedback to the teams on each shift regarding their performance in real-time. This approach has previously been documented for the receipt of samples and result reporting at an in-house hospital diagnostic facility, with operational improvements made considering this visualisation of data, but our efforts concentrated on the laboratory process^27^. Information was broken down for each station in three streams (Supplementary Figs. S4, S5):

1. The incoming workload from the previous station to prepare reagents and equipment.
2. A real-time view of the workload at the station, where plates experiencing a delay beyond expected process time are highlighted in red.
3. A 24-h analysis of the day’s performance, allowing instant feedback.

The visualisation of current workflow was particularly important in stations containing automated platforms, where dashboards were configured to highlight automation end-times so that plates/data could be expedited to the next step. These tools were specifically designed to ensure completed plates were swiftly moved to the next stage, striving towards a steady flow through the lab.

**Figure 3:**
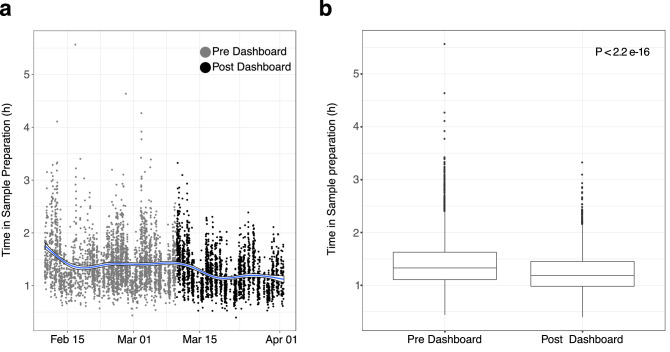
Using informatics dashboards to improve process efficiency in Sample Preparation. (**a**) Each point represents a single microplate where the x axis describes creation time within LIMS and y axis the total time spent within the Sample Preparation step. Grey points are before the introduction of the in-lab dashboard; Black points are post introduction. Blue line is a regression calculated using a generalized additive model with the SE shown. (**b**) Box plot of data from (**a**). P value from Wilcox test. Figure prepared using R with ggplot2 v 3.3.2 [CRAN—Package ggplot2 (r-project.org)].

To investigate any effect on TAT through use of these data management tools, we monitored the time a sample plate spent within Sample Preparation before and after implementation. Here we observed a notable decrease in the time a sample spent at this stage within a few days of the introduction (Fig. 3) and an overall significant reduction was observed across the time points studied with a median reduction of 8.46 min (10.6% improvement). In particular, the number of plates spending over three hours in Sample Preparation were substantially reduced through introduction of this tool, indicating that staff are not necessarily working at a higher speed, but rather that delayed plates are being identified and expedited, thus reducing the variance in time spent at this station.

### Reviewing CCTC performance

**Figure 4:**
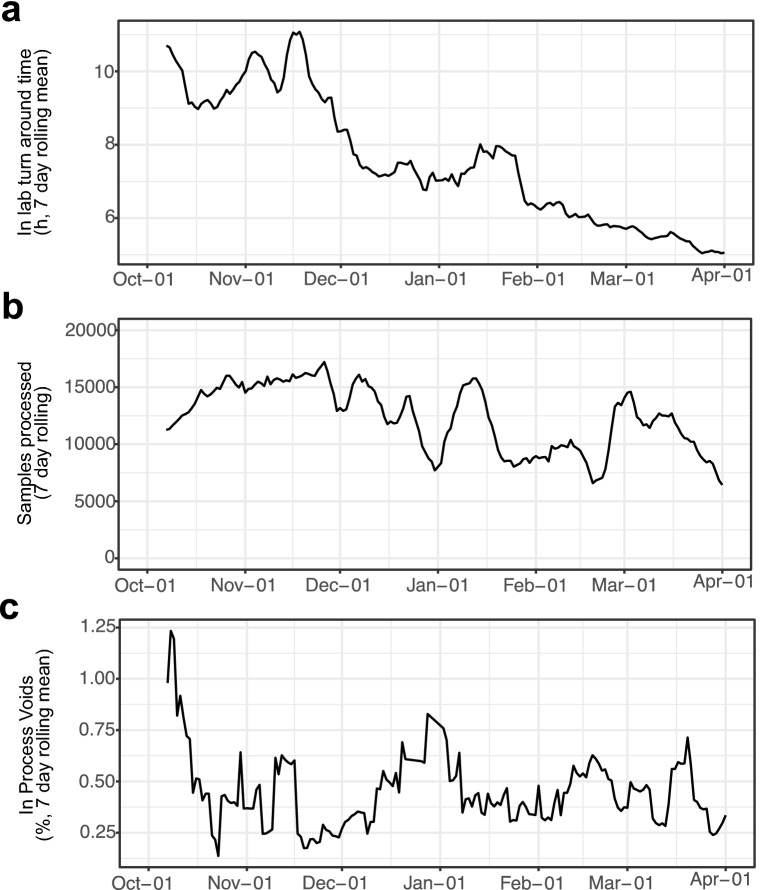
Lab TAT, samples processed, and quality control of the CCTC from October 2020–March 2021. (**a**) 7-day rolling mean of the lab TAT. (**b**) 7-day rolling mean of the total daily sample number processed. (**c**) 7-day rolling mean of the in-process voids within the lab. Figure prepared using R with ggplot2 v 3.3.2 [CRAN—Package ggplot2 (r-project.org)].

To review performance of the CCTC against our established KPI, we plotted the seven-day rolling mean of the process timing data collected to quantify our progress (Fig. 4). The laboratory TAT understandably had a direct relation to the number of samples processed, however, after implementing the strategies described in our paper (minus D2PCR) the CCTC sustained a high workload with peaks in both January 2021 and March 2021 without an aligned detrimental effect on TAT. Indeed, our mean TAT for March–April 2021 was below 6 h, in line with the theoretical time for the process of 3 h 50 min–5 h 10 min (Fig. 1). This focus on exploitation of our operational data to continually drive efficiency of process has led to the CCTC consistently achieving its KPI of > 80% of samples processed within 24 h (achieved on > 73% of days in 2021). Heat Inactivation upon receipt was formally adopted into the CCTC process in early February 2021 and quickly showed positive impact by helping to smooth the flow of samples from receipt into the lab – along with the other advantages described earlier.

Whilst our efforts in reducing laboratory TAT had an observable impact, this would be counter-productive if these improvements were detrimental to quality. The seven-day rolling mean of the centre’s In-Process (IP) voids is plotted in Fig. 4c and shows that there has been no increase in IP voids throughout our drive towards efficiency and effectiveness of process (the average rate remaining below our target KPI at 0.45%).

## Discussion

A comprehensive clinical testing infrastructure is a critical component in combatting disease outbreaks. At the CCTC we deployed a functional industrial scale diagnostics facility and have continued to strive for improved efficiency and effectiveness of our process, to ensure that the centre delivered its objectives to as high a standard as possible.

In the establishment and continued management of any process in an efficient manner, it is important to ensure that the baseline for establishing what ‘good’ looks like is agreed by all stakeholders. Of equal importance is that all those delivering the process understand these baseline metrics and how their own efforts can impact them (both positively and negatively). Our efforts in progression of the CCTC from initial establishment to fully optimised operation has been underpinned by our ability to use internal process metrics to highlight bottlenecks and improve consistency of flow through our end-to-end process. From point of sample receipt into the CCTC we are able to track progress of samples through each of the different ‘stations’ (Fig. 1) and display that progress in almost real-time through use of our comprehensive suite of dashboards (Supplementary Figs. S4, S5). These data drawn from several underlying sources were computationally wrangled into a consistent set of data objects which could then be displayed to various consumers of that data, ensuring that display was tailored to their needs. Through regular management review of operational performance data and translation of that data into operational improvements through management on the ground, the CCTC was able to exploit those data in near real-time to enable the CCTC to meet all our KPI and further drive efficiency of our processes.

**Figure 5:**
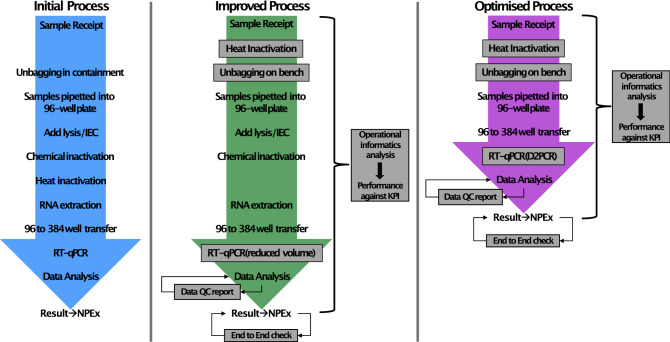
Evolution of the CCTC laboratory process. Evolution from initial standard process to fully optimised (incorporating both Heat Inactivation upon receipt and D2PCR format). Key changes from the initial process are shown in grey boxes—including physical laboratory steps along with alignment of data QC tools and continuous Operational informatics analysis.

In this report we have documented the informatics tools that we have used to attain our objectives on TAT and quality, whilst capacity modelling, heat inactivation upon receipt, and D2PCR have exemplified our efforts to reduce the footprint, increase the safety of our process, lower our dependence on multiple supply chains, and reduce the burden of labour-intensive steps, making a more effective and economical diagnostics facility. In addition to the optimisation noted above we further optimised the assay set up and quality control as detailed in the Supplemental Information including changes in assay volume, instrument performance tracking and data integrity. This has resulted in what we believe is the optimised process that future pathogen screening laboratories can follow (Fig. 5).

In the first year of operation the CCTC processed 3,264,596 clinical samples. Over this time, continuous process monitoring has been applied to highlight areas of focus for technology advancement including heat inactivation and D2PCR, with the impact of those advancements being observed in real-time In situations such as emerging pandemic threats, the speed at which a highly efficient response can be implemented is key to minimising impact to human health and society. Adoption of the processes and improvements described here, as learned through the COVID-19 pandemic, will enhance efficiency of process and effectiveness of delivery for future clinical diagnostics facilities.

## Methods

### Ethics statement

This study was conducted as part of the Lighthouse Laboratories surveillance for COVID-19 infections set up under the auspices of Section 251 of the National Health Service Act 2006 and/or Regulation 3 of The Health Service (Control of Patient Information) Regulations 2002. The study therefore did not require individual patient consent or ethical approval. No Patient Identifiable information (PII) was received by the Centre. Authors only had access to anonymised data in the form of sample barcodes. Approval for the operation of the CCTC and improvements to the procedures used therein was granted by the Department for Health and Social Care under the emergency provisions granted by the Secretary of State under Section 251 of the National Health Service Act.

### Capacity modelling

A stochastic simulation of the sample preparation and screening processing steps was developed by Dr Michael Allen (University of Exeter Medical School) using the *SymPy* library associated with the Python development environment^16^. The simulation was developed to consider the processing times at each step with associated human and equipment resources. Variation in time for either human or equipment related processing was applied using a triangular distribution. Workforce break times, shift routines and estimates for equipment breaking down were incorporated into the model. Simulations were run over 30 iterations and the results aggregated to provide an output for capacity, test-sample queuing, and resource utilisation at each step and for the overall process. The simulation data was used to identify potential processing bottlenecks so that solutions could be explored by adjust the screening process and/or applied human and equipment resources.

### SARS-Cov-2 RT-qPCR diagnostic assay

Methodology for the standard SARS Cov-2 diagnostic test used at the CCTC is described in detail in the Supplementary Information, where we have presented the Standard Operating Procedures in abridged form, focusing on transferable aspects and removing any CCTC-specifics that would be irrelevant to other laboratories. In brief, clinical oropharyngeal/nasopharyngeal (OP/NP) samples were received at the CCTC in leakproof UN3373 packaging containing a screw capped sample tube with a swab stick immersed in viral transport medium (VTM). OP/NP sab vials were unpacked and racked within a microbiological safety cabinet and following Containment Level 2 precautions. For each sample, 200 µl of VTM was transferred to a 96 deep well sample plate, followed by the addition of RNA extraction lysis buffer, Proteinase K and Internal Extraction Control (IEC) RNA. The sample plate was sealed and double contained, and placed at 65 °C for 10 min for heat inactivation of viable SARS-Cov-2, followed by incubation at room temperature for 10 min. RNA was extracted using the RNAdvance Viral Kit (C63510) on Biomek i5 or i7 automated platforms from Beckman Coulter. Viral RNA was eluted in nuclease-free water and then used for RT-qPCR using the Genesig Real-Time COVID-19 PCR High Throughput assay kit (Primer Design Ltd, Geneisg Z-Path-COVID-19-CE HT1.0) as described by the manufacturer except that 10 µl (i.e. half) reaction volumes were used. RT-PCR reactions were prepared in white, 384-well LightCycler 480 Multiwell Plates (Roche #04729749001), 6 µl RT-qPCR master mix was added using a ThermoFisher Multidrop Combi and 4 μl extracted RNA sample was added using an Agilent Bravo. RT-qPCR reactions were run on a Light Cycler 480 II and data was analysed using a bespoke algorithm (FastFinder software, UgenTec) to define Cq values and assign test results following interpretation of controls (positive, negative, IEC) and according to a defined decision tree.

### Direct to PCR (D2PCR) validation

In the D2PCR assay, the standard process described previously is shortened by omission of the initial sample lysis and RNA extraction steps. Instead, 100 μl of each OP/NP swab sample was transferred to a *Hard-Shell Low-Profile Skirted 96-Well PCR Plate* (Biorad #HSP9601), sealed with an *Aluminium Foil Seal* (Beckman Coulter #538619) and heated in a *PCR Max AlphaCycler*, set to 65 °C for 20 min. Heat inactivation could also be performed at the stage of sample receipt, which has the advantage of allowing subsequent steps to be performed without Containment Level 2 working, or by another suitable method that achieves the required heat exposure for viral inactivation prior to PCR set-up outside of biological containment. All equipment used for heat inactivation should be suitably calibrated to verify the required heating of samples^28^. RT-PCR reactions were prepared in *White, 384-well LightCycler 480 Multiwell Plates* (Roche #04729749001) by sequential addition of 3.5 μl of *Genesig Real Time PCR COVID-19 High Throughput HT-CE kit V2.0* (Genesig #Z-Path-COVID-19-CE) PCR master mix using a *ThermoFisher Multidrop Combi*, followed by 2.5 μl of a 50-fold dilution of IEC in nuclease-free water using an *Agilent Bravo* (omitting positive and negative control wells where IEC was not included). The RT-qPCR assay was initiated through addition of 4 μl heat inactivated sample to the 384-well microplate containing PCR master mix and IEC using an *Agilent Bravo*, and loading of the 384-well microplate into a *Roche LightCycler 480 II*.

Initial D2PCR experiments were performed using OP/NP swab samples with known SARS-CoV-2 status (samples already tested using the approved CCTC standard laboratory process) to determine suitable conditions for heat inactivation of potential viable SARS-Cov-2 and RT-qPCR set-up. In the absence of an RNA extraction step, the IEC was included to confirm successful RT-PCR in every sample (therefore highlighting any unexpected sample-mediated reaction inhibition). IEC was added to PCR master mix in 384-well microplates to avoid weak or variable IEC signals that are seen when IEC is added to samples themselves, likely a result of RNA degradation.

Validation of the D2PCR methodology for clinical testing was carried out by testing of 1100 OP/NP swab samples in parallel to the standard RNA extraction-based methodology, testing over three separate days (Fig. 2).

### Operational informatics

To enable rapid development, we based our solution around an RStudio connect server deployed on a virtual machine connected to a data lake containing output from our LIMS. Architecture of the system was set up as shown in Supplementary Fig. S2.

Automated queries generated reports from the LIMS containing all active and recently completed (archived) plates on a 15–30 min cycle. These reports were output in CSV format and deposited into an Azure file share, forming the basis of our data lake.

The Azure file share was mapped to a virtual machine running RStudio connect. A series of scheduled R markdown scripts regularly imported the CSV reports, calculated required statistics, and appended the new data to existing RDS files which contained all the processed data required.

Data was visualised and exploited via several applications coded in *Shiny* (an RStudio package) which read the RDS files and provided web-based visualisations (Supplementary Figs. S3, S4, S5).

## Supplementary Information

Supplementary Information 1.

Supplementary Information 2.
