# Supplementary material for: Improving the efficiency and effectiveness of an industrial SARS-CoV-2 diagnostic facility
Source: Sci Rep. 2022 Feb 24;12:3114. doi: 10.1038/s41598-022-06873-6 (PMC8873195; doi:10.1038/s41598-022-06873-6)
Supplement: Supplementary file 1 — Supplementary Information 1. [file 41598_2022_6873_MOESM1_ESM.docx]

**Figure S1. (Timeline for establishment of the CCTC)
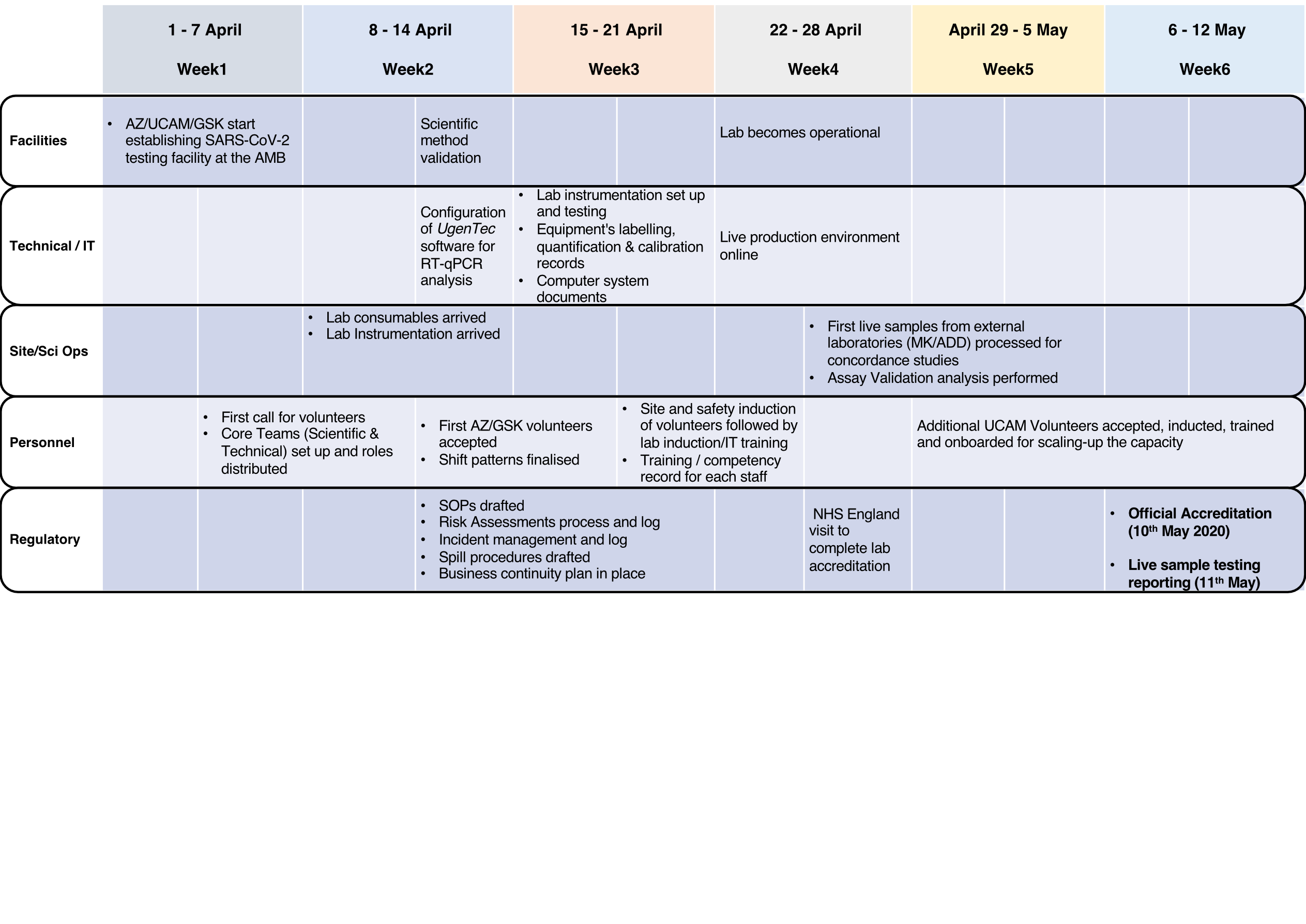
**

From concept to fully functional laboratory - ready for delivering SARS-CoV-2 diagnostic results – took approximately six weeks. The CCTC received its operational approval on 10th May 2020 and first reported diagnostic results on 11th May 2020.

**Figure S2. (Operational Informatics system architecture)**


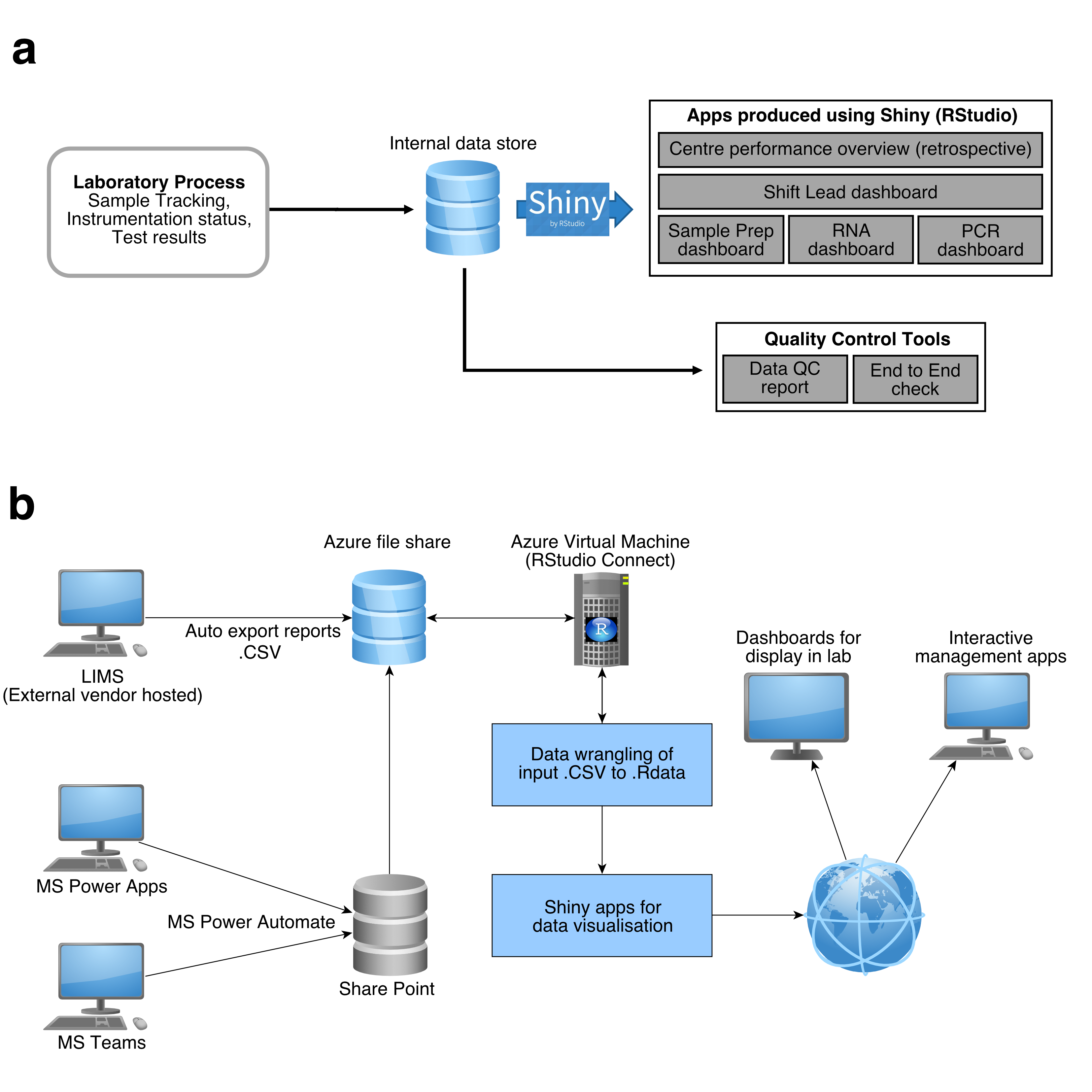


**(a)** High-level architecture for exploitation of CCTC operational data. Various dashboards (delivered via Shiny Applications) are shown along with operational QC tools running across data extracted from the LIMS coupled with other local data sources. **(b)** Lower-level architecture; based around an RStudio connect server which ingested data from multiple sources and output graphical representations as either interactive displays, or non-interactive dashboards to present real time performance data to the staff on shift. Figure prepared using yED 3.21.1 [yEd - Graph Editor (yworks.com)](https://protect-de.mimecast.com/s/xXyrCnR2olCXGXM5DHJiUAE?domain=yworks.com).

**Figure S3. (Centre Performance Overview)**

**
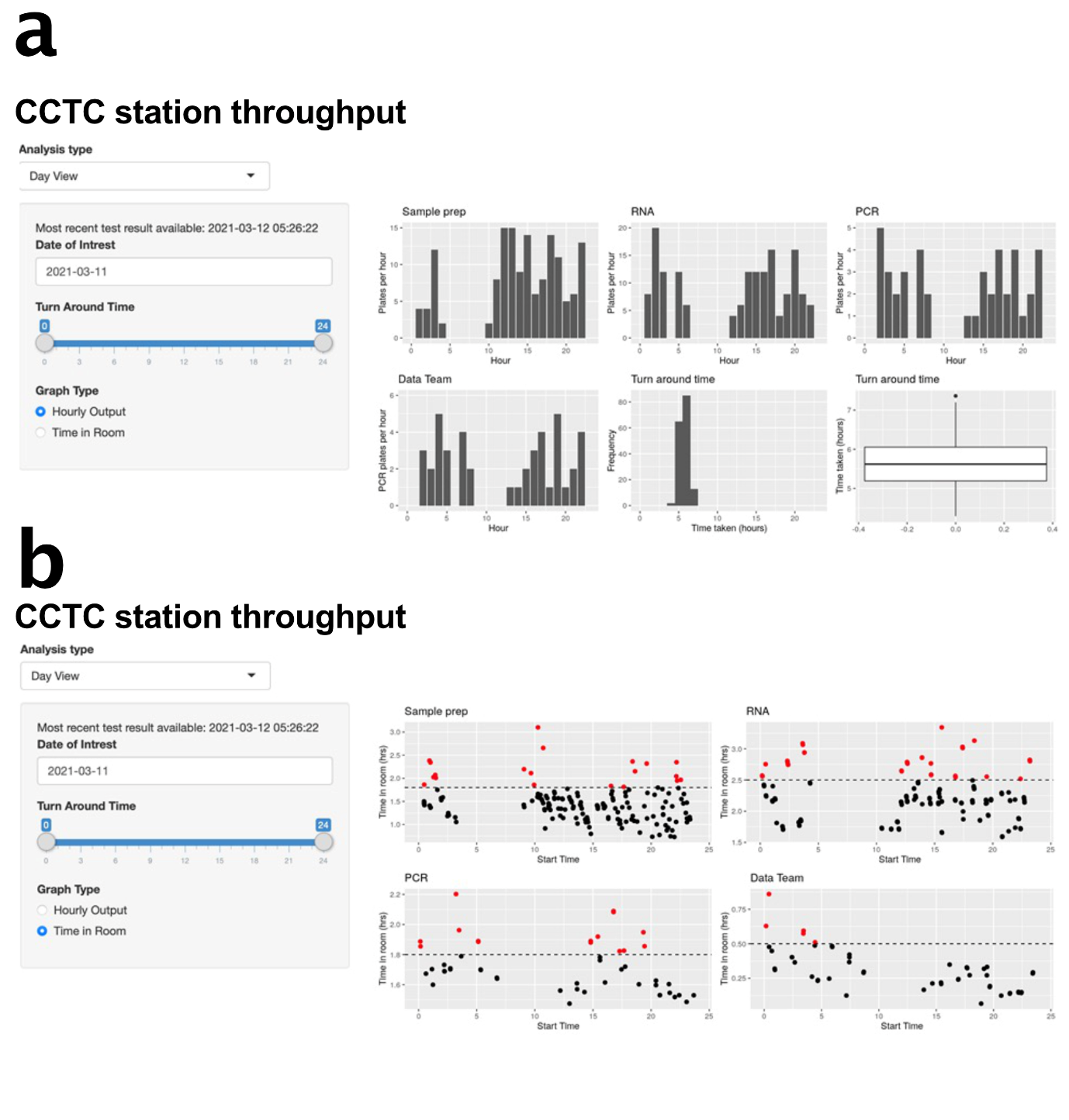
**

Example screenshot of the *Centre Performance Overview* tool. **(a)** Plates per hour shown across the various stations for a selected day. Distribution of plates by laboratory Turn Around Time (TAT) is also shown as both histogram and box & whisker plot. **(b)** TAT for plates at each of the stations. Each point represents a single 96-well plate, where time in room calculated as the time of ***plate creation*** to the plate being ***archived in LIMS at the subsequent station***. Points show as red once they progress beyond the target time for completion at a given station.

**Figure S4. (Shift Lead dashboard)**

**
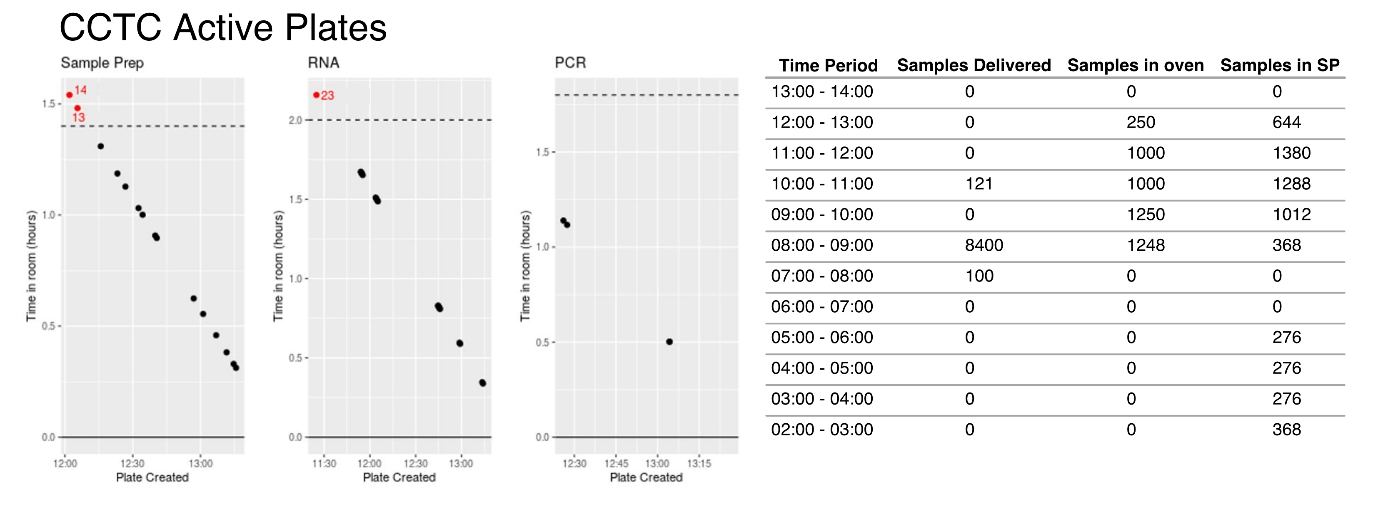
**

Example screenshot of the *Shift Lead dashboard*; providing near real-time operational management oversight of progress. The current workflows in each of the wet-lab stations of Sample Preparation, RNA extraction and PCR are shown. Delayed plates shown in red with the plate barcodes highlighted. The right-hand side shows the timing and number of samples received at Sample Receipt (captured by a Microsoft Power App). The number of samples processed through the ovens utilised for upfront heat inactivation (captured by an excel document hosted on Microsoft Teams), and samples received in the Sample Preparation laboratory (captured by a Microsoft Power App).

**Figure S5. (Sample Preparation dashboard)**


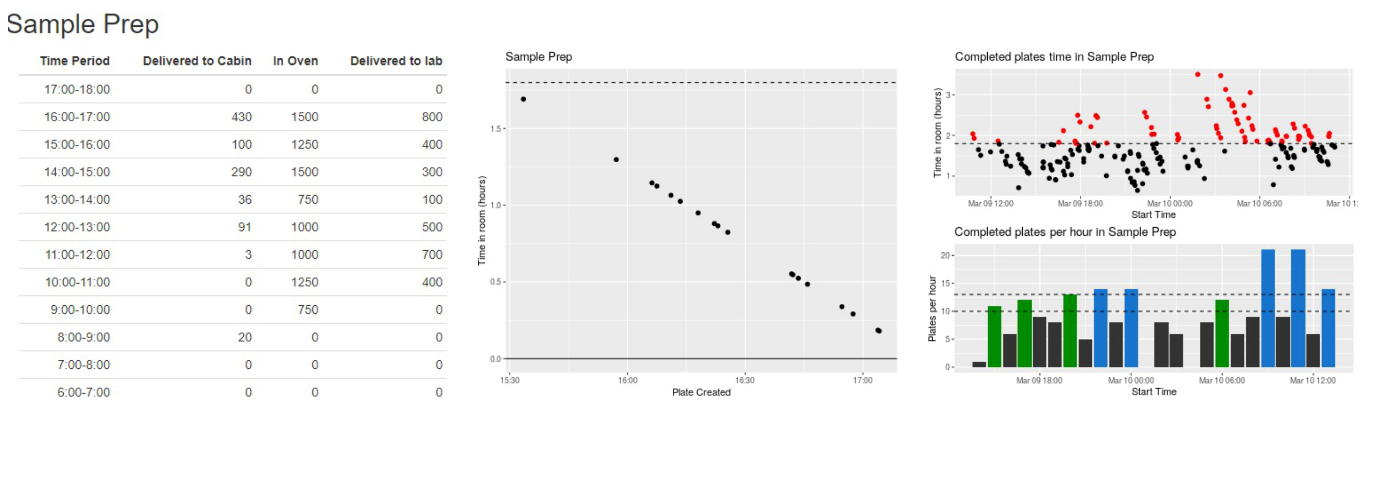


Example screenshot of the *Sample Preparation dashboard*; three streams of information are visualised. Upcoming work - the number of samples received at sample receipt, the number of samples processed through the ovens and the number of samples delivered to the Sample Preparation laboratory. Current work – the creation timestamps of viral preparation plates that are active in LIMS and have not yet been archived at the next station. Performance review – A 24-hour window of the Sample Preparation station performance by creation timestamp of plates and the ‘time in room’ before they are archived, and several plates per hour, distributed by creation timestamp. Similar dashboards were utilised across RNA and PCR stations.

**Figure S6. (Overview of the standard workflow at the CCTC)**


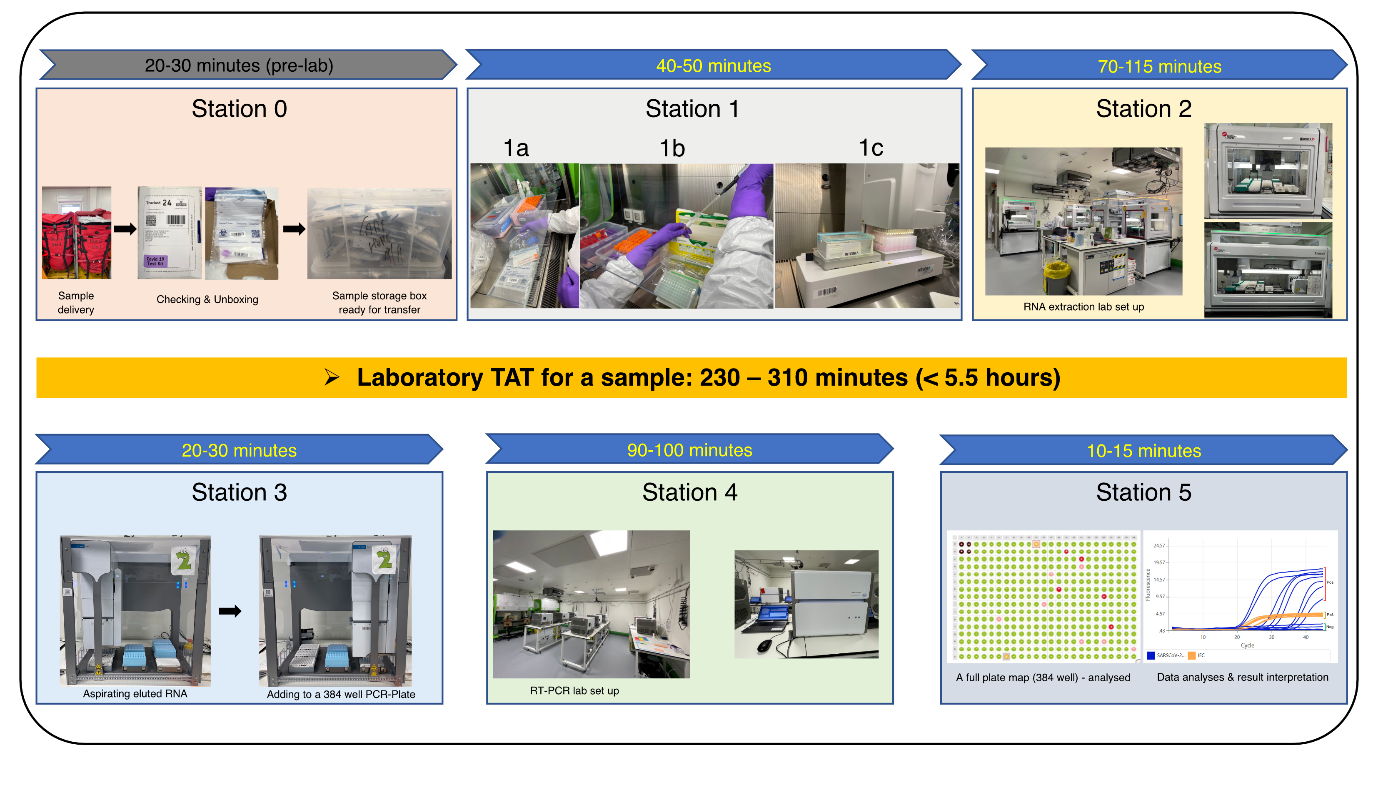


A pictorial overview of the assay to detect SARS-CoV-2 in OP/NP swab samples. Except for Station 0 and 5, all stations were in separate labs within the Anne McLaren Building (AMB).

**Station 0:** OP/NP swab samples received in UN3733 packaging, opened, and examined for leaks.

**Station 1 (Sample Prep labs):** Sample tubes further screened on acceptance criteria, unbagged, racked at Station 1a. OP/NP swab solutions manually pipetted into a 96-well plate at Station 1b and inactivated by adding a buffer mix to the plate at Station 1c using an *Integra ViaFlo* 96 channel pipette, followed by heat inactivation at 65ºC for 10 minutes.

**Station 2 (RNA extraction labs):** Automated liquid-handling platforms (*Beckman Coulter Biomek i5* / *i7*) perform magnetic-bead based RNA extraction handling up to three 96-well sample plates at a time.

**Station 3 (Pre-PCR lab):** Automated liquid-handling platforms (*Agilent Bravo*) transfer RNA samples, consolidating up to four 96-well plates into one 384-well pre-prepared PCR plate.

**Station 4 (RT-PCR labs):** Positive Control Template (PCT) added to the designated wells of the PCR plate and RT-qPCR run on a Roche LightCycler480.

**Station 5 (Remote data teams):** Following curve analysis via *UgenTec FastFinder* data was quality checked, approved and results authorised as “Positive (Pos.)”, “Negative (Neg.)” or “Void” through comparison to the reference curves. Results then reported to the National Pathology Exchange (NPEx) system, utilised for clinical result management in the NHS.

**Figure S7. (Data QC Report)**

**
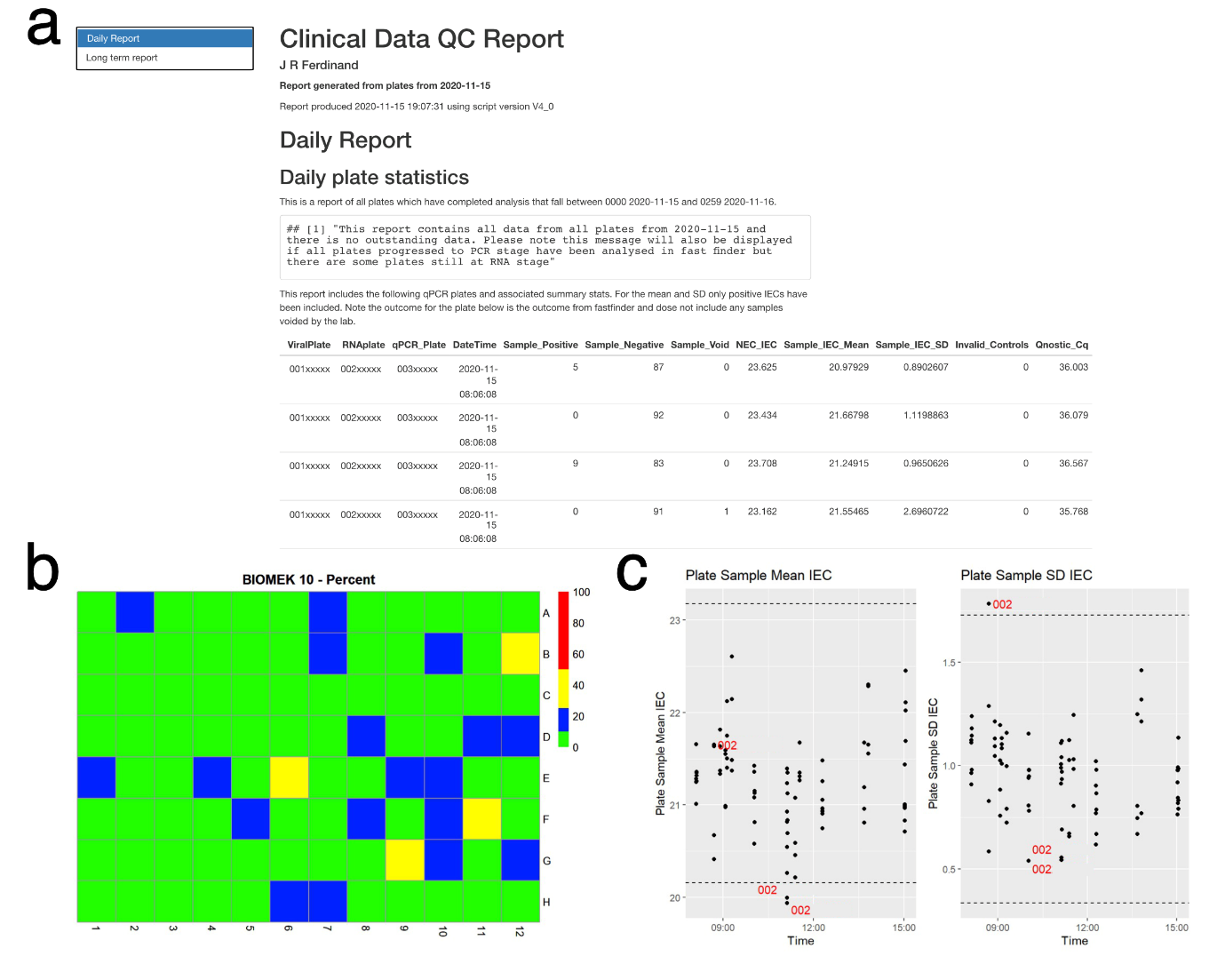
**

Output received from running the Data QC report. **(a)** Mean IEC is calculated for each plate generated in Sample Preparation and the standard deviation calculated. *Qnostic_Cq* is the *ORF1ab* gene Cq of the in-process SARS-CoV-2 positive control developed by Qnostics. **(b)** Heatmap showing percentage of samples that show >= 2SD from the daily Mean IEC Cq. Green: <5% of samples, Blue: <20%, Yellow: <50%, Red: >50% of samples. **(c)** Mean IEC across each plate is plotted against time of day to monitor drift in performance over that day. **(d)** SD of IEC Cq across each plate is plotted against time of day.

**Establishment of the CCTC initial process**

All Standard Operating Procedures (SOP) referred to below can be found in the associated *Abridged SOP* document published alongside this Supplementary Information.

**Setup of the initial standard workflow at the CCTC**

The CCTC was established at the AMB, a newly built high specification building at the University of Cambridge. AMB’s design with self-contained laboratories with integrated air handling system allowed us to compartmentalise the end-to-end process, with separate labs for each of sample receipt, RNA extraction and PCR to avoid cross-contamination of reagents. We were able to keep a physical barrier between labs handling the viable virus from “clean” labs with chemical and heat inactivated non-viable viral material (**Fig. S6**).

OP/NP swab samples from various sources (Home test kits/Care Homes/Regional Testing Centre) across the region were delivered to sample receipt at Station 0 of the centre. Samples arrived in UN3373 packaging, sealed in a MedDx FS95 /C5ADR safety bag. The bag contains a double contained screw capped sample tube with a unique barcode matched to patient identity and swab stick immersed in the viral transport medium (VTM). The boxes are checked against receipt criteria such as external leaking, unpacked on passing the check, and circa 90-100 bags are collected in a transparent sample receipt box that is passed onto the next station (**Fig. S6**, **Station 0;** **SOP-CB-01**).

In the Sample Preparation laboratories (Station 1), all activities are performed inside Class-II biological safety cabinets (BSCs) by trained scientists operating under strict SOP wearing full PPE (**SOP-CB-02**). all steps are performed inside a class-II biological safety cabinets (BSCs) by well-trained scientists operating under strict SOP guidelines wearing full PPE. At Station 1a, the FS95 bags are cut open to further check the quality of the swab sample, for example whether sample tubes are correctly barcoded, to ensure that there is no leakage inside the inner secondary bag, and that the sample surpasses quality criteria (there is a swab and adequate VTM volume). Sample tubes are then unbagged, racked (n=46), and stored at 4 ºC in a plastic box. The box is then wiped down with 1:10 (v:v) Distel and 70% (v:v) ethanol to minimise risk of contamination before being taken out of the BSC.

Two boxes with racked samples (n=92) are then moved to a separate BSC at Station 1b, where each sample barcode is scanned, registered and assigned to a well on an exclusive viral plate ID map in *Brooks LIMfinity*, a Laboratory Information Management System (LIMS). Subsequently, each sample (200 µl VTM) is manually pipetted into the designated well of the 96-deep well plate with each plate containing 92 samples and four controls. Viral inactivation takes place at Station 1c, where the “all-in-one lysis mix” (for components of the mixture see **SOP-CB-02**) is added using a semi-automated pipette channel, *Integra ViaFlo96*, and incubated for 20 minutes; first 10 minutes in the oven at 65 ºC to heat inactivate (HI) the virus followed by 10 minutes at room temperature (**Fig. S6**, **Station 1; SOP-CB-02**). The heat inactivated plate is then transferred to the Station 2. Any sample examined at Station 1a that does not qualify for downstream processing is voided inside a BSC and recorded on a void tool app that reads the barcode of the sample and accordingly updates LIMS.

In the RNA labs (Station 2), extraction of viral RNA from up to three 96-deep well swab sample plates are performed using Beckman RNAdvance Viral kit components using the automated robotic liquid handling systems, Biomek i5 or Biomek i7 from Beckman Coulter (**Fig. S6, Station2**; **SOP-CB-03**). All RNA extraction and robotics protocols are conducted as per manufacturers guidelines. To keep track of samples in the end-to-end process, the sample plate ID and linked eluted RNA plate ID are registered together in LIMS for effective pairing. The eluted 96-well RNA plate is then transferred to the next station, Station 3.

In the pre-PCR lab (Station 3), the COVID-19 Genesig Real-Time PCR assay kit is used for the Reverse Transcription-quantitative Polymerase Chain Reaction (RT-qPCR), in a 384-well format. Multi Drop combi (ThermoScientific) is used to dispense the master mix into PCR plates, this work is conducted in a BSC to avoid contamination. Following this, nuclease free water is manually added to No Template Control (NTC) wells. These PCR ready plates are sealed and stored at 4 ºC for up to a maximum of 6 hours in dark before RNA addition. The Bravo automated liquid handling platform (Agilent) is used to stamp four 96-well plates into one 384-well plate as per the pre-aligned plate-map after the RNA plates are received (**Fig. S6, Station 3**; **SOP-CB-05**). The PCR plate ID and paired RNA plates IDs are then merged in LIMS. PCR plates now progress to Station 4.

In the RT-qPCR lab (Station 4), a Positive Control Template (PCT) is finally added to the designated wells of the PCR plate. PCT preparation and addition is carried out in in a BSC to prevent cross contamination and ensure assay integrity. The PCR plate, after a short centrifugation step, is loaded onto a LightCycler 480 (from Roche) using predefined programme that takes 85 minutes (**Fig. S6, Station 4**; **SOP-CB-06**). The open reading frame1ab (ORF1ab) gene is the target genomic region for amplification and detection of SARS-CoV-2 in this single target assay. Once the run is completed, the file generated with result data (.ixo file) is transferred to the shared network folder for QC analysis and validation by the data team.

Throughout the steps in the laboratory process, instrument identifiers are recorded to enable full tracking and retrospective analysis of any data trends.

The Data team (Station 5) utilise the *FastFinder* software from *UgenTec* to perform a quality check and analyse auto-generated data to detect presence/absence of SARS-CoV-2 virus ribonucleic acid in the swab samples based on Cq values using defined decision trees (**Fig. S6**, **Station 5**; **SOP-CB-17**). Results are further double-checked and approved by the data team lead/shift lead and populated back into the LIMS, before finally being released to the NHS server by uploading on the National Pathology Exchange (NPEx) system.

**Enhancing the CCTC Data Analytics**

**Data QC Report**

Cq of the IEC (present in every sample tested) was downloaded from our LIMS along with the test results and an R markdown script deployed to interrogate the data leading to the production of a Data QC report written in HTML.

The report is split into multiple sections (**Fig. S7**). Initially examining the performance of the IEC across all plates from the current day and comparing to the previous three days. Any plate with a mean IEC outside of the previous three days mean +/- 2SD is flagged for further investigation. Typically, one would expect approximately 5% of plates to exhibit an IEC Cq outside of this range (assuming a normal distribution), however a larger number of plates falling into this category flags a potential laboratory-based issue requiring further investigation.

The report goes on to highlight individual instrument performance, presenting the output as a heatmap using a plate layout (96-well format for all pre-PCR steps, 384-well for PCR) and coloured using a traffic light system where green is no violations and red is >50% of the samples in that location have been scored with an IEC Cq outside of the designated acceptable range.

The final section of the Data QC report looks at long-term trends within the centre (including tracking of reagent batches).

**End to End check**

Due to the rapid creation of the Lighthouse Laboratory Network, it was necessary to align with existing NHS test reporting infrastructure to ensure the labs could push result data correctly (namely to the National Pathology Exchange; NPEx). Our LIMS was set to produce an hourly report of all new test results (aligned against barcode) that was automatically exported as a CSV file to our secure Azure file share and subsequently manually uploaded to NPEx.

Scope of the testing laboratory ended with the transfer of this data to NPEx and this service was then responsible for rapidly releasing those test results to the relevant patients. The manual upload process provided an important final checkpoint that enabled us to halt the release of any data in case of any quality concerns, however the loose integration with the LIMS also introduced a weakness due to the reliance on a manual step (data reports could potentially be missed, archived without ever being sent to NPEx or dropped through loss of connection to the NPEx system).

To mitigate these risks, we developed a comprehensive *End to End check* informatics tool, which became integrated into our process at the end of each shift (3 times per day). This tool took as an input a manually created test results report for all results created in the lab within the previous 72 hours. The tool automatically downloaded all the previous 7 days files from NPEx and compared them with the test result report to ensure that all results had been uploaded correctly. If any issues were identified these were summarised and reported back to the Data Team Lead enabling rapid corrective action.
