# Supplementary material for: Improving the efficiency and effectiveness of an industrial SARS-CoV-2 diagnostic facility
Source: Sci Rep. 2022 Feb 24;12:3114. doi: 10.1038/s41598-022-06873-6 (PMC8873195; doi:10.1038/s41598-022-06873-6)
Supplement: Supplementary file 2 — Supplementary Information 2. [file 41598_2022_6873_MOESM2_ESM.docx]

**Supplementary information**

**Referenced CCTC Standard Operating Procedures (SOPs)**

SOPs were continuously updated as required in response to process improvements, provision of additional information etc. The most relevant and useful version or versions have been chosen for publication here in abridged form, comprising the detailed Procedure sections and other transferrable information. Information uniquely applicable to CCTC has been removed for clarity.

| SOP number | Process | Page numbers in this document |
| --- | --- | --- |
| SOP CB 01 (Version 2) | Sample Receipt (Version prior to Heat Inactivation implementation) | 2-5 |
| SOP CB 01 (Version 3.3) | Sample Receipt (Version following Heat Inactivation implementation) | 6-10 |
| SOP CB 02  (Version 11) | Swab sample preparation, lysis and deactivation (Version covering with and without bulk Heat Inactivation) | 11-30 |
| SOP CB 03  (Version 4.1) | RNA extraction from sample plates using the Biomek i5/i7 | 31-44 |
| SOP CB 05 (Version 5.2) | PCR preparation of RT-PCR reagents and plate set-up using High Throughput Kit | 45-49 |
| SOP CB 06 (Version 4) | Preparation and addition of positive control to RT-PCR plate, RT-PCR running and data export | 60-70 |
| SOP CB 17  (Version 6) | Data Analysis | 71-77 |
| SOP CB 33  (Version 2) | Swab vial preparation for Direct to PCR assay | 78-89 |
| Sop CB 34  (Version 2) | Direct to PCR – Pre PCR set up | 90-106 |

# SOP CB 01: STATION 0 OROPHARYNGEAL AND NASOPHARYNGEAL SWAB SAMPLE RECEIPT (Version2)

# PURPOSE

The Cambridge COVID-19 Testing Centre (CCTC) based at the Anne McLaren Building is one of the National Testing Centres for COVID-19. The purpose of this facility is to test for the presence the SARS-CoV-2 virus. This procedure provides the method for receipt of the samples on site and the transport to the sample preparation laboratories.

# AUDIENCE

This SOP applies to all on site trained personnel who are responsible for assessing samples for COVID-19 screening.

# SCOPE

This document defines the required procedure for Station 0, incorporating the inspection of packaging integrity and assurance of traceability. Delivery of the package to the sample preparation labs, removal and disposal of the outer most layer of packaging.

# GLOSSARY


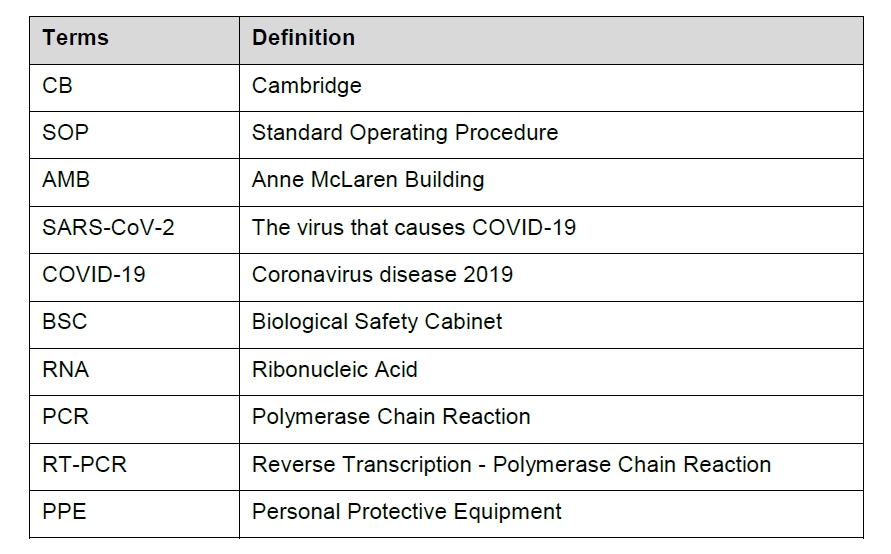


# SAFETY OVERVIEW

## 5.1 Hazards

| Distel (1:10) | H315 Causes skin irritation  H317 May cause an allergic skin reaction  H318 Causes serious eye damage  H351 Suspected of causing cancer  H373 May cause damage to organs through prolonged or repeated exposure |
| --- | --- |
| 70% Ethanol | H225 Highly flammable liquid and vapour  H319 Causes serious eye irritation |

## 5.2 Personal Protective Equipment

- Fully buttoned-up Howie-style laboratory coat in the general facility
- Extended-cuff nitrile gloves – double gloved when working within the Biological Safety Cabinet (BSC)
- Disposable sleeves (required if lab coat does not have elasticated cuffs, or if lab coat is non-disposable or not liquid-impermeable)
- Laboratory safety glasses or over-glasses

# PROCEDURE

## 6.1 General considerations

### 6.1.1 Warnings and precautions

- Handle all specimens as if infectious
- Refer to task-based risk assessment for handling infectious specimens
- Discard all gloves, pipette tips, pipettes, vials, test tubes, or other disposable items into appropriately labelled biohazard boxes or autoclave bags

6.1.2 Preventing Contamination

- Change gloves whenever contamination is suspected and when changing workstation
- Reagents and equipment should not be moved from the goods in/support side to the lab side. Should a case arise where a reagent or piece of equipment needs to be moved to goods in/support side, it must first be decontaminated with Distel (1:10) and wiped down with 70% ethanol

### 6.1.3 Exposure control plan

In case of sample spillage or any other risk of exposure to SARS-CoV-2 arising, the following measures need to be taken

#### 6.1.3.1 Spillage outside the BSC

- Immediately leave the room and discard any PPE that may have been in contact with contaminated sample as a hazardous waste.
- Contact the Shift/Lab Lead who will enact the spill procedure as outlined in SOP CB 09
- Shift Lead will inform staff when it is safe to continue working

##### 6.1.3.2 Receipt of samples with wet / presence of moisture on outer (UN3373 box) and / or tertiary containment.

(Note a suspected spill should be dealt with as above in Section 6.1.3.1. This section describes the action to take when a spill is not suspected, but the container outer is wet, for example from rain or from previous cleaning that has not been completely dried).

- Immediately close the outer UN3373 box.
- Treat the outside of the box as specified by SOP CB 09: Spray with Distel (1:10) and leave for 30 mins. Subsequently wipe clean with 70% ethanol.
- Pass the UN3373 box into the AMB Level 2 airlock for the Station 1 sample prep team to unbox inside a BSC.
- Proceed with 6.1.3.3.

##### 6.1.3.3 Contamination of PPE

- Immediately encapsulate all potentially contaminated PPE in an autoclave bag which should be double bagged, sealed with autoclave tape and sent to the autoclave.
- Wash hands/exposed skin according to best practice

## 6.2 Procedure Schematics

### 6.2.1 Room Schematics

Typical layout of a Sample Preparation laboratory showing the input point for sample drop off


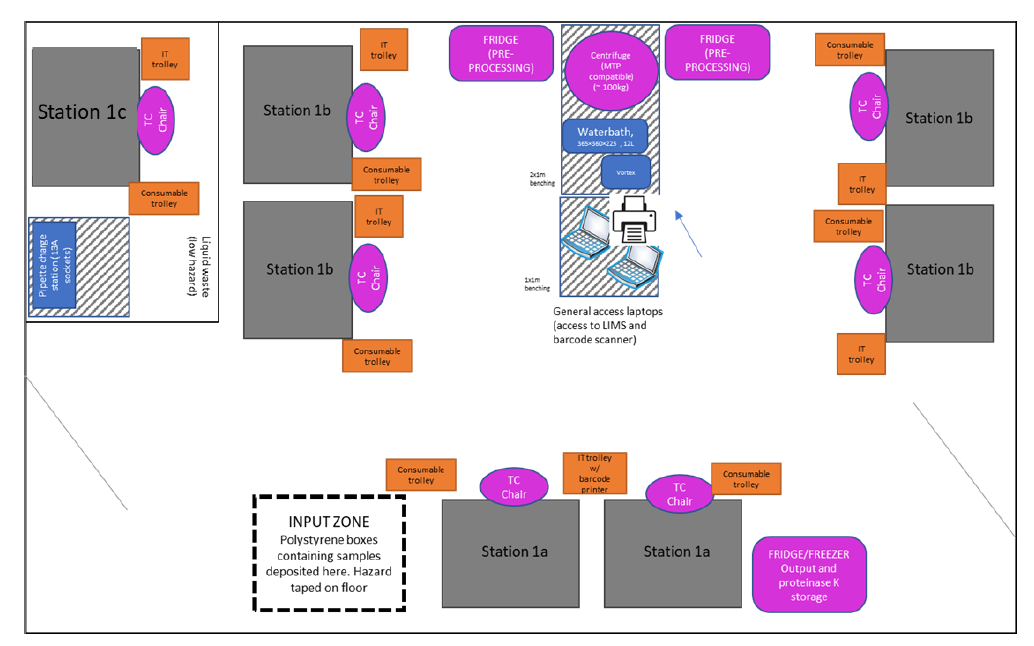


## 6.3 Materials and Reagents

| Reagent / Labware | Vendor and article # | Comment |
| --- | --- | --- |
| Distel (1:10) | VWR | Provided by Avantor |
| 70% Ethanol | VWR | Provided by Avantor |

| Equipment | Comment |
| --- | --- |
| Safety Knife |  |
| Skirted Trolley | For safe transport of boxes |

## 6.4 General guidelines

- Perform all steps at room temperature (20-25oC), unless otherwise noted.
- Perform all steps wearing appropriate PPE

## 6.5 Procedure

### 6.5.1 Sample receipt: Goods In

1. UN3373 boxes will arrive in goods in. This can consist of either MedDX containers or home test kits in carboard containers.
2. On receipt of the delivery, details of the delivery are recorded in Microsoft Teams tracker. If boxes are received in MedDX containers from Regional Test Sites, the ID of the MedDX containers is recorded and logged on University owned SharePoint site
3. Open UN3373 box
4. Inspect the UN3373 box in which samples have been provided for damage or signs of leakage. If signs of leakage are visible, refer to SOP CB 09
5. Visually check the tertiary containment bag (if present) for signs of leakage. If no signs of leak are present, transfer the contents to a **SAMPLE RECEIPT** box (clear box)
6. When home kits are received they are unpacked down to the sample bag (secondary containment) and placed in the sample receipt box. To support increasing sample numbers, the carboard tertiary containers may be passed through to sample prep.
7. Transport the Sample Receipt Box to the AMB level 2 airlock.
8. Return the UN3373 box to the courier

## 6.6 Waste Management

- All items of PPE that require disposal should be placed in the autoclave bags provided, sealed and sent for autoclaving as required

# RESPONSIBILITIES

- It is the responsibility of all personnel handling samples to follow this procedure.
- It is the responsibility of personnel performing this procedure to ensure all equipment, chemicals, reagents, and solutions are properly labelled and that proper documentation is maintained.
- It is the responsibility of the laboratory manager to ensure that this procedure is followed and that it is updated as necessary.

End of SOP CB 01 Version2

# SOP CB 01: STATION 0 OROPHARYNGEAL AND NASOPHARYNGEAL SWAB SAMPLE RECEIPT (Version 3.3)

# PURPOSE

The Cambridge COVID-19 Testing Centre (CCTC) based at the Anne McLaren Building is one of the National Testing Centres for COVID-19. The purpose of this facility is to test for the presence the SARS-CoV-2 virus. This procedure provides the method for receipt of the samples on site and the transport to the sample preparation laboratories.

# AUDIENCE

This SOP applies to all on site trained personnel who are responsible for assessing samples for COVID-19 screening.

# SCOPE

This document defines the required procedure for Station 0, incorporating the inspection of packaging integrity and assurance of traceability. Delivery of the package to the sample preparation labs, removal and disposal of the outer most layer of packaging.

# GLOSSARY


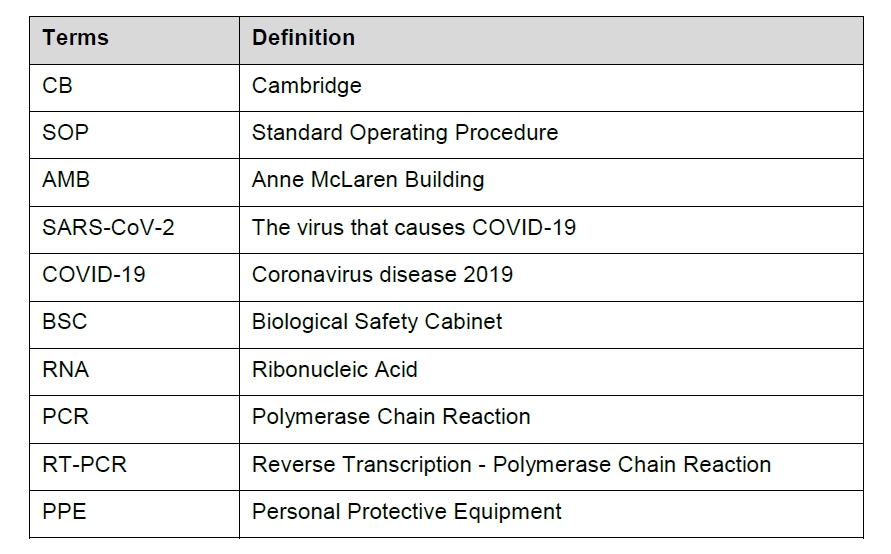


# SAFETY OVERVIEW

## 5.1 Hazards

| Distel (1:10) | H315 Causes skin irritation  H317 May cause an allergic skin reaction  H318 Causes serious eye damage  H351 Suspected of causing cancer  H373 May cause damage to organs through prolonged or repeated exposure |
| --- | --- |
| 70% Ethanol | H225 Highly flammable liquid and vapour  H319 Causes serious eye irritation |

## 5.2 Personal Protective Equipment

- Fully buttoned-up Howie-style laboratory coat in the general facility
- Extended-cuff nitrile gloves – double gloved when working within the Biological Safety Cabinet (BSC)
- Disposable sleeves (required if lab coat does not have elasticated cuffs, or if lab coat is non-disposable or not liquid-impermeable)
- Laboratory safety glasses or over-glasses

# PROCEDURE

## 6.1 General considerations

### 6.1.1 Warnings and precautions

- Handle all specimens as if infectious
- Refer to task-based risk assessment for handling infectious specimens
- Discard all gloves, pipette tips, pipettes, vials, test tubes, or other disposable items into appropriately labelled biohazard boxes or autoclave bags

6.1.2 Preventing Contamination

- Change gloves whenever contamination is suspected and when changing workstation
- Reagents and equipment should not be moved from the goods in/support side to the lab side. Should a case arise where a reagent or piece of equipment needs to be moved to goods in/support side, it must first be decontaminated with Distel (1:10) and wiped down with 70% ethanol

### 6.1.3 Exposure control plan

In case of sample spillage or any other risk of exposure to SARS-CoV-2 arising, the following measures need to be taken

#### 6.1.3.1 Spillage outside the BSC

- Immediately leave the room and discard any PPE that may have been in contact with contaminated sample as a hazardous waste.
- Contact the Shift/Lab Lead who will enact the spill procedure as outlined in SOP CB 09
- Shift Lead will inform staff when it is safe to continue working

##### 6.1.3.2 Receipt of samples with wet / presence of moisture on outer (UN3373 box) and / or tertiary containment.

(Note a suspected spill should be dealt with as above in Section 6.1.3.1. This section describes the action to take when a spill is not suspected, but the container outer is wet, for example from rain or from previous cleaning that has not been completely dried).

- Immediately close the outer UN3373 box.
- Treat the outside of the box as specified by SOP CB 09: Spray with Distel (1:10) and leave for 30 mins. Subsequently wipe clean with 70% ethanol.
- Pass the UN3373 box into the AMB Level 2 airlock for the Station 1 sample prep team to unbox inside a BSC.
- Proceed with 6.1.3.3.

##### 6.1.3.3 Contamination of PPE

- Immediately encapsulate all potentially contaminated PPE in an autoclave bag which should be double bagged, sealed with autoclave tape and sent to the autoclave.
- Wash hands/exposed skin according to best practice

## 6.2 Procedure Schematics

### 6.2.1 Room Schematics

Sample Receipt Cabin (Station 0)
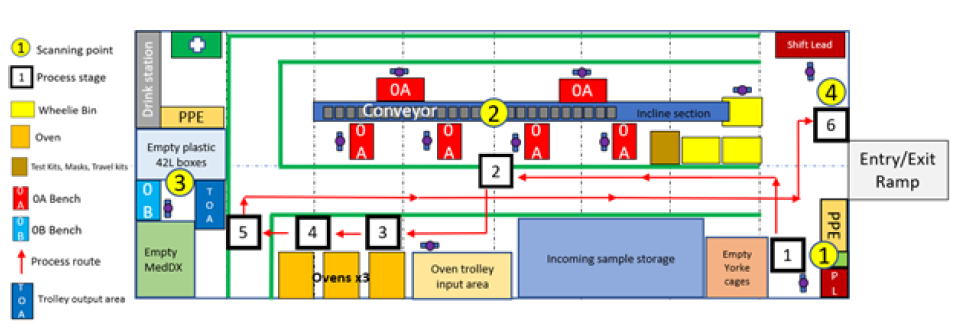


## 6.3 Materials and Reagents

| Reagent / Labware | Vendor and article # | Comment |
| --- | --- | --- |
| Distel (1:10) | VWR | Provided by Avantor |
| 70% Ethanol | VWR | Provided by Avantor |

| Equipment | Comment |
| --- | --- |
| Safety Knife |  |
| Skirted Trolley | For safe transport of boxes |

## 6.4 General guidelines

- Perform all steps at room temperature (20-25°C), unless otherwise noted.
- Perform all steps wearing appropriate PPE

## 6.5 Procedure

**6.5.1 Sample receipt: Goods In**

1. UN3373 boxes will arrive in goods in. This can consist of either MedDX containers or home test kits in carboard containers.

2. On receipt of the delivery, details of the delivery are recorded in Microsoft Teams tracker. If boxes are received in MedDX containers from Regional Test Sites, the ID of the MedDX containers is recorded and logged on University owned SharePoint site

3. Open UN3373 box

4. Inspect the UN3373 box in which samples have been provided for damage or signs of leakage. If signs of leakage are visible, refer to SOP CB 09.

5. Unpack the samples and visually check the secondary containment sample bags for signs of leakage. If a leak is found within the outer containment, take advice from the Sample Prep Scientist operating the oven on whether the sample should be heat-inactivated. Preferably all samples should be heat-inactivated whether they are fit for processing or will be disposed of.

6. If samples have non-standard packaging or contents, consult the Sample Prep Scientist operating the oven for guidance on whether heat inactivation should be performed. Examples of where heat inactivation should NOT be performed include instances where there is no sample bag, or when a sample bag contains paperwork such as the testing information booklet.

7. Load samples into oven trays containing dividers with vial caps uppermost (place sample bags vertically on their long edge, with the long edge aligned to the short side of the tray). Include 25 samples in a tray. If fewer than 25 samples are available, use ‘dummy’ samples to reach a total of 25. To ensure 25 samples are added, count 25 samples in and then recount once the tray is full. Place 2 samples per divider for 12 of the dividers and 1 in the remaining divider.

**NOTE 1: Only sample vials suitable for heat inactivation should be used.**

**NOTE 2:** Oven trays without dividers are also available but should only be used if divider trays are unavailable. An oven must only be loaded with one type of tray.

8. Insert full trays of 25 samples into a Trolley Tray Rack. Wheel the full Trolley to the dedicated OVEN INPUT area adjacent to the oven. Apply the foot brake on the trolley.

9. The samples are heat inactivated in the oven following the process described in SOP CB 35: STATION 0: Heat inactivation of samples in packaging at Sample Receipt for processing of samples through the oven and control of heat inactivation. After heat inactivation, trolleys will be placed in the OVEN OUTPUT area.

10. If there is any problem with the Heat Inactivation process, in accordance with SOP CB 35, samples will be unloaded from the oven and placed in a dedicated VIABLE SAMPLE box (clear box) by the oven operator, keeping the vials lid uppermost, and will be clearly labelled as such prior to transfer to the laboratory. The samples will be transferred separately from heat inactivated samples (see also point 12).

11. On successful completion of the oven cycle, transfer heat inactivated samples from the OVEN OUTPUT area to a SAMPLE RECEIPT box (clear box) - Keeping the vials lid uppermost

12. Transport the Sample Receipt Boxes to the level 4 Sample Prep lab. Non heat inactivated VIABLE SAMPLE boxes must be handed directly to a lab lead for separation and special processing within Containment level 2.

13. Return the UN3373 box to the courier

## 6.6 Waste Management

- All items of PPE that require disposal should be placed in the autoclave bags provided, sealed and sent for autoclaving as required

# RESPONSIBILITIES

- It is the responsibility of all personnel handling samples to follow this procedure.
- It is the responsibility of personnel performing this procedure to ensure all equipment, chemicals, reagents, and solutions are properly labelled and that proper documentation is maintained.
- It is the responsibility of the laboratory manager to ensure that this procedure is followed and that it is updated as necessary.

End of SOP CB 01 Version3.3

**SOP CB 02: Oropharyngeal and nasopharyngeal swab sample manual preparation and viral deactivation prior to RNA extraction (Version 11)**

# PURPOSE

The Cambridge COVID-19 Testing Centre (CCTC) based at the Anne McLaren Building is one of the National Testing Centres for COVID-19. The purpose of this facility is to test for the presence the SARS-CoV-2 virus. This procedure provides the method for preparing and deactivating human swab samples prior to RNA extraction using the Biomek i5/i7 in a safe and consistent manner.

# AUDIENCE

This SOP applies to all on site trained personnel who are responsible for assessing samples for COVID-19 screening.

# SCOPE

This document defines the required procedure for Station 1, incorporating the sorting, inactivation and lysis of viral particles for RNA extraction from swab samples with the purpose of detecting the presence of SARS-CoV-2 viral RNA using the COVID-19 RT qPCR assay kit from PrimerDesign LTD.

# GLOSSARY


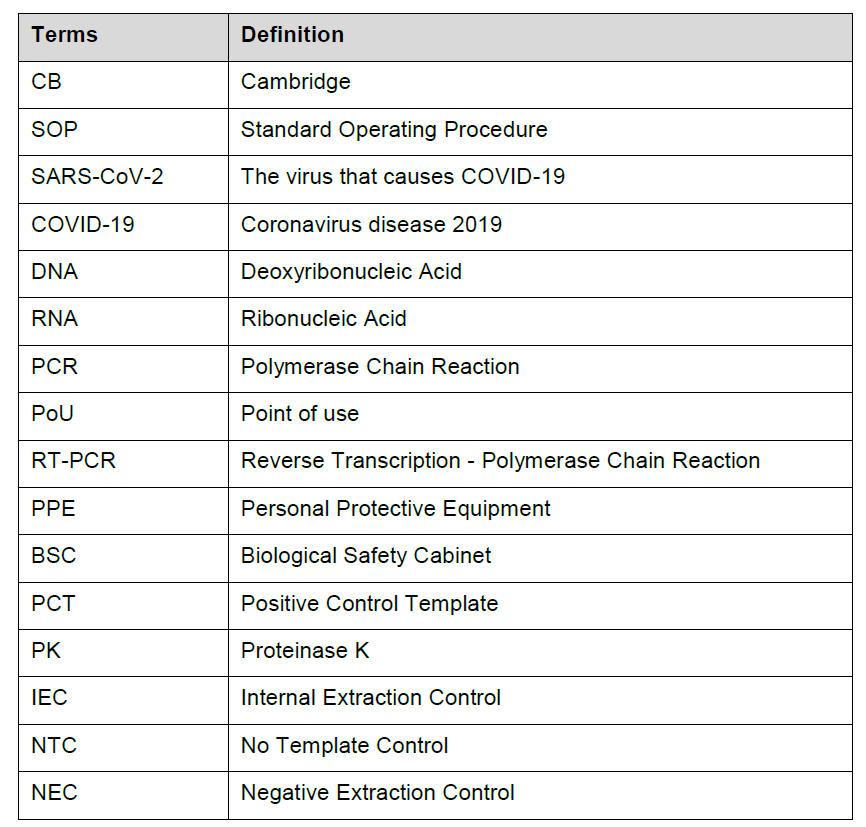


# SAFETY OVERVIEW

## 5.1 Hazards


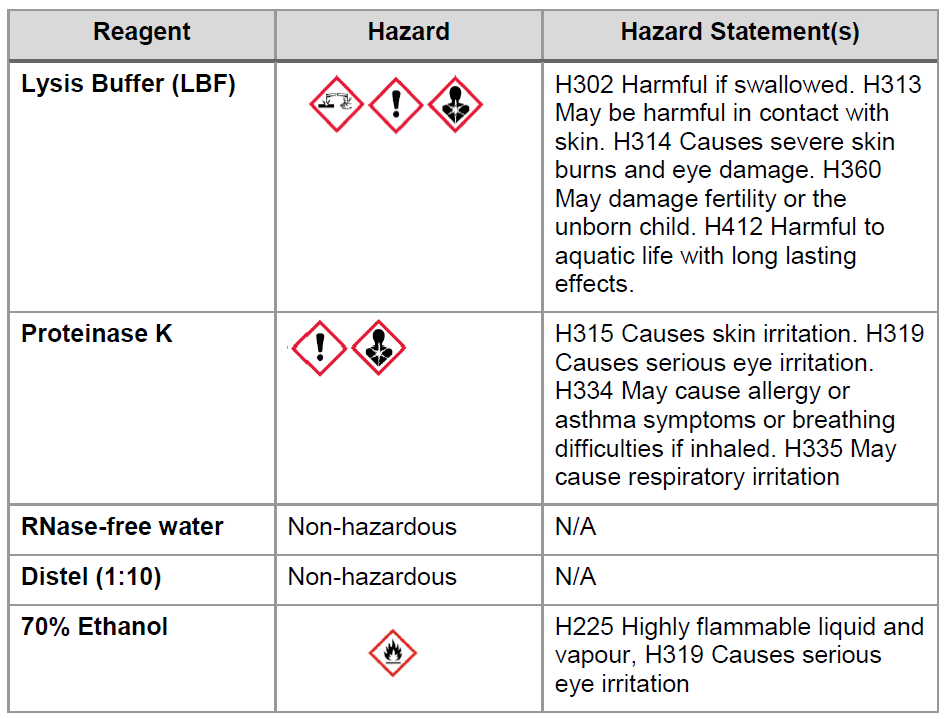


## 5.2 Personal Protective Equipment

- Fully buttoned-up Howie-style laboratory coat in the general facility
- Extended-cuff nitrile gloves – double gloved when working with samples
- Disposable sleeves for non-heat inactivated samples (required if lab coat does not have elasticated cuffs, or if lab coat is non-disposable or not liquid-impermeable)
- Laboratory safety glasses or over-glasses (Where vision is impeded when used with masks and the operator is positioned at a BSC and protected, they may temporarily work without glasses)
- All PPE should be removed before exiting the sample prep rooms

# PROCEDURE

## 6.1 General considerations

### 6.1.1 Warnings and precautions

- Handle all specimens as if infectious
- **Refer to task-based risk assessment for handling infectious specimens**
- Discard all gloves, pipette tips, pipettes, vials, test tubes, or other disposable items into appropriately labelled biohazard boxes or autoclave bags.
- Use separated working areas for specimen preparation, reaction set up and amplification, with separate supplies and equipment
- **NOTE: Use of Virkon is not permitted in the Sample Lysis laboratory due to the risk of accidental mixing with lysis buffer containing guanidine thiocyanate, which may result in the release of cyanide gas.**

### 6.1.2 Preventing Contamination

- Change gloves whenever contamination is suspected and, changing outer glove when changing workstation (e.g. station 1a to station 1b)
- BSC, workbenches, and pipettes should be cleaned and decontaminated with 1:10 Distel (v:v), and 70% (v:v) ethanol to minimize risk of contamination. Spray and wipe the surfaces with Distel followed with 70% ethanol.
- Reagents and equipment should not be moved from a dirty area to a clean area. Should a case arise where a reagent or piece of equipment needs to be moved backwards (dirty to clean), or contamination is suspected, it must first be decontaminated with Distel and wiped down with 70% ethanol.
- Use dedicated equipment (pipettes, boxes, racks etc) in SP1, and do not use in labs used for heat inactivated samples
- Do not use Virkon, see above.

### 6.1.3 Preventing DNase/RNAse contamination

- Use DNase/RNase free disposable plasticware and pipettes reserved for DNA/RNA work to prevent cross-contamination with DNases/RNases from shared equipment
- Use DNase/RNAse free filter tips throughout procedure to prevent aerosol and liquid contamination
- Wipe surfaces of workbenches and BSC with RNAse Away followed by 70% ethanol

### 6.1.4 Exposure control plan

In case of sample spillage or any other risk of exposure to SARS-CoV-2 arising, the following measures need to be taken. Please refer to SOP CB 09 for details pertaining to spillages

#### 6.1.4.1 Spillage on bench or in BSC for **heat inactivated** samples

- Mop up the liquid using absorbent pads/paper and discard in the biohazard bin on the workbench
- Treat area of spillage with Distel (1:10), RNase away and 70% ethanol
- Remove top layer of gloves and dispose of in biohazardous waste.
- All spills should be reported to the lab lead

#### 6.1.4.2 Spillage outside the BSC for non-heat inactivated samples

- All scientists discard any PPE that may have been in contact with contaminated sample as a hazardous waste and leave the room.
- Immediately contact team leader or shift leader and await further instruction, scientists are to remain in the vicinity and not disperse into other rooms.
- The team leader/shift leader will enact the “Uncontained Spill Procedure” as outlined in SOP CB 09.

#### 6.1.4.3 Spillage inside the BSC for non-heat inactivated samples

- Mop up the liquid using absorbent pads/paper and discard in the biohazard bin in the BSC
- Treat area of spillage with Distel (1:10) for 30 min
- Remove top layer of gloves and sleeves inside the BSC
- After 30 min follow with a wipe with 70% ethanol. Discard waste tissue / paper towel in the biohazardous bin in the biosafety cabinet
- Remove top layer of gloves and sleeves inside the BSC
- All spills should be reported to the lab lead

#### 6.1.4.4 Contamination of PPE with any biological sample

- Immediately encapsulate all potentially contaminated PPE
- Wash hands/exposed skin according to best practice

## 6.2 Procedure Schematics


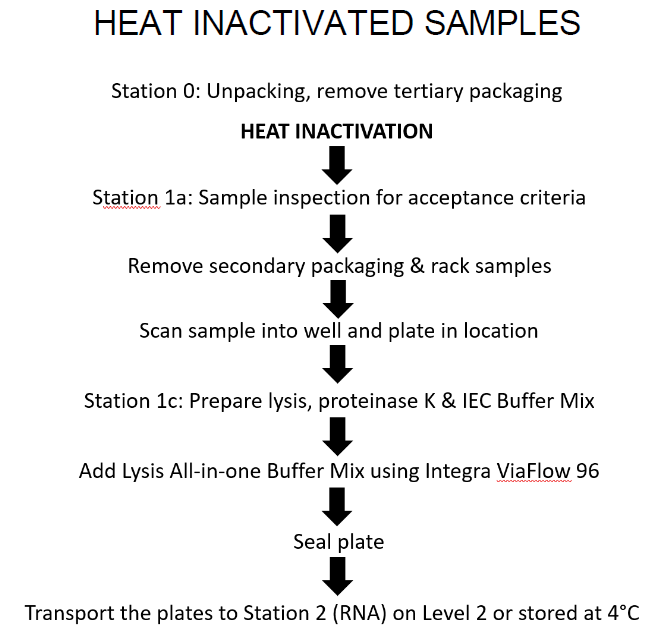


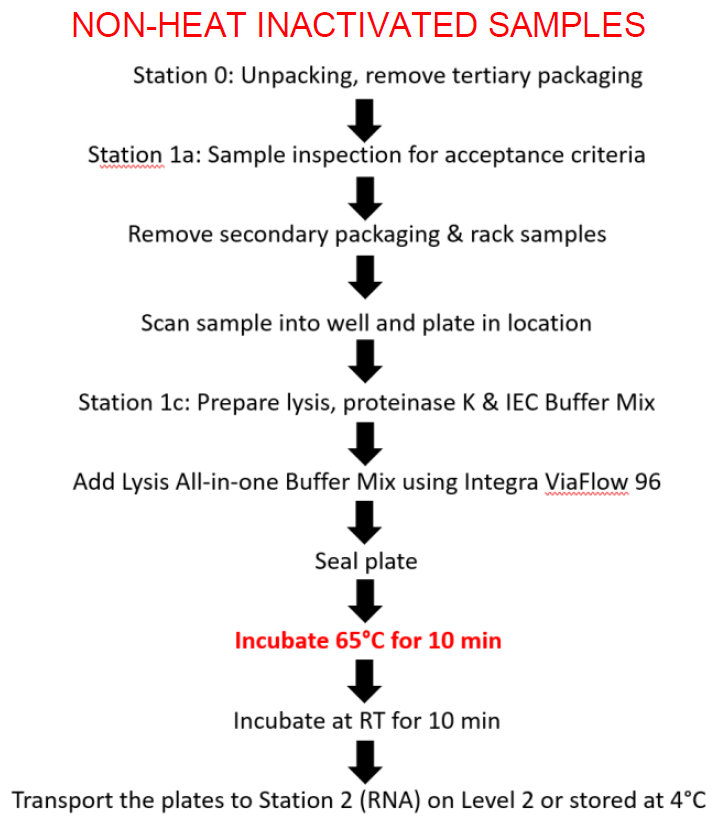


## 6.3 Workstation Schematic

For reference, a typical layout of a Station 1 Sample Preparation Laboratory.


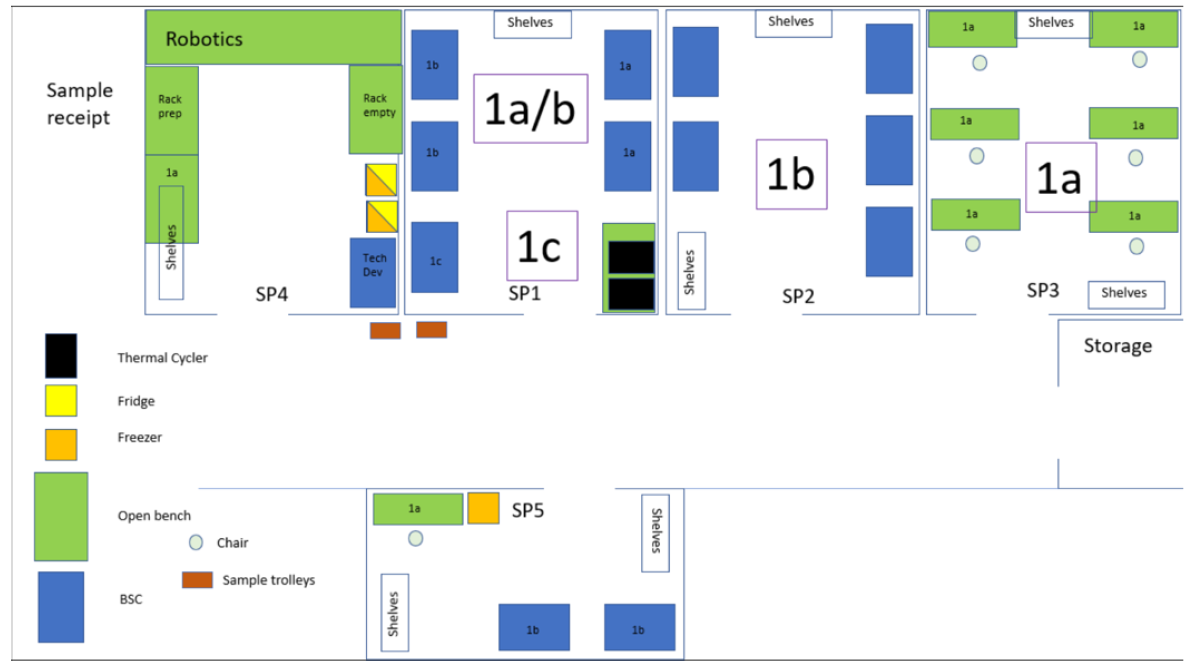


## 6.4 Materials and Reagents


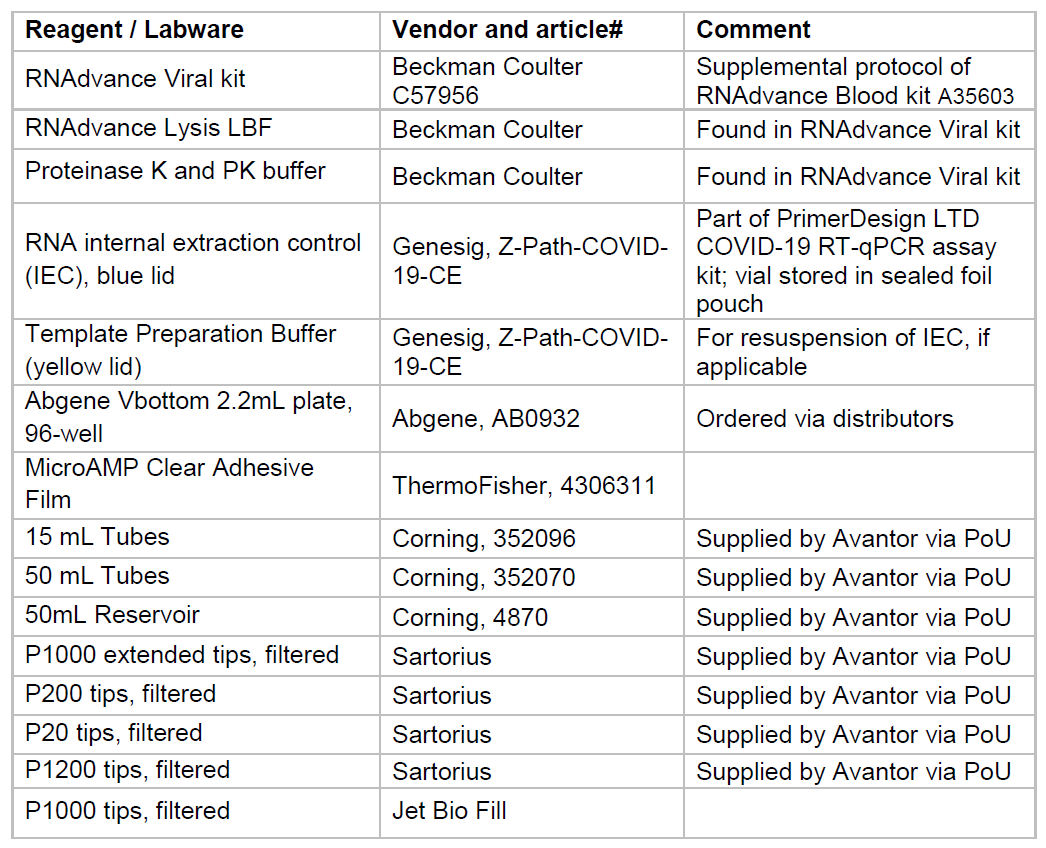


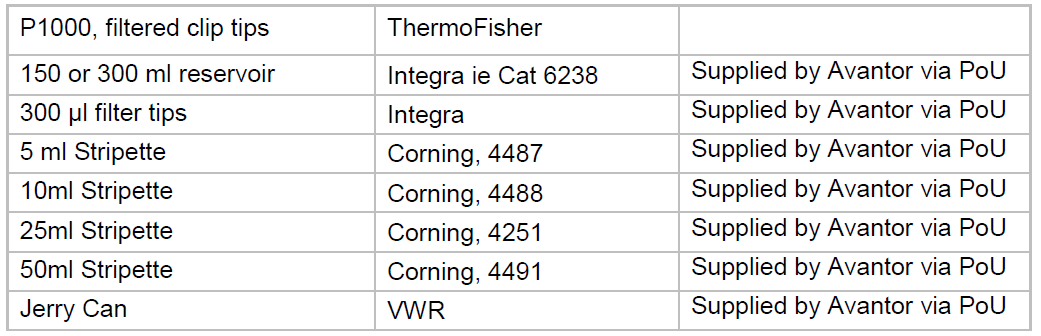


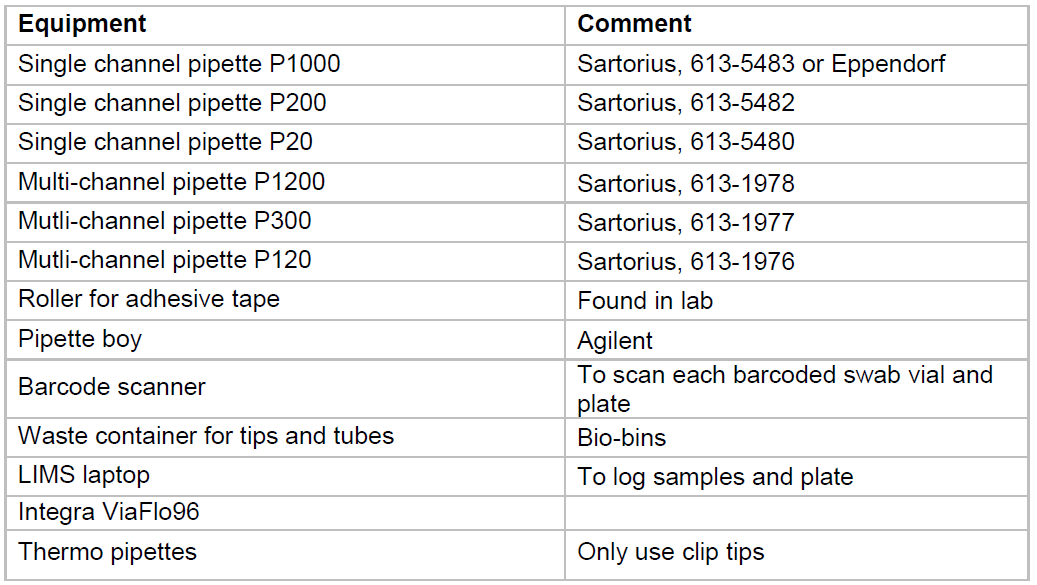


## 6.5 General guidelines

- Perform all steps at room temperature (20-25°C), unless otherwise noted.
- Perform all steps wearing appropriate PPE
- Avoid creating bubbles during mixing and aliquoting

## 6.6 Procedure

- Perform all actions with non-heat inactivated samples in a Class 2 BSC within a CL2 laboratory
- To avoid confusion, all work with non-heat inactivated samples is carried out in a dedicated room
- Refer to plate map for guidance
- All waste including tips should be placed in Bio-bins, sealed and double contained in an autoclave bag if necessary, before leaving the BSC or workbench. Refer to Risk Assessments and Waste management (Section 6.6.6 for guidance.
- The below procedures for stations 1b and 1c are for the use of the ViaFlo96. Should this instrument not be available, operators should proceed to 10.3 to follow the manual process.

### 6.6.1 Sample preparation: Station 1a for non-heat inactivated samples

*Note: Samples that have* ***not been successfully heat treated in the oven*** *will be clearly labelled and processed in a BSC. All samples that come out of this hood will also be clearly labelled for a second heat inactivation (Section 6.6.4.27)*

1. Ensure the BSC is compliant has been tested in the last 12 months and within operational safe working limits. Airflow information on the front of the cabinet should not be in red and no alarms should be sounding. If hood is non-compliant do not proceed and report to the shift lead.

2. Wipe down inside of biological safety cabinet with Distel (1:10) followed by 70% ethanol, discarding wipes in biohazard waste container in the BSC.

3. Obtain the delivered container of samples from the input zone. Remove sample bags from the container (tertiary packaging), placing them in the BSC.

4. Inspect the vials for leaks and ensure they meet the acceptance criteria.

a. The volume of transport media should be sufficient*

b. The sample tube should be intact

c. The sample should contain a single swab (with the exception of samples from University of Cambridge which will contain multiple swabs)

d. A barcode should be present on the swab tube

e. The sample should not contain visible contamination with mould

*Note: Clarification on barcode acceptance can be found in the appendix and has been defined by Deloitte.*

**Volume should be greater than 200 μl but not fill the sample tube as this is an error.*

5. If samples fail inspection, the operator should ensure the sample is double contained. An additional bag should be used if needed. Then mark the bag with a number corresponding to the spoil reason (see Section 10 Appendix). Place the sample in a plastic container labelled ‘Void’ whilst still in the BSC to triple contain the samples. Set aside the box and inform lab or shift lead for recording in LIMfinity and disposing as per SOP CB 10.

6. If the sample is accepted, remove the vial from the secondary packaging, discard packaging in an autoclave bag placed within the BSC.

7. Place the vial in the racking contained in a Tupperware container lined with absorbent mat for secondary containment.

8. Once a rack is full, secure the lid, wipe down the external surfaces with Distel (1:10) and 70% ethanol. The Tupperware is then taken to Station 1b and placed in the pre-processing input area (fridge/shelves).

9. At end of working period, repeat 6.6.1.2 to clean the BSC.

### 6.6.2 Sample preparation: Station 1a for heat inactivated samples

1. Wipe down the work area with Distel (1:10) followed by 70% ethanol, discarding wipes in biohazard waste container next to the workbench.

2. Obtain the delivered container of samples from the input zone. Remove sample bags from the container (tertiary packaging), placing them on the work area.

3. Inspect the vials to ensure they meet the acceptance criteria:

a. The volume of transport media should be sufficient*

b. The sample tube should be intact

c. The sample should contain a single swab (unless known to be part of a pooled screening approach)

d. A barcode should be present on the swab tube

e. The sample should not contain visible contamination with mould

*Note: Clarification on barcode acceptance can be found in the appendix and has been defined by Deloitte.*

**Volume should be greater than 200 μl but not fill the sample tube as this is an error.*

4. Inspect the sample bags for moisture and leaks:

a. If the outer bag is damp from the heat inactivation, there is no change to procedure, continue to process samples as normal

b. If moisture is seen between the inner and outer bag, carefully open outer bag and remove inner bag to check if moisture is also visible within the inner bag. If no moisture is visible, process as normal. If moisture is present within inner bag follow point c.

c. If moisture is seen within the inner bag: Handle the sample vial carefully, avoiding contact with liquid, wipe the tube with a Distel (1:10) tissue and rack as normal. Dispose of all used tissue in autoclave waste bag after each vial is wiped. Change outer gloves.

*d. Note: Use the void process for significant leaks where outer vial contamination with liquid cannot be effectively wiped*

5. If samples fail inspection, the operator should ensure the sample is double contained. An additional bag should be used if needed. Then mark the bag with a number corresponding to the spoil reason (see Section 10 Appendix). Place the sample in a plastic container labelled ‘Void’ whilst still working at the workbench. Set aside the box and inform lab or shift lead for recording in LIMfinity and disposing as per SOP CB 10.

6. If the sample is accepted, remove the vial from the secondary packaging, discard packaging in an autoclave bag placed next to the workbench.

7. Place the vial in the racking contained in a Tupperware container lined with absorbent mat for secondary containment.

8. Once a rack is full, secure the lid and take to Station 1b and place in the pre-processing input area (fridge/shelves).

9. At end of working period, repeat 6.6.2.1 to clean the workbench.

### 6.6.3 Sample preparation: Station 1b

*Note: This procedure is conducted by two operators. Operator One will conduct the sample work within the BSC. Operator Two will shadow, checking samples and reagents are added to the correct wells and operating the electronic LIMS system ensuring correct recording. Where the operator is not defined, this step may be completed by either.*

*Note: Take note of Tupperware containers coming out of 1a. If it states that the samples have not been heat inactivated then outer sleeves and doubles gloves should be worn by Operator 1 through the procedure, with all items that are removed from the hood being thoroughly cleaned with Distel and 70% ethanol. If samples have been heat inactivated, then Operator 1 will require double gloves but not outer sleeves and will only be required to wipe items out of the hood with Distel and 70% ethanol if they are suspected to be contaminated.*

1. Operator 1 will sign into the Viral Prep Tool App and with their credentials as they will be the one conducting the wet work in the BSC.

2. Ensure BSC is compliant and within operational safe working limits. All work to be conducted in the BSC unless otherwise stated.

3. Sequentially wipe down inside of BSC with Distel (1:10), RNAse Away and 70% ethanol. Clear up residue at each step with tissue / paper towel and discard in biohazard waste container in the hood.

4. Waste containers consisting of Bio-bins in an autoclave bag should be placed in the hood, ensuring the airflow is not obstructed, ready for sample and waste discarding during the process.

5. Remove a Qnostic Positive Control from the -20°C freezer in Sample Prep (aliquots of working stock will be stored at -20°C with bulk stock kept at -80°C). Allow to defrost at room temperature. Ensure a barcode is taken from the box when collecting the vial.

*Note: The Qnostic positive control is to be added to every sample prep plate when possible. Its position is to be randomly placed in the plate during steps 9-11 of Section 6.6.2*

6. Collect a Tupperware containing racked samples from the pre-processing input area (fridge or shelves) or station 1a.

7. Once Operator 1 is positioned in the hood with the correct PPE, Operator 2 is to pass an empty deep 96 well plate (AB0932) into the BSC and a barcode. Operator 1 will attach a barcode to the left-hand side of the plate. Lifting it to the glass, Operator 2 will scan the plate barcode into the top box on the app which is selected in blue.

*Note: if any problems should occur at the start and the app does not work, inform the lab lead and switch to the previous excel sheets as detailed in the appendix.*

8. Operator 1: Next using a P1000 Pipette, add 200μL nuclease free water to wells A1-C1 for the controls as shown in the plate layout. Paying particular care to C1 the negative extraction control (NEC).

9. Operator 1 will remove the first sample vial and hold to the glass for operator 2 to scan into the sample box highlighted in blue. Sample will be assigned a well location by the app. Operator 2 will call the well location.

10. Operator 1 will remove the lid over a Bio-bin, discarding it in the waste, and using a P1000 pipette with extended tips, transfer 200μl to the designated well. This will be verified by operator 2. The vial will be placed in the waste.

11. Repeat for the remaining sample vials, take column-wise from input rack, transfer to sample plate column-wise, scanning each vial in and discarding them in the Bio-bin / autoclave bag. One sample space should be used at random for the Qnostic positive control as stated in step 5 of section 6.6.2.

*Note: Should any problem occur with either the sample or pipetting of the sample, immediately alert the lab lead or designated super user of the sample prep tool app. Lab lead will follow instructions as stated in SOP CB 10. This is not to be conducted by the operators.*

*If barcodes do not scan correctly, manually enter the sample ID with verification from the other operator and hit enter. Inform lab lead of any issues.*

12. Operator 1: Seal plates with microamp clear adhesive seal.

13. Transfer of plates to 1C station:

a) For non-heat inactivated samples transfer the plate into two sample bags to double contain. Wipe with Distel (1:10) and 70% ethanol and remove from the hood and transfer to Station 1c in the non-heat inactivated input area. Inform the operator manning Station 1c to continue with Section 6.6.3.

b) *For heat inactivated samples the plate should be contained in a labelled transfer box to be taken to the 1c station in the heat inactivated input area, without the necessity to wipe the box with Distel and 70% ethanol.*

14. Operator 1: Discard any reservoirs or waste into the waste bin in the BSC avoiding any spillage as defined in 6.6.5 Waste Management. Clear working area and wipe down surface and pipettes with Distel (1:10) and 70% ethanol as required. Seal the waste bins taking particular note of the Bio-bins containing the remaining sample liquid, close the autoclave bag and pass to Operator 2 who will ensure bins are correctly sealed and aid placement into a second bag. Remove outer gloves and sleeves, if used, as bins are removed or place in a new Bio-bin in the BSC.

15. Operator 2: Navigate to tools in the top menu of the app. Record the lot numbers of the reagents used and the BSC used under ‘other’. Close tool when finished. Speak with the Operator of Station 1c for reagent details.

**6.6.4 Lysis All-In-One Reagent Addition: Station 1c using the ViaFlo96**

*Note: This procedure is conducted by two operators when making up the all-in-one Buffer. Operator One will make the buffer within the BSC. Operator Two will shadow, checking reagents are added correctly. Only one operator is required to run the ViaFlo96 and does not require witnessing. Where the operator is not defined, this step may be completed by either.*

*Note: If the ViaFlo96 fails QC or will not operate, the all in one lysis buffer can be added using a manual process. See 10.3 Appendix.*

*Note: One integra is dedicated to non-heat inactivated samples, with aligned ovens, input and output areas indicated by lab signage. The second integra is dedicated to heat inactivated samples only, aligned with labelled input and output areas*

1. Ensure BSC is compliant and within operational safe working limits. All work to be conducted in the BSC unless otherwise stated.

2. Sequentially wipe down inside of BSC with Distel (1:10), RNAse Away and 70% ethanol. Clear up residue at each step with tissue / paper towel and discard in biohazard waste container in the hood.

3. An autoclave bag should be placed in the hood, ensuring the airflow is not obstructed, ready for sample and waste discarding during the process.

4. At the start of a shift, the ViaFlo96 should be QC’d before use as directed below

a) Turn on the ViaFlo96 using the power switch behind the deck on the right-hand side

b) Read instructions on the pipette screen to home the device. Press ‘run’ when instructed to home the head position and repeat when instructed.

c) Add a reservoir to deck A and attach tips as directed below in section 6.6.3.11-16.

d) Following the following protocols, full details can be found in the appendix Section 10.

e) QC1: Fill the reservoir with 27 ml water. In custom programmes, run QC1. When run, leave for 30 seconds to ensure passes. Click run to complete the run.

f) QC2: Fill a new reservoir with 50 ml lysis buffer. In custom programmes, run QC2 and leave for 1 min. Click run to complete the run

g) QC3: Fill the reservoir with 50 ml water. In custom programmes, run QC3. Click run to complete the run

h) Unload the tips and dispose of the liquid waste in the jerry can.

*Note: If QCs fail, please repeat with fresh tips. Should it fail again, inform the lab lead or shift lead and take note of the batch number of the Integra tips.*

5. If opening a new RNAdvance Viral kit, resuspend Proteinase K by adding the appropriate volume of PK Buffer as defined by the manufacturer. 10 ml of PK buffer to 500 mg Proteinase K.

6. Remove the required number of Genesig Easy RNA Internal buffer extraction control (IEC) aliquots from the freezer to defrost.

*Note*: *Thawed and opened IEC aliquots must be disposed of at the end of a shift and once thawed, IEC must not be re-frozen. Defrosted unopened IEC aliquots may be handed over to the following shift for lysis buffer preparation.*

7. Obtain a 50 ml falcon tube or suitable RNAse/DNase free clean 100 ml bottle. Operator 3 will prepare the all in one lysis buffer using the following volumes. Operator 4 will verify the addition of the reagents.

- *Note: The ViaFlo96 requires a dead volume, providing an additional 5 ml in the reservoir is sufficient. The below calculations take that into account.*
- *Note: Once made up, the all in one lysis buffer must be used within 1 hr. Record the time the buffer mix was made up on the bottle.*


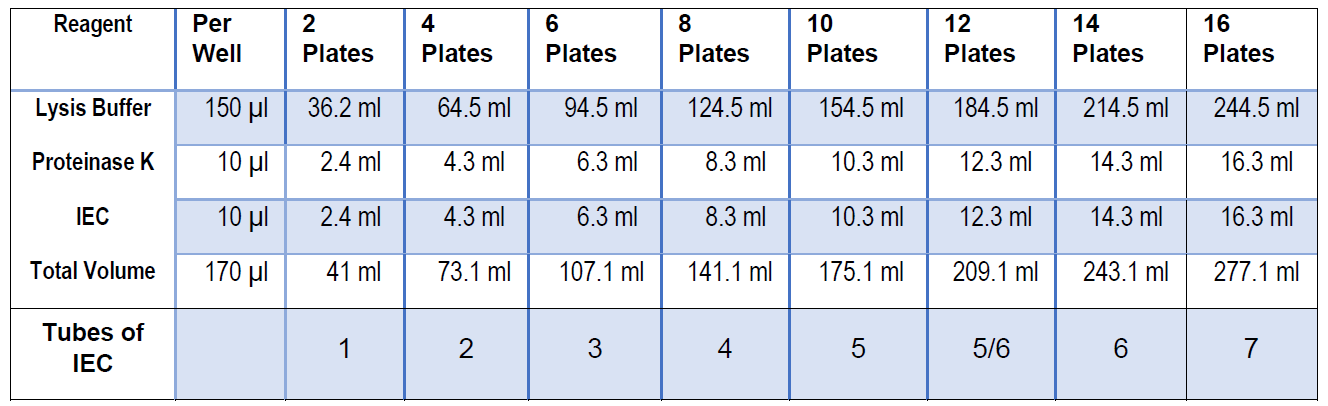


8. The number of tip boxes matched to the quantity of lysis buffer in the reservoir shall be identified and segregated for the run. Operator 2 will pass in 1 or 2 at a time with the respective plates.

- *Note: At the end of the run, it shall be confirmed that all the tip boxes have been used.*

9. Operator 3 will proceed with the following steps. Turn the ViaFlo96 using the power switch behind the deck on the right-hand side if it is not already on.

10. Read instructions on the pipette screen to home the device. Press ‘run’ when in structed to home the head position and repeat when instructed.

11. Place a new Integra reservoir into the Integra reservoir holder (deck A) as imaged below.


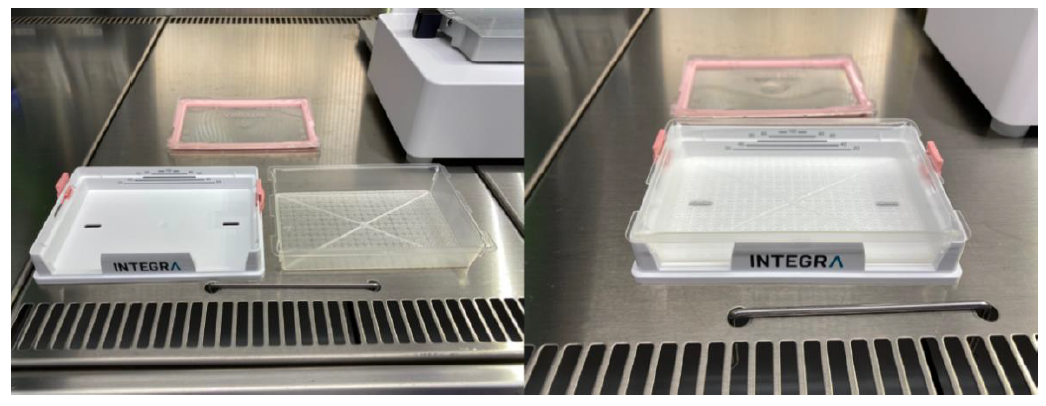


12. Carefully pour the Lysis all-in-one Buffer (maximum volume for 8 plates) into the reservoir. *If Lysis all-in-one Buffer is prepared for more than 8 plates, the maximum volume to be added to the reservoir (accurately, using a stripette) is for 8 plates and the rest held in a closed bottle,* ***labelling 'a' and 'b'*** *both on the bottles and on the lysis tracking spreadsheet. After 8 plates, the reservoir is replaced and a new reservoir loaded with the remaining buffer.*

- *Note: It is recommended to rest a clean plate seal on top of the reservoir (i.e. not physically adhere the seal to the reservoir). Carefully lift the seal just before triggering integra action and re-cover the reservoir as soon as integra tips have moved out of the way.*

13. On deck B, place a clean box of Integra 300 μl tips (green box) in the orientation shown below


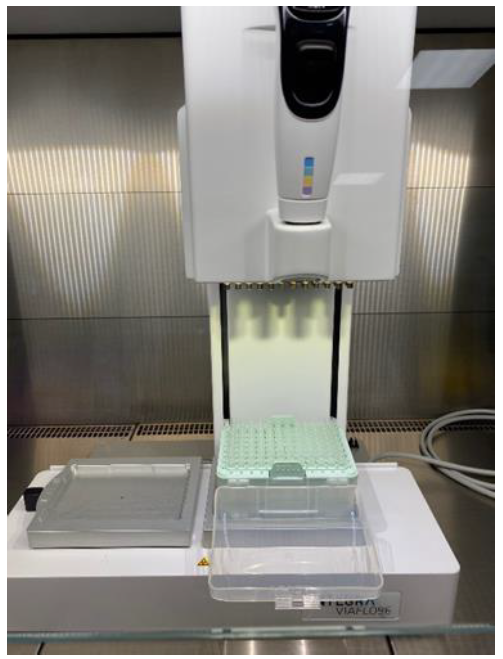


14. Ensure the pipette is in main menu

15. Hold the pipette on the instrument and move it to the right, as far as it goes. Bring the head down onto the tips until the blue button lights up on top. Hold the head in place and press the blue button with the other hand.

16. Tips will be loaded onto the head. Once done so, raise the pipette up and place the tip box to the side for use later.

17. Unseal a single viral plate at a time by carefully removing the plate seal to the side (NOT in front) of Integra.

18. Place the unsealed viral plate on the right (deck B) as pictured below.


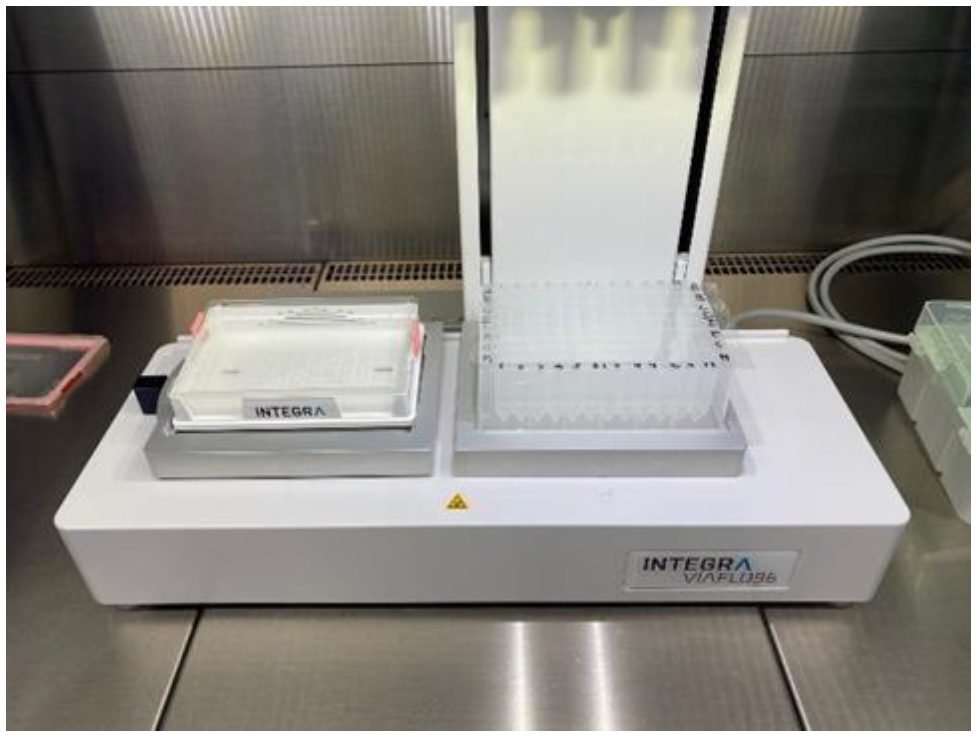


19. Take hold of the pipette again and using the wheel, scroll to the ‘custom programmes’, click the middle of the wheel to select

20. Scroll down to ‘Lysis Mix Auto’ and click to select.

21. Do not hold the pipette but click ‘Run’ on the pipette below the menu wheel. The protocol will run, taking 170 μl up, transferring it to the plate and mixing 10 times.

22. As soon as the pipette has finished and lifted, remove the plate and seal plates with microamp clear adhesive seal and *secure with a rubber roller (or similar method) to ensure adherence of the seal to all well walls.*

23. Immediately place the tip box on deck B below the tips.

24. Click the back arrow on the pipette twice to return to the main menu before the next step.

25. Take hold of the pipette again and make sure it is as far to the right as it goes. Slowly bring the pipette down until the tips are partway into the rack. Click ‘eject’ twice. Tips will release into the box. Discard tips in the waste bin in hood.

26. Transfer the plate containing samples out of 1c hood

a) If the samples have been heat inactivated prior to 1c stage then plate can be removed without being wiped or placed in plastic bags for double containment, unless contamination is suspected.

b) If samples have not been heat inactivated, double contain sealed plate within two sealable bags and wipe with Distel (1:10) and 70% ethanol before removing from the hood.

27. If the plate contains samples that have been heat inactivated at sample receipt, place them into dedicated Tupperware containers and incubate at room temperature for 20 min. A maximum of 2 plates may be placed per container. The incubation time can include transport time the Station 2 for RNA extraction.

28. For non-heat inactivated samples, place the plate in the incubator set to 65°C for 10 minutes. Then place at room temp for 10 min. Remove the bags from the plate and place the plate in a dedicated Tupperware container lined with absorbent mats. A maximum of 2 plates may be placed per container.

29. Before sending plates to Station 2 (RNA), they should be visually checked for the correct well volumes and for the presence of a barcode (on the correct side).

30. Take the box to the sample prep goods in area and locate the blue trolley. Carefully place the box on the top shelf of the trolley, taking care to avoid overcrowding when there are multiple boxes

*Note: Avantor will come and collect the trolley at regular intervals c. 20 mins. If the frequency needs adjusting, to ensure efficient movement of plates to RNA, contact Avantor.*

**Plates will be moved as stated in SOP CB 27.**

31. Inform RNA by entering the plate number and time of lysis mix addition into the plate timings Excel sheet.

- *Note: Plates can remain at 4°C overnight, however by the end of the working day, all plates must be pushed to Station 2. Station 2 will record time and date of arrival if a hold is required overnight.*

32. Repeat from step 13 to run further plates until the buffer is used up.

- *Note: At times of peak throughput consider having a third person (could be lab lead) assist at 1C, to take responsibility for moving plates through heat inactivation, cooling and delivery of plates to transport trolley, allowing the second person to witness/supervise the activities of the Integra operator more closely.*

33. Turn off instrument

34. Discard any dry reservoirs or waste into the waste bin in the BSC avoiding any spillage as defined in 6.6.5 Waste Management. Any excess liquid waste from the lysis can be placed in a 50 ml falcon tube and discarded in the Bio-bin. Larger volumes should be placed in a jerry can. Clear working area and wipe down surface and pipettes with Distel (1:10) and 70% ethanol as required. Seal the waste bins, close the autoclave bag and pass to another operator for placement into a second bag. Remove outer gloves and bin in a new Bio-bin in the BSC.

### 6.6.5 Plate Layout


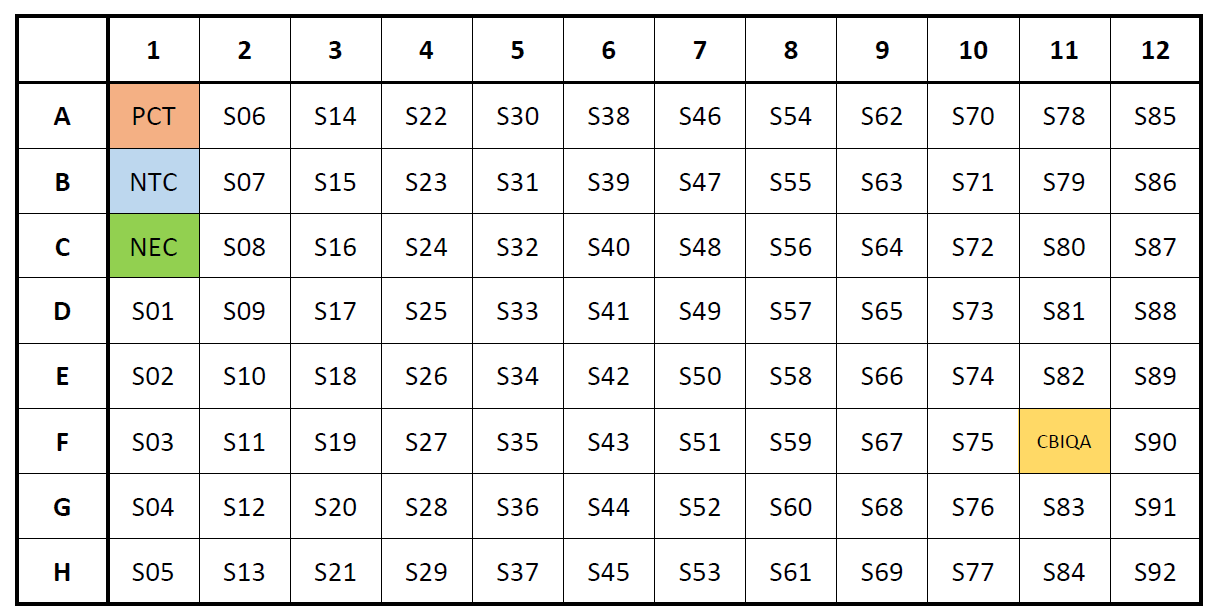


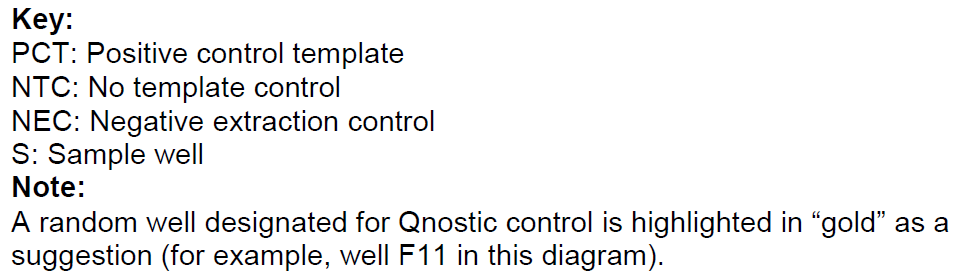


### 6.6.6 Waste Management

- **NOTE: Use of Virkon is not permitted in the sample lysis process or laboratory due to the risk of accidental mixing with lysis buffer containing guanidine thiocyanate, which may result in the release of cyanide gas.**

#### 6.6.6.1 Station 1a

- All plastic packaging waste will be discarded in the plastic biohazard autoclave bags
- Clean outer gloves with Distel (1:10) followed by 70% ethanol and dispose of into the autoclave bag
- Remove the sleevelet and dispose of into the autoclave bag
- Seal the autoclave bag with an autoclave tape ready for autoclaving

#### 6.6.6.2 Station 1b

- Lids may be secured on sample tubes dependant on style and swab placement or left off. Place in the 2L Bio-bin in the BSC. Before removal from the BSC, ensure everything is sealed and double contained using autoclave bag
- Decontaminate the accessible surface of the 96-tube rack with Distel (1:10) followed by 70% ethanol
- Decontaminate any tip boxes for disposal with Distel (1:10) and 70% ethanol and place in the autoclave bag if passed out the hood.
- Any small quantities of waste liquid (less than 50 ml) should be placed in a falcon tube, sealed and disposed of into a Bio-bin within the BSC. Larger volumes of liquid waste from buffers should be poured into a suitable container for autoclaving
- Dry reservoirs should be disposed of in the Bio-bin in the BSC
- Seal the Bio-bins and close the autoclave bag, pass outside the BSC to be placed in a second autoclave bag.
- Clean the outer gloves with Distel (1:10) followed by 70% ethanol and place them into the autoclave bag when being discarded.
- Sleeves to be discarded into an autoclave bag.
- Using clean gloves or a second operator, seal the autoclave bags with an autoclave tape ready for autoclaving.

#### 6.6.6.3 Station 1c

- Keep the waste bag tucked into the corner and stack the used tip boxes (closed) neatly to avoid over-flowing (double check the correct number of tip boxes have been used)
- Small volumes of liquid waste, c. 5 ml can be placed in a 50 ml falcon tube and placed in the autoclave bag for disposal
- Lager volumes of waste, >5 ml should be poured into the Jerry Can in the BSC. Once full the jerry can should be wiped with distel (1:10) and then 70% ethanol and removed from the BSC. A sticker should be affixed to the can, if not already present, denoting the contents of the can. This is then left beside the autoclave collection bins for Avantor to dispose of.
- Dry reservoirs should be disposed of in the autoclave bags
- Pipette tips from the integra should be reloaded into the tip box as per section 6.6.3.23 and the full tip box closed and placed in the autoclave bag in the BSC.
- Waste from the BSC should be placed in an autoclave bag or 6L Bio-bin for the stripettes.
- Seal any Bio-bins if used and close the autoclave bag, pass outside the BSC into a second autoclave bag.
- Clean the outer gloves with Distel (1:10) followed by 70% ethanol and place them into the autoclave bag when being discarded.
- Sleeves to be discarded into an autoclave bag.
- Using clean gloves or a second operator, seal the autoclave bags with autoclave tape ready for autoclaving

# RESPONSIBILITIES

- It is the responsibility of all personnel handling samples to follow this procedure
- It is the responsibility of personnel performing this procedure to ensure all equipment, chemicals, reagents, and solutions are properly labelled and that proper documentation is maintained
- It is the responsibility of the laboratory lead to ensure that this procedure is followed and that it is updated as necessary

# APPENDICES (Selected)

##
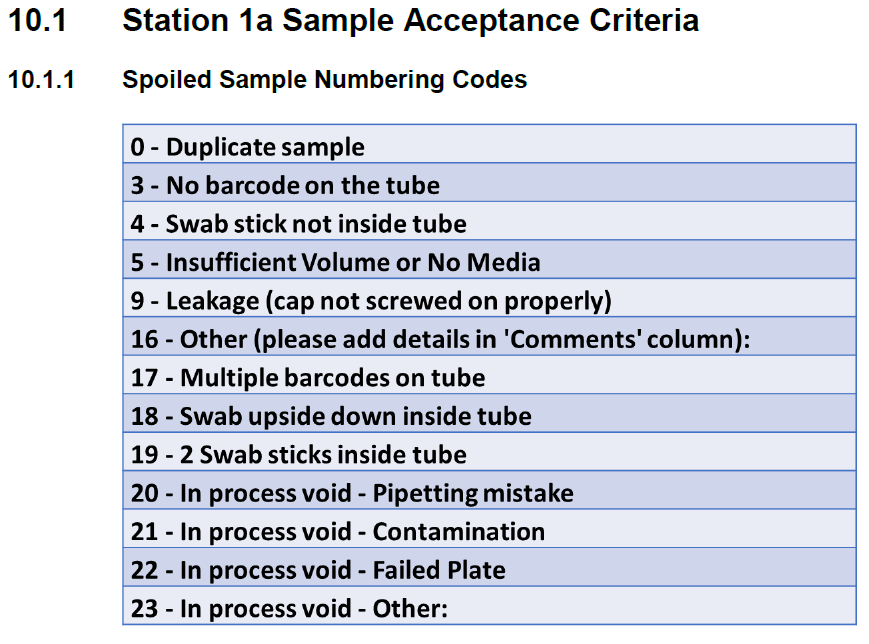


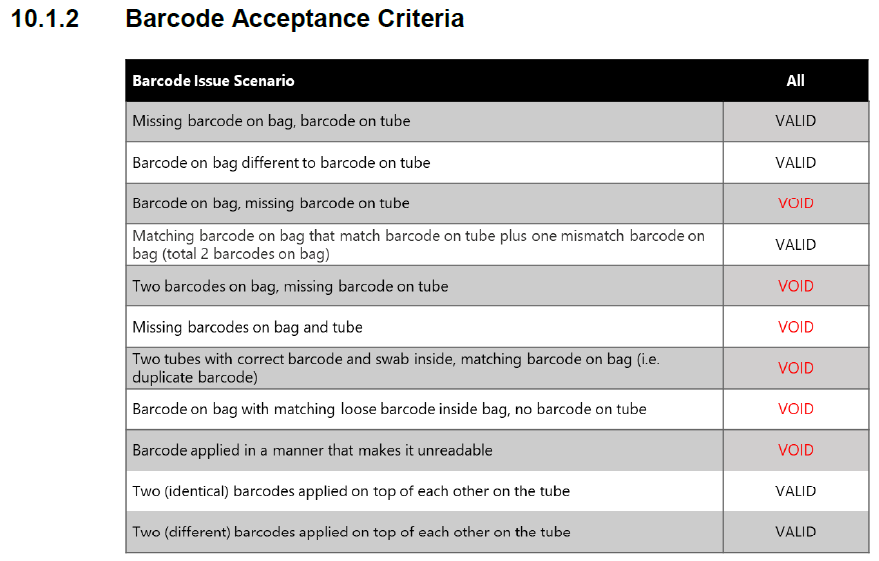


## 10.3 Sample Preparation: Station 1c Manual Process

There are two workflows that can be followed, the manual workflow or one based on the Integra ViaFlo96 for the addition of an all in one lysis buffer to the sample prep using an automated 96 well head pipette. This section covers the manual process should there be a failure of the Integra ViaFlo96.

### 10.3.1 Sample preparation: Station 1c without the ViaFlo96

*Note: This procedure is conducted by two operators. Operator Three will conduct the sample work within the BSC. Operator Four will shadow, checking samples and reagents are added to the correct wells. Where the operator is not defined, this step may be completed by either.*

*Note: For full technical details on working with the LIMS system LIMfinify and Viral Prep Tool App see user guide COVID19 IT training.*

1. Ensure BSC is compliant and within operational safe working limits. All work to be conducted in the BSC unless otherwise stated.

2. Sequentially wipe down inside of BSC with Distel (1:10), RNAse Away and 70% ethanol. Clear up residue at each step with tissue / paper towel and discard in biohazard waste container in the hood.

3. Waste containers consisting of Bio-bins and/or an autoclave bag should be placed in the hood, ensuring the airflow is not obstructed, ready for sample and waste discarding during the process.

4. If opening a new RNAdvance Viral kit, resuspend Proteinase K by adding the appropriate volume of PK Buffer as defined by the manufacturer. 10 ml of PK buffer to 500 mg Proteinase K.

5. Remove the Genesig Easy RNA Internal buffer extraction control (IEC) aliquot from the freezer to defrost.

6. Obtain a 50 ml falcon tube or suitable RNAse/DNase free clean 100 or 250 ml bottle. Operator 3 will prepare the all in one lysis buffer using the following volumes. Operator 4 will verify the addition of the reagents.

*Note: The below calculations take into account a dead volume. These are recommended final volumes to make up, larger plate batches may be made.*

*Note: Once made up, the all in one lysis buffer must be used within 1 hr. Record the time the buffer mix was made up on the bottle.*


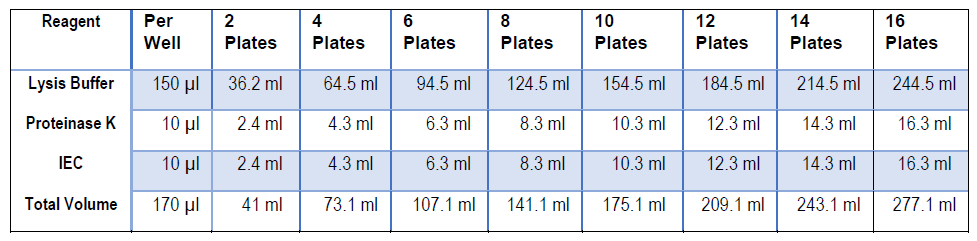


1. Operator 3 will proceed with the following steps. Carefully pour the Lysis Buffer (LBF) all in one, into a 50 mL reservoir and mop any spills immediately.

2. Operator 3: Using a multichannel pipette, add 170μL Lysis Buffer all in one to a row using a P1200 12-channel pipette. Mix the samples 10 times, discard the tips. Repeat with each row of the plate. Operator 4 will witness the addition to each well.

3. Operator 3: Seal plates with microamp clear adhesive seal. Transfer the plate containing samples into a sealable bag to double contain and further into a second bag for triple containment. Wipe with Distel (1:10) and 70% ethanol and remove from the hood or hand to Operator 4.

4. Operator 4: Place the plate in the incubator set to 65°C for 10 minutes.

5. Operator 4: After place the plate at room temperature for 10 minutes then send to RNA.

6. Before sending plates to RNA, they should be visually checked for the correct well volumes and for the presence of a barcode.

7. Contact RNA to push the plates to station 2. Remove the bags from step 5 and place the plate at 4°C or at room temperature on the designated RNA trolley in the internal corridor.

*Note: Plates can remain at 4°C overnight, however by the end of the working day, all plates must be pushed to Station 2. Station 2 will record time and date of arrival if a hold is required overnight.*

8. Repeat from step 11 to run further plates until the buffer is used up.

9. Once complete, Operator 3 will discard any reservoirs or waste into the waste bin in the BSC avoiding any spillage as defined in 6.6.5 Waste Management. Any excess liquid waste from the lysis can be placed in a 50 ml falcon tube and discarded in the biobin or a larger Jerry Can. Clear working area and wipe down surface and pipettes with Distel (1:10) and 70% ethanol as required. Seal the waste bins, close the autoclave bag and pass to Operator 4 for placement into a second bag. Remove outer gloves and dispose of in a new Bio-bin in the BSC.


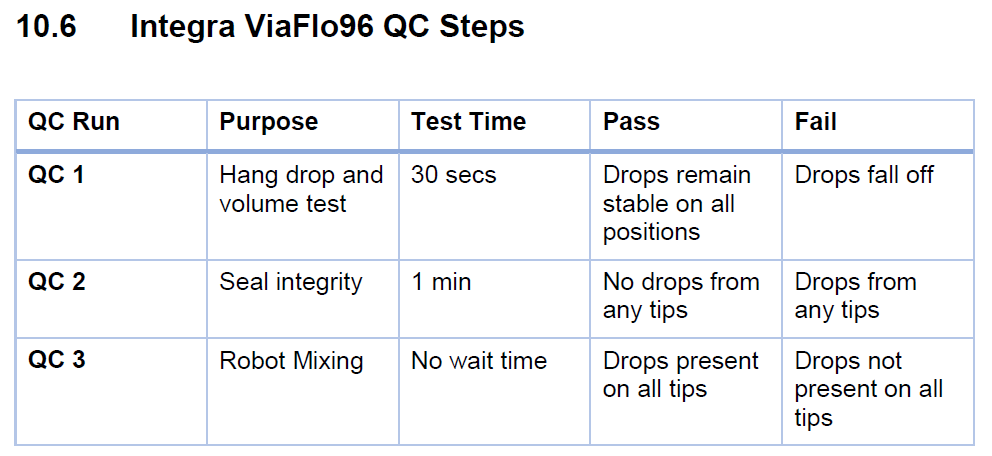


End of SOP CB 02 Version11

# SOP CB 03: RNA extraction from sample plates using the Biomek automated workstation (Version4.1)

# PURPOSE

The Cambridge COVID-19 Testing Centre (CCTC) based at the Anne McLaren Building is one of the National Testing Centres for COVID-19. The purpose of this facility is to test for the presence the SARS-CoV-2 virus. This procedure covers the setup and operation of the Biomek i5/i7 for RNA extraction of viral RNA from human biological samples.

# AUDIENCE

This SOP applies to all on site trained personnel who are responsible for assessing samples for COVID-19 screening.

# SCOPE

This document defines the required procedure at Station 2 (below) for extracting viral RNA from up to 3 x 96 deep-well plates containing human biological samples from nasopharyngeal and oropharyngeal swabs using the Beckman Coulter Biomek i5 or Biomek i7, for the purpose of detecting the presence of SARS-CoV-2 viral RNA using the Beckman Coulter RNAdvance Viral kit.

# GLOSSARY


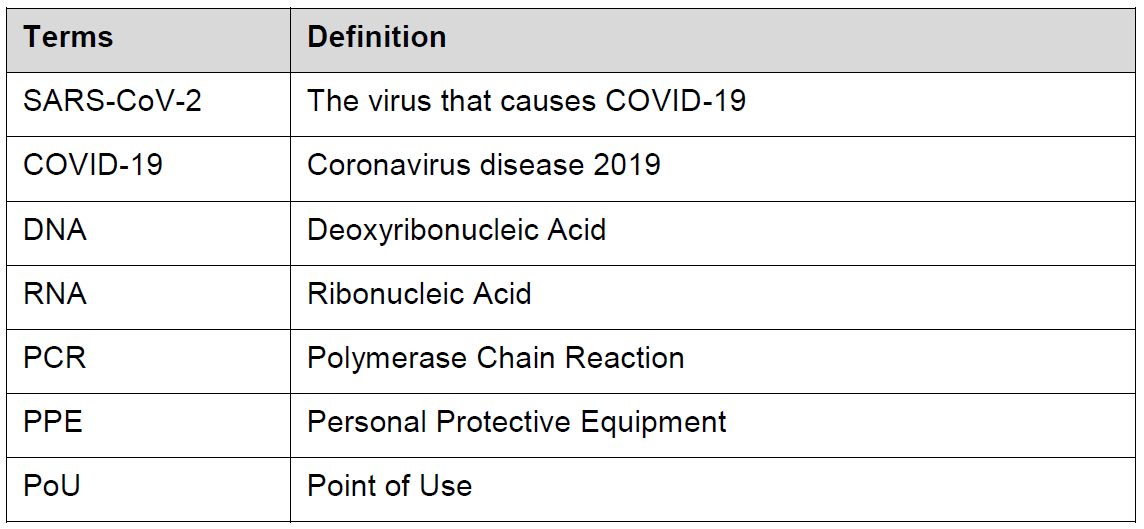


# SAFETY OVERVIEW

## 5.1 Hazards

##
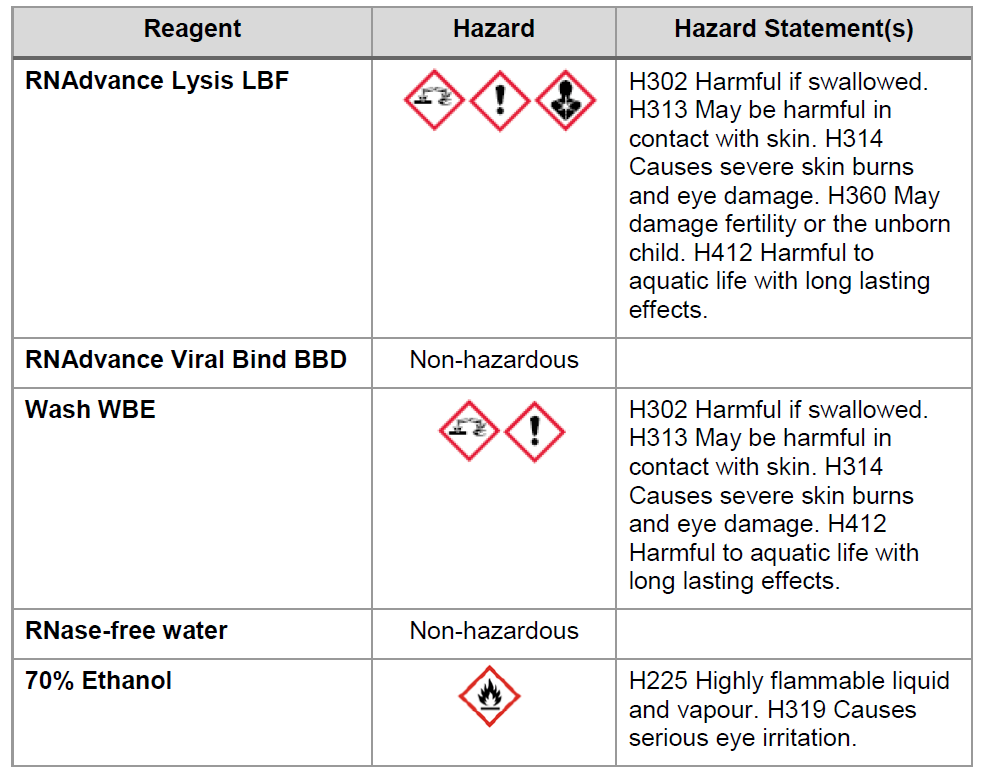

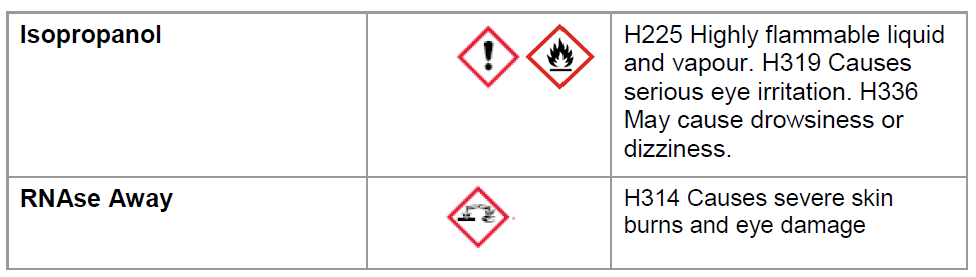


## 5.2 Personal Protective Equipment

- Fully buttoned-up Howie-style laboratory coat
- Standard nitrile gloves
- Laboratory safety glasses or over-glasses

# PROCEDURE

## 6.1 General considerations

### 6.1.1 Warnings and precautions

- Treat all specimens as a biohazard.
- **Refer to the task-based risk assessment**
- Discard all gloves, pipette tips, pipettes, vials, test tubes, or other disposable items into appropriately labelled Biohazard boxes (Bio-bins) or yellow hazardous waste bags.

### 6.1.2 Preventing Contamination

- Change gloves whenever contamination is suspected and when changing workstation
- **NOTE: Use of Virkon is not permitted in the RNA extraction process or laboratory due to the risk of accidental mixing with lysis buffer containing guanidine thiocyanate, which may result in the release of toxic gas**

**6.1.3 Preventing DNase/RNase contamination**

- Use DNase/RNase free disposable plasticware and pipettes reserved for RNA work to prevent cross-contamination with DNases/RNases from shared equipment
- Use DNase/RNase-free filter tips throughout procedure to prevent aerosol and liquid contamination
- Wipe surfaces of workbenches with RNAse Away followed by 70% ethanol before use
- Change gloves frequently

### 6.1.4 Exposure control plan

The risk of SARS-CoV-2 exposure during this procedure is very low due to the viral inactivation steps carried out in the previous steps (lysis and heat inactivation; SOP CB 02). Nonetheless, to err on the side or caution, the following measures should be taken in the case of a sample spillage or any other risk of exposure to SARS-CoV-2.

- Cover the spill with 70% ethanol and a liquid absorbent pad or tissue depending on the volume
- Incubate for 30 minutes then remove the absorbent pad or tissue and dispose of in a hazardous waste bag
- Clean the spill area with 70% ethanol and wipe dry, disposing of wipes in a hazardous waste bag for disposal.
- If the spill was in an RNAse free working area (e.g. plastic tray) then clean with RNAse Away followed by 70% ethanol
- Change gloves following dealing with a spillage
- If PPE is contaminated, remove PPE and place in an autoclave bag. Wash hands or any exposed skin with soap and warm water.
- Refer to Risk Assessments for more information.

## 6.2 Procedure Schematic

##
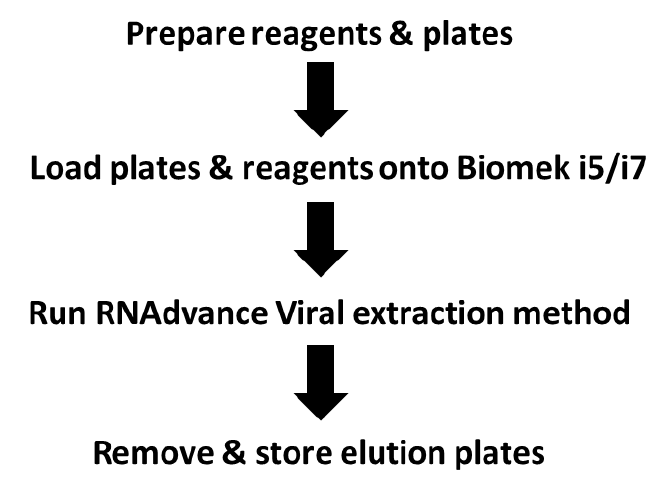


## 6.3 Workstation Schematic


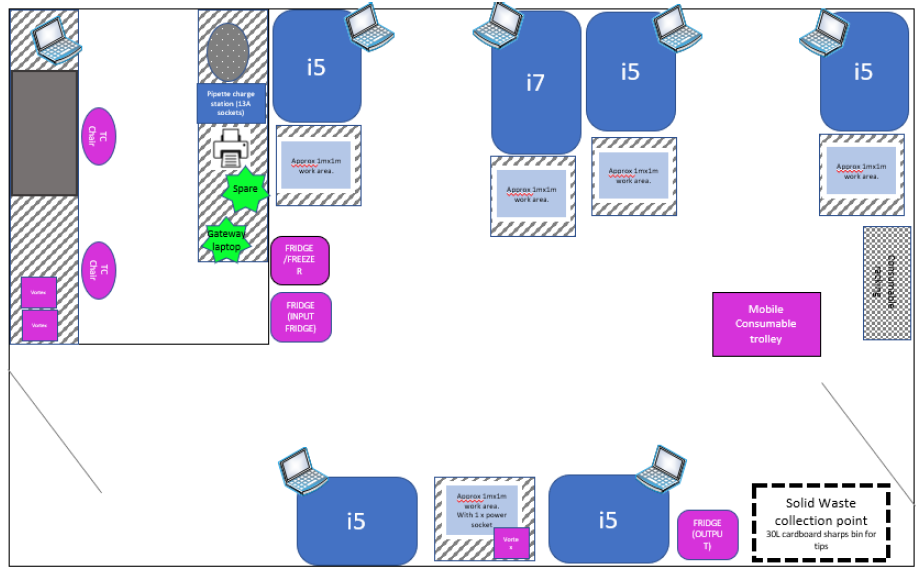
For reference, a typical layout of a Station 2 RNA Extraction Laboratory.

## 6.4 Materials and Reagents

**Note**: the labware listed below is needed for one 3-plate run


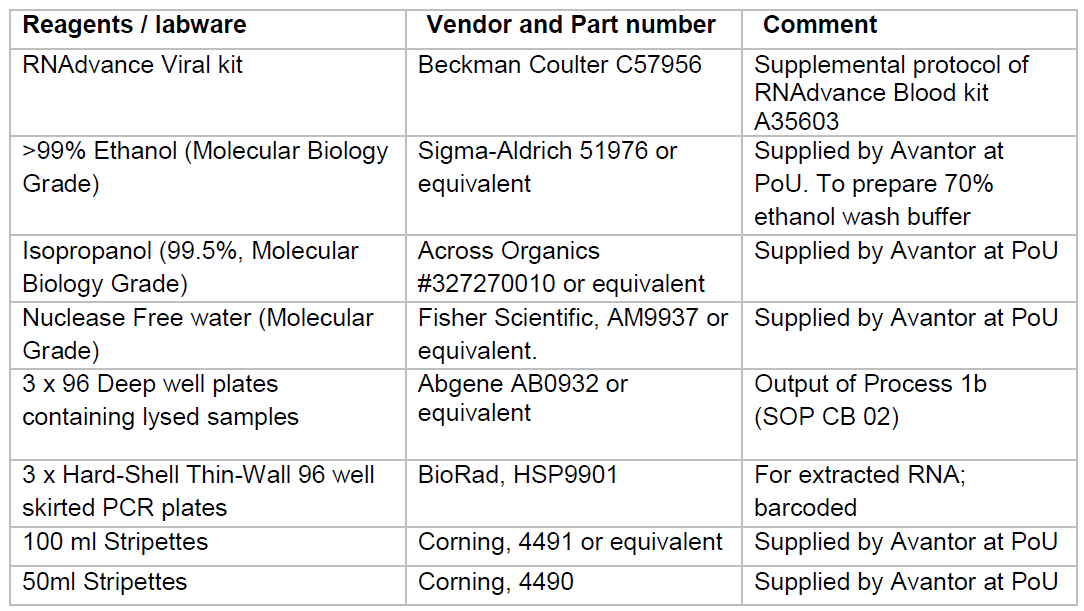

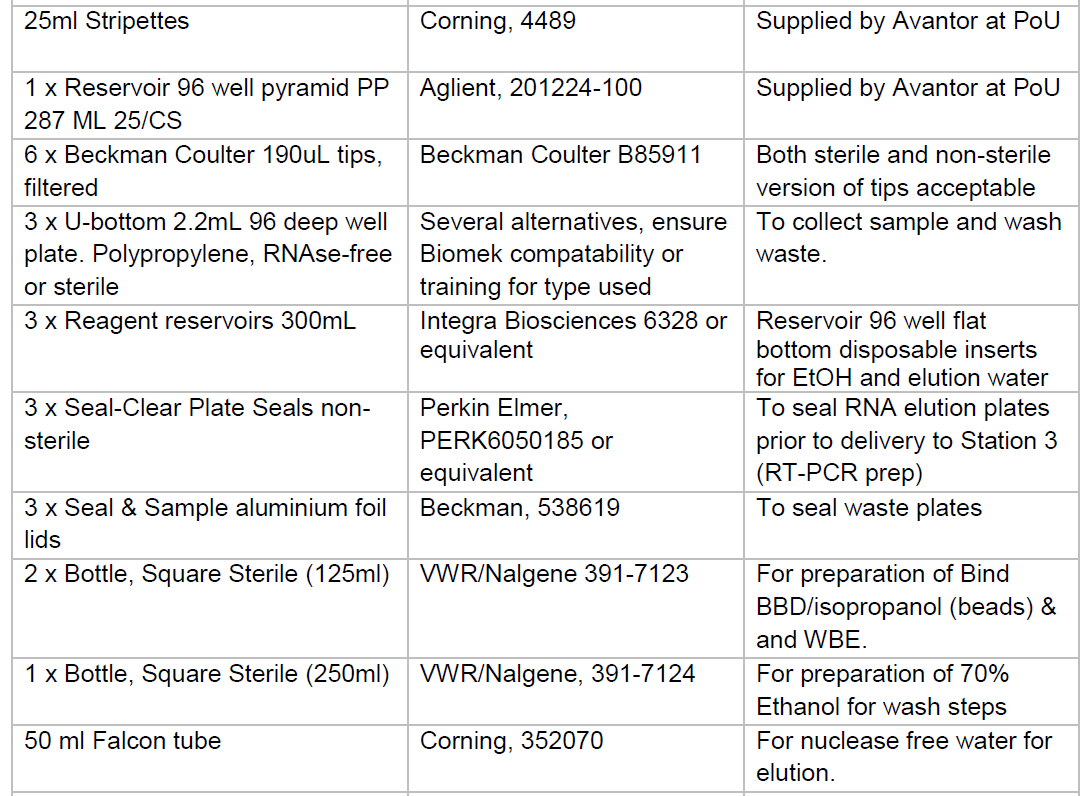

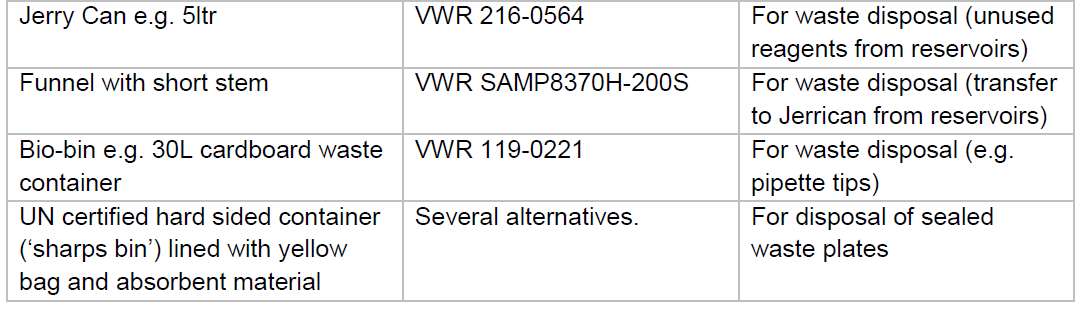


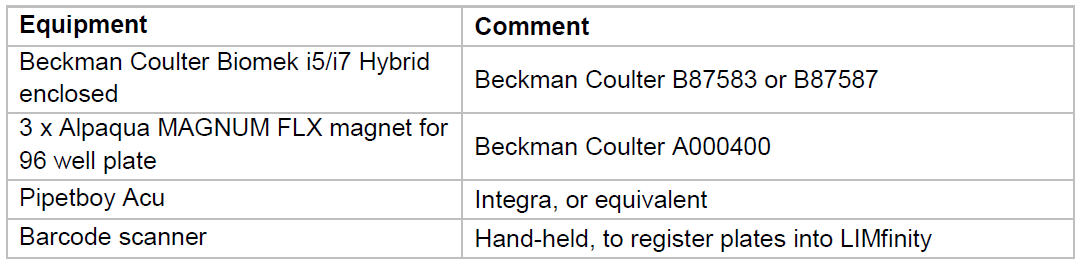


## 6.5 General guidelines

- Perform all steps at room temperature (20-25°C), unless otherwise noted
- Perform all steps wearing appropriate PPE
- The sample received from station 1b (SOP CB 02) has been lysed in RNAdvance Lysis LBF and the Wash Buffer WBE contain guanidine thiocyanate. Care must be taken in handling and disposing of liquid waste. **Do not mix with Virkon**. Refer to Hazard Statements, the information above and Risk Assessments
- All liquid waste should be disposed of in the correct receptacles: plates containing waste from the Beckman i5/i7 (containing guanidine thiocyanate and ethanol) are sealed using an adhesive film lid and stored for collection and special disposal. Liquid waste from reservoirs is collected in a Universal Jerry can for collection and special disposal
- Zero waste is to go to drain except for pure water
- All waste tips and items with the potential to puncture or tear a plastic bag should be placed in hard-sided bin (e.g. Bio-bin) and sealed before disposal
- All other solid waste including reservoirs, packaging, empty plates & used plastic reagent bottles should be placed yellow bags and closed with a cable tie before disposal

## 6.6 Procedure

### 6.6.1 RNA extraction

Protocol for processing three sample plates in one Biomek i5/i7. See appendix (10.5) for reagent volumes and deck positions to be used for 1 or 2 sample plate runs using this method. 50 ml nuclease free water is used for elution in all cases (1-3 Sample plate run).

**Important Note**: The Biomek i5/i7 is equipped with a light curtain at the front opening which if broken during a run will immediately pause the run. If this is done accidentally the run can be restarted by clicking continue in the dialogue box. If this is done promptly it will not negatively affect the run. Once a run is in progress, it is recommended to close the door to avoid accidental breaking of the light curtain.

1. At start of day ensure that the Biomek i5/i7 is turned on (on/off button on the back right side), if not left on continuously. Note: The Biomek i5/i7 is equipped with a LED light at the top of the instrument. Blue= Idle, Green= Running, Amber = Set-up, Red= Error.

2. Switch on the attached computer. Note: the order in which the Biomek i5/i7 & computer are switched on is not important. The password is taped below the screen or available from the Lab lead.

3. Home the Biomek i5/i7 and run the “QC Run” (see Appendix 10.4) if this is the first run of the day.

4. Wipe down all worksurfaces with RNAse away and 70% ethanol.

5. The RNA Extraction Lab Lead should query the LIMfinity system for active sample plates (referred to as Viral plates in LIMfinity) and check the plate storage fridges for sample plates that may have been left overnight from the previous shift. Any sample plates in storage must be processed within 12 hours of arrival.

6. Prepare reagents required for the run when informed of expected sample plate arrival (up to 1 hour before use). See appendix 10.5 for the correct reagent volumes to use for 1-3 Sample plate runs on a Biomeki5/i7. The reagent lot number barcode cards (for quick scanning into LIMfinity) are checked against current reagent lot numbers to confirm that they are correct to use at start of shift and the card is updated when any new reagent bottles are opened for use.

7. In the reagent preparation area, remove the RNAdvance Viral Bind BBD bead bottle (Beckman C42156, contains 4.6 ml when full) shake & vortex thoroughly to ensure the beads are fully resuspended. Using clean nuclease-free pipette, transfer 2.0 ml of the resuspended beads to 80 ml isopropanol in a 125 ml Nalgene bottle and mix well.

8. Prepare WBE wash buffer by adding 225 ml isopropanol to the bottle as per manufacturer’s instructions (Wash WBE, Beckman C42172). Add 150 ml of this WBE/isopropanol mix to a 250 ml Nalgene bottle.

9. In the reagent preparation area, make up 270 ml of 70% ethanol for addition to the wash reservoir by adding 189 ml ethanol (Molecular Grade) to 81 ml nuclease-free water in a 250 ml nuclease-free sterile bottle. Use sterile individually wrapped 100 ml stripettes for this to avoid nuclease contamination of the wash buffer.

10. Pour 50ml of nuclease-free water into a 50 ml Falcon tube for elution.

11. Place reagents on the plastic tray in the working area of the Biomek to be run.

12. Collect sealed 96 deep-well sample plates from Station 1b. Sample plates should be moved between stations using a high-sided (blue) trolley lined with spill mats. When collecting sample plates, do not open the door of the Sample preparation laboratory; instead ring the bell and wait for the door to be opened by the Station 1 team member, who will place the sample plates onto the mats on the trolley. Carefully wheel the trolley back to the RNA Extraction laboratory for processing. If sample plates have come out of the fridge, they should be allowed to equilibrate to room temperature for 10 minutes before proceeding with the RNA extraction.

Note: Sample plates can be stored in the designated fridge in the RNA Extraction laboratory prior to processing, for example if all instruments are in use or if there is insufficient time to complete the RNA extraction before the end of the day. The start time of fridge storage should be noted on the plate in marker pen, and the plates should be processed as soon as possible. As in step 5, any plates stored in the fridge should be allowed to warm to room temperature for 10 minutes before processing.

13. At the computer controlling the Beckman i5/i7, open the Beckman Coulter Method Launcher from the desktop (this icon has a green arrow).

14. Click the ‘RNAdvance Viral-3 Plate Method’ start the user interface for the automation set-up.

15. Select the number of sample plates to be processed: either 1, 2 or 3. The software will then calculate labware, reagent positions and volumes based on this. The description below is for 3 sample plates, please follow the onscreen instructions for labware positions with 1 or 2 sample plate runs.

16. Keep default settings for all other choices in workflow and method options.

17. Click “Start Run” to continue to deck setup and reagent preparation.

18. Verify that the deck is clear, then click Continue.

19. The Guided Labware Setup for the placing labware/reagents & samples will be initiated. Step by step, populate the Biomek i5/i7 deck with tips and plates.

**Note**: The lids should only be removed from the tip boxes within the Biomek i5/i7, but should be done immediately after addition to the deck to avoid the risk of them being accidentally left in place. Set the lids aside to re-lid the tip boxes at the end of the run (see step 35).

**Note:** Once installed the Magnum FLX Magnets should be left in place and not be removed between runs.


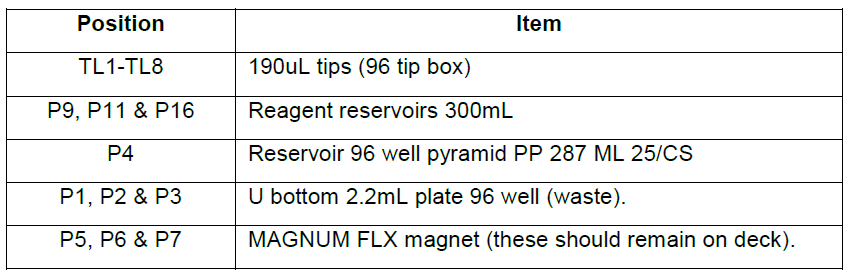


20. Follow the Guided Labware Setup for dispensing the following reagents, carefully pouring them into the relevant reservoirs. (Note: beads added last-step 25 below)


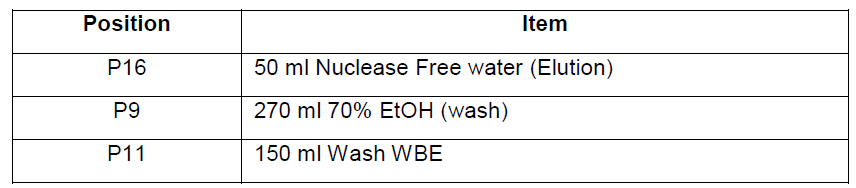


21. Ensure that all sample plates and final (elution) plates (96 well skirted PCR plates) have a barcode located on the left-hand short side.

22. Label the three sample plates to be processed 1, 2 & 3 and label three barcoded final plates 1, 2 & 3. This is to ensure correct pairing of the Sample Plate and RNA Plate in LIMfinity and when subsequently placed on the deck of the Biomek i5/i7.

23. Log in to LIMfinity in the gateway laptop computer equipped with a barcode scanner.

24. Scan the Sample and Final plates pairwise, and load on the deck as follows: This process should be witnessed by another team member to ensure the correct procedure and positioning is achieved.

a. Select ‘RNA Extraction’ from the left-hand side menu to open the window to record new RNA plates. Scan Sample Plate 3 and Final Plate 3 into a LIMfinity as a pair and place in their correct positions on the i5/i7 deck (P7 & P15 respectively). Place Final Plate 3 on the deck first, then wear double gloves to remove the film seal from Sample Plate 3, place on the deck and remove & dispose of the outer gloves. Scan the instrument ID and reagent lot numbers into the relevant windows in LIMfinity. Click ‘OK’ to save the plate.

b. Repeat this procedure for Sample Plate 2 and Final Plate 2 (placed on P6 & P15 (on top of elution plate 3) respectively).

c. Repeat this procedure for Sample Plate 1 and Final Plate 1 (placed on P5 & P15 (on top of elution plate 2) respectively).

- Note: If the Sample Plate has “Addenbrookes Samples” written on it the used must write the same on the Final Plate (on the side of the plate & on the film after sealing). This is to let the PCR & Data teams know to export the Ct values from FastFinder as these are required by Addenbrookes.

25. Shake the 82 ml of Bind BBD Beads again to ensure they are thoroughly resuspended and pour into the reservoir in position P4.


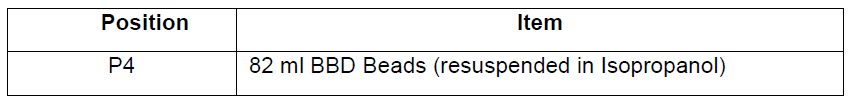


26. Once all steps have been followed, the “Final deck setup” page is displayed and should look like this. Both the user and witness should then confirm that the deck setup and reagent volumes are visually correct. (Beckman i5/i7 deck layout shown below, note that the i7 has extra unused positions to the right)


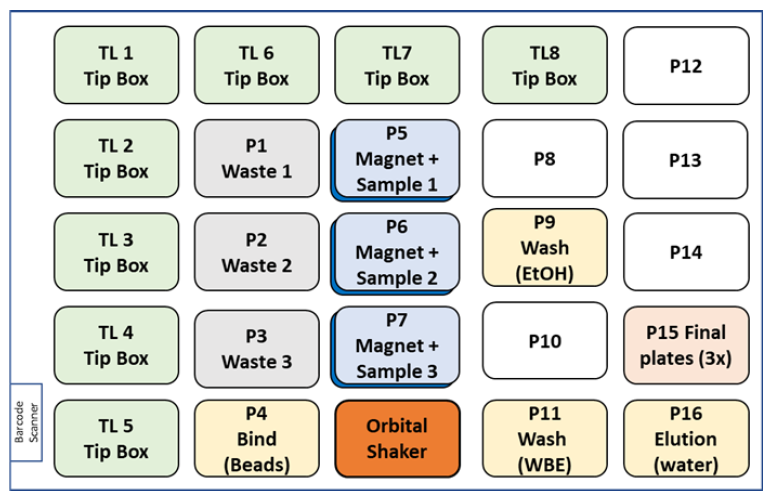


27. Continue to “Deck Verification”. Verify that the setup on the deck corresponds to the setup in the software. If a warning message is given, check carefully including checking with a witness that it is OK to ignore the automatic warning message. Click “Finish” to start the method. The light at the top of the instrument should turn green.

28. Close the instrument door to prevent accidental breaking of the light curtain.

29. When the method has finished a “Method Completed Successfully” dialogue box will appear and the light on top of the instrument should turn blue (idle). Click OK to acknowledge.

30. After the process has fully completed, carefully seal the RNA plates with the clear adhesive seal and take directly to Station 3 (pre-PCR) for qPCR setup. If not taken for proceeding directly, the plates should be stored in the pre-PCR room fridge.

**Note:** If the RNA extraction run ends too late in the evening for pre-PCR to process, RNA plates can be stored overnight in the fridge of the pre-PCR room for processing the following morning. In this event please inform the PCR Lab

Lead and write the time & date the RNA plate was completed on the film seal, so that these plates can be processed first the following morning.

31. Seal the waste plates on the deck with aluminium foil seals and then remove from the Biomek. Roller over the film to make a good seal, and place into a yellow bag within the designated yellow plastic waste bin for removal.

32. Carefully empty any remaining liquid from the reagent reservoirs into the liquid waste container (UN certified Jerrican) using the plastic funnel.

33. Re-lid all tip boxes whilst on the deck and place them carefully into a 30L yellow Bio-bin.

34. Discard all empty reservoirs & sample plates into yellow bags. When full, close bags with a cable tie and transfer to the waste collection point for disposal.

### 6.6.2 Error management

- If at any point during the run the user realises there could be a potential problem with the way the labware is setup or other serious issue they should break the light curtain or click on the pause button to pause the instrument and seek the assistance/advice of the RNA Extraction Lab Lead for that shift. The Lab Lead will decide the course of action to take e.g. submit an instrumentation support request.
- If at any point during the run an error message appears (indicated on screen and/or by red light on top of instrument) the user should break the light curtain (if instrument is not already paused) and seek the assistance/advice of the RNA Extraction Lab Lead for that shift. The Lab Lead will decide the course of action to take.
- If before, during or after a run an incident occurs (e.g. plate dropping) with the potential to result in a plate needing to be partially or fully cancelled the operator should contact the RNA Lab Lead for that shift. If appropriate and after consultation with the Shift Lead, the RNA Lab Lead will cancel the RNA plate in LIMfinity and inform the Sample Prep Lab Lead so that they can void the samples in LIMfinity. The RNA Lab Lead will inform the Shift Leader of any incidents so that it can be logged in the incident log.

### 6.6.3 Waste Management

- Sealed waste plates from the Biomek are disposed of within the designated yellow plastic waste bin for removal from the laboratory. **Note:** Adsorbent material (e.g. ‘pig mat’) should be placed in the bottom of the yellow bag prior to adding the sealed waste plates and again on top when the bag is full prior to tying off with a cable tie. The yellow bin should be labelled: “RNA Extraction Waste. Sealed multi-welled plates from RNA extraction containing aqueous ethanol, isopropanol and guanidine thiocyanate”.
- Liquid from reagent reservoirs is collected in UN certified Jerrican liquid waste containers. The container should be labelled “Bead mix consisting of 35% ethanol, 20% isopropanol, traces of guanidine thiocyanate”, and display flammable liquid and corrosive substance hazard labels. When full (see mark on side of can) place in waste collection point in wheeled containment trolley for removal.
- Re-lidded tip boxes are disposed of in 30L yellow Bio-bins. When full, place the sealed Bio-bin into a yellow hazardous waste bag, close with a cable tie and transfer to the solid waste collection point for disposal.
- All other waste is collected in yellow hazardous waste plastic bags and when full bags are closed with a cable tie and placed in the waste collection point for disposal.

# RESPONSIBILITIES

- It is the responsibility of all personnel handling samples to follow this procedure.
- It is the responsibility of personnel performing this procedure to ensure all equipment, chemicals, reagents, and solutions are properly labelled and that proper documentation is maintained.
- It is the responsibility of the Laboratory Lead and Shift Lead to ensure that this procedure is followed and that it is updated as necessary.

# APPENDICES (Selected)

## 10.4 Performing a QC run of i5/i7 and the start of the day

1. Initiate QC run on the instrument using Method Launcher (Image of a small Scientist).

2. Set the deck up using the reference image on the software and labels on the deck. You will need

- 1 x Tip Box
- 1x Integra Reservoir
- 1 x Sample Plate
- 1 x 100 ml H2O

3. Once the deck is set- click “OK” on the software

4. What are you looking for:

i. Ensuring liquid is aspirated, 100μl should be aspirated with a 2 μl air gap. Ensure that all tips have equal volumes of water. The water will hold for 90 seconds – ensure that no drips or loss of liquid occurs in this time.

ii. The tips will line up with the sample plate, check that this is correctly aligned. The tip head will then perform a tip touch on the bottom of the sample plate with the sample tips, check that it slightly & evenly depresses the sample plate on the magnets (note this may vary in extent per Biomek, the intention is that full contact is made between the tips and plate).

iii. The sample plate will move from sample position 1 to the Orbital Shaker & back and from Sample position 2 to the Orbital Shaker & back and from Sample position 3 to the Orbital Shaker & back – check that the plate moves with ease and is placed into position correctly at both orbital shaker & magnet (no clipping).

5. If any of the above actions show an issue alert your Lab Lead.


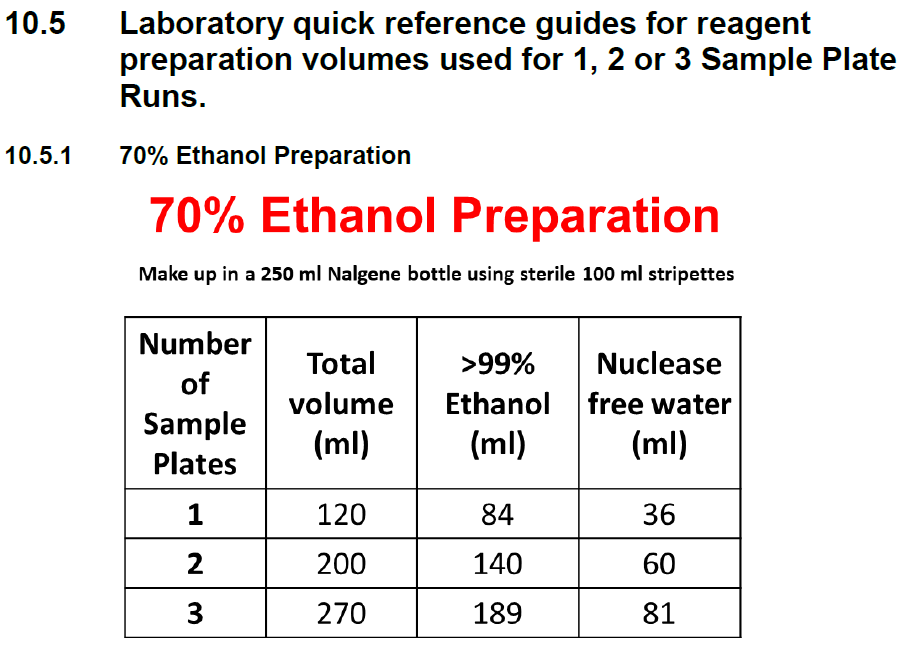


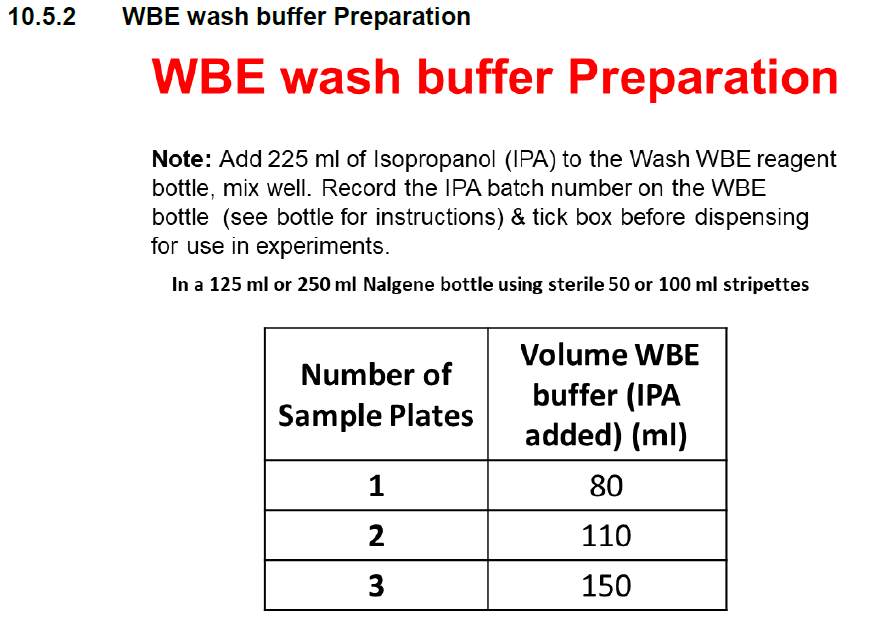


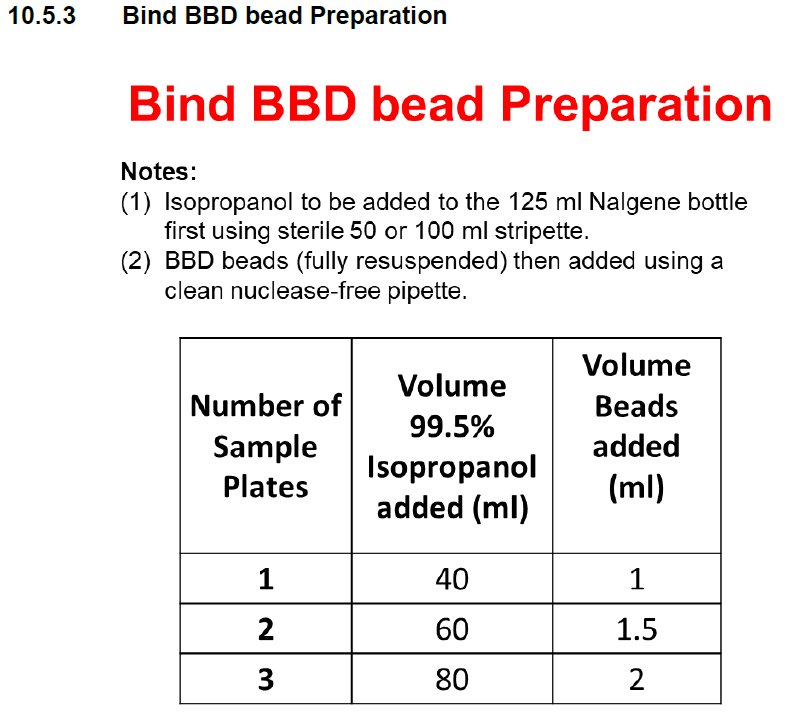


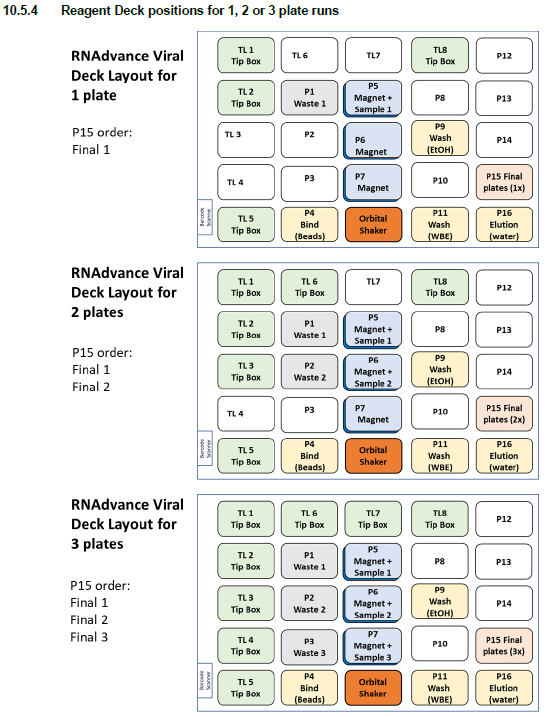


End of SOP CB 03 (Version4.1)

**SOP CB 05: STATION 3 USE AND STORAGE OF HT KIT REAGENTS FOR PREPARATION OF 384-WELL PCR PLATE AND ADDITION OF NEGATIVE CONTROL AND RNA TEMPLATE (Version5.2)**

1. **PURPOSE**

The Cambridge COVID-19 Testing Centre (CCTC) based at the Anne McLaren Building is one of the National Testing Centres for COVID-19. The purpose of this facility is to test for the presence the SARS-CoV-2 virus. This procedure provides the method for preparing and dispensing the RT-PCR Master Mix, the addition of the No Template Control (NTC) and PCR plate storage prior to the addition of the RNA samples.

1. **AUDIENCE**

This SOP applies to all on site trained personnel who are responsible for assessing samples for COVID-19 screening.

1. **SCOPE**

This document defines the required procedure for Station 3 for preparing the Primerdesign Ltd COVID-19 Genesig Real-Time PCR assay Master Mix and dispensing it to the 384-well PCR plate, the addition of the No Template Control (NTC) and PCR plate storage. It also covers plate to plate transfer of the RNA samples with the purpose of detecting the presence of nucleic acid from SARS-Cov-2 in oropharyngeal and nasopharyngeal swabs. The assay can be run as a 20ul or 10ul total assay volume in a 384 well qPCR plate.

1. **GLOSSARY**

| **Terms** | **Definition** |
| --- | --- |
| SARS-CoV-2 | The virus that causes COVID-19 |
| COVID-19 | Coronavirus disease 2019 |
| DNA | Deoxyribonucleic Acid |
| RNA | Ribonucleic Acid |
| PCR | Polymerase Chain Reaction |
| RT-PCR | Reverse Transcription – Polymerase Chain Reaction |
| PPE | Personal Protective Equipment |
| BSC | Biological Safety Cabinet |
| PCT | Positive Control Template |
| NTC | No Template Control |
| NEC | Negative Extraction Control |
| IEC | Internal Extraction Control |
| min | minute |

1. **SAFETY OVERVIEW**

# Hazards

| **Reagent** | **Hazard** | **Hazard Statement(s)** | **Reference** |
| --- | --- | --- | --- |
| **Primerdesign Ltd COVID-19 Genesig Real-Time PCR**  **assay** | Non-hazardous |  | MDC COSHH 064 |
| **70% Ethanol** | 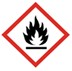 | H225, H319 | MDC COSHH 048 |
| **Distel (1:10)** | Non-hazardous | H315, H317, H318, H351, H373, H412 | MDC COSHH 004 |

- 1. **Personal Protective Equipment**
     - Fully buttoned-up Howie-style laboratory coat
     - Standard nitrile gloves
     - Laboratory safety glasses or over-glasses

1. **PROCEDURE**

# General considerations

## Warnings and precautions

- - - - Treat all specimens as a biohazard.
      - Refer to the task-based risk assessment.
      - Discard all gloves, pipette tips, pipettes, vials, test tubes, or other disposable items into appropriately labelled sharps biohazard boxes or autoclave bags.
      - Use separated working areas for specimen preparation, reaction set up and amplification, with separate supplies and equipment

## Preventing Contamination

- - - - Incorrect results could occur if either the clinical specimen or the real-time PCR reagents used in the amplification step become contaminated by accidental introduction of amplification product (amplicon) or positive control reference material.
      - The Genesig COVID-19 positive control template is provided in a sealed foil envelope and contains a high copy number of templates. It should be opened and processed away from test samples and kit components to avoid cross- contamination.
      - Change gloves between samples and whenever contamination is suspected.
      - Keep reagent and reaction tubes capped or covered as much as possible.
      - Always check the expiration date prior to use. Do not use expired reagent. Do not substitute or mix reagent from different kit lots.
      - Work surfaces, Biological Safety Cabinet, pipettes and centrifuges should be cleaned and decontaminated with cleaning products (e.g. DNA/RNA remover, 70% ethanol, Distel 1:10) to minimize risk of nucleic acid contamination.
      - Use UV lighting in the BSC’s used for handling/dispensing Master Mix and PCT to decontaminate cabinets in the case that contaminated plates are identified during the data analysis or a spill of the PCT occurs within the BSC.
      - Change gloves whenever contamination is suspected and when changing work station
      - Reagents and equipment should not be moved from a dirty area to a clean area. Should a case arise where a reagent or piece of equipment needs to be moved backwards, it must first be decontaminated with Distel and wiped down with 70% ethanol.

## Preventing Dnase/RNAse contamination

- - - - Use Dnase/Rnase-free disposable plastic ware and pipettes reserved for DNA/RNA work to prevent cross-contamination with Dnases/Rnases from shared equipment.
      - Use Dnase/Rnase-free filter tips throughout the procedure to prevent aerosol and liquid contamination.
      - Wipe surfaces of workbenches and biological safety cabinets with RNAse away followed by 70% ethanol.
      - Where RNA samples are not able to be processed as soon as possible for example in the event of Bravo failure, maximum capacity reached, awaiting further RNA plates for processing onto the same PCR plate or during shift handovers, the sealed RNA plates may be stored at 4^0^C in a refrigerator for a maximum of 12 hours, prior to RT-qPCR set up. Storage will be in the ‘RNA Input’ fridge in PCR-Prep (station 3b), the plates will be fully sealed and the time of preparation clearly written on the seal.

# Procedure Schematic

Sort into the 3 components (each in separate sealed silver pouches); PCT, IEC and Primer/probe/Master Mix.


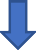


Before use of all reagents, check expiry date.


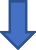


Thaw and prepare reconstituted RT-PCR mix and Primer/Probe and record lot numbers in DailyPCRReagentsSheet.xlsx file.


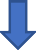


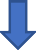

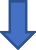
Dispense RT-PCR Master Mix into 384-well PCR plate using MultiDrop. Add RNase/DNase Free water to NTC wells and seal PCR plate,

If necessary, store PCR plate at +4°C prior to addition of RNA samples.


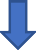


Remove tips in position A1 and B1 (corresponding to positive and no template controls) for filtered 96-tips boxes and position them on the Bravo deck.


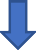


Scan RNA sample 96 well plates (up to 4) and 1x RT-qPCR plate 384-well plate filled with Master Mix and position on the Bravo deck.


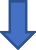


Run the 96 to 384 stamping program on the Agilent Bravo fitted with a 96LT head to dispense RNA sample into the RT-PCR Master Mix.


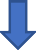


Loosely seal the RT-PCR 384-well plate with LightCycler 480 sealing foil.


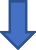


Register Bravo transfer, MultiDrop head, Master Mix and PCT batch numbers into LIMfinity.

# Work Station Schematic


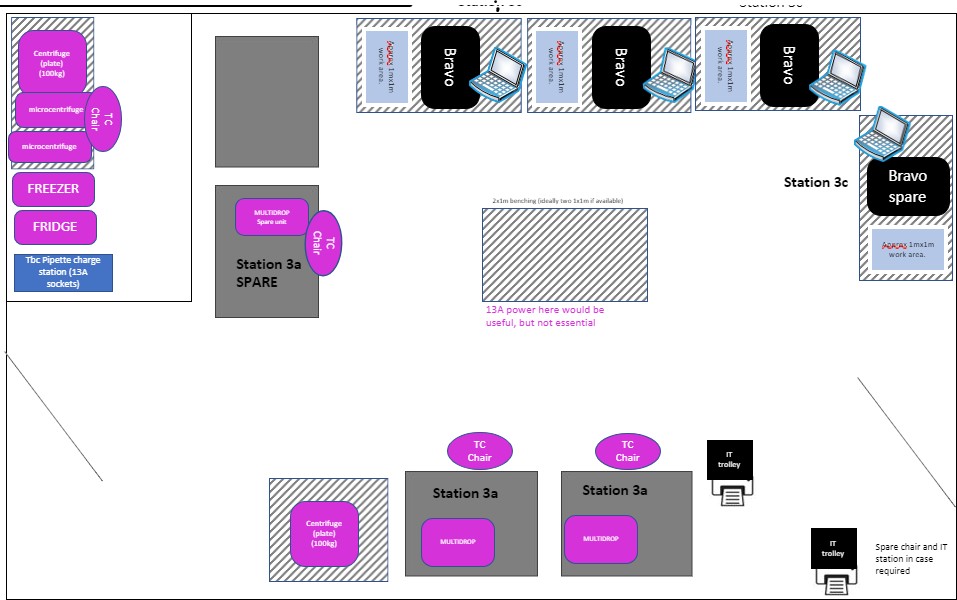


For reference, a typical layout of a Station 3 RT-PCR Preparation Laboratory

# Materials and Reagents

| **Reagent / Labware** | **Comment** |
| --- | --- |
| Reconstituted Oasig™ OneStep 2X RT-qPCR  Master Mix | From PrimerDesign LTD COVID-19 RT qPCR assay kit |
| Reconstituted COVID-19 Primer & Probe Mix  (including IEC primer/probe mix) | From PrimerDesign LTD COVID-19 RT qPCR assay kit |
| Rnase/Dnase Free water 1.5 mL vial | From PrimerDesign LTD COVID-19 RT qPCR assay kit |
| P1000 tips, sterile, filtered |  |
| P20 tips, sterile, filtered |  |
| Plate sealer | eg. Clear Adhesive Film PerkinElmer Topseal-A plus Cat #6050185 |
| Rnase/Dnase Free water | To clean MultiDrop cassette |
| Distel |  |
| Ethanol 70% spray |  |
| RNAse away spray | e.g. Molecular BioProducts Cat #7002 |
| Ethanol 100% | To prepare 70% solution for cleaning MultiDrop cassette |
| Aluminium foil | To protect RT-PCR mix from light |

| **Equipment** | **Comment** |
| --- | --- |
| BSC hood dedicated for PCR Master Mix  preparation |  |
| Fridge (+4°C |  |
| Freezer (-20°C) |  |
| Electronic single channel Multidispense pipette  P200 + carousel charging stand |  |
| Single channel pipette P1000 |  |
| Single channel pipette P20 |  |
| Vortex |  |
| MultiDrop Combi | Thermo #5840300 |
| Small tube plastic tips dispensing cassette for  MultiDrop Combi | Thermo # 24073291 |
| Waste container for tips and tubes | Biobin |
| Ice box containing ice |  |

- 1. **General guidelines**
     - Perform all steps in a BSC to avoid contamination unless otherwise noted
     - Perform all steps wearing appropriate PPE
     - Avoid creating bubbles during mixing and aliquoting

# Procedure

- - - The Primerdesign Ltd Genesig Real Time PCR COVID-19 High Throughput (HT) assay kit is shipped at ambient temperature but must be stored at -20°C upon arrival.
    - Always check the expiration date prior to use. Do not use expired reagents.
    - **Protect fluorogenic primer/probe mix from light.**
    - The kit components from packets 1 (Primer/probe mix and the enzyme master mix) and 3 (RNA internal extraction control) are all delivered ready to use in solution. The positive control template (packet 2) is delivered lyophilized and must be resuspended in the appropriate, supplied template preparation buffer to the correct volume as detailed in the table below, prior to use.
    - Once resuspended, components are stable for up to six months if stored at -20°C and for 3 months at 4°C or the expiry date, whichever occurs first.
    - Whilst in use, the kit components should be kept on ice or stored at 4°C in the dark to minimize the time at room temperature.
    - The reagents should not be freeze/thawed. Once defrosted the components are stable at 4°C for up to 3 months according

to manufacturer’s specifications. Validation data at our testing centre has confirmed 24 hour stability and therefore good practice within the centre would be to use the thawed components within 24 hours.

- - - Each time a component is defrosted, the date will be written on the outer packaging/container if it is not used immediately.
    - There is a Master Mix inventory located in the internal Teams: Cambridge Coronavirus test Centre Teams/3.PCR/Files/Inventory Master Mix where all current

lab stock is logged along with its location, Lot number and expiry date. As a kit is used, the stock will be deleted from the inventory.

## RT-qPCR components

One kit of Primerdesign Ltd Genesig Real Time PCR COVID-19 High Throughput (HT) assay kit assay will enable the testing of 1536 samples (4 x 384-well qPCR plates) if using a 20µl assay, or 3072 samples (8 x 384-well qPCR plates) if using a 10µl assay and contains the following:

| **Reagent label** | **Number of Vials 1536 tests** | **Volume (mL per vial)** | **Lid color** | **Resuspended with?** |
| --- | --- | --- | --- | --- |
| **Pack 1** | | | | |
| PrecisionPLUS OneStep qPCR Master Mix | 1 | 19.0 | Orange | n/a |
| COVID-19 Primer & Probe Mix (including IEC primer/probe mix) | 1 | 3.8 | Green | n/a |
| **Pack 2** | | | | |
| Genesig COVID-19 Positive control template | 1 | 0.6* | Red | Template preparation buffer |
| Template preparation buffer | 1 | 1.5 | Yellow | n/a |
| Water RNase/DNase Free | 1 | 1.5 | Clear or white | n/a |
| **Pack 3** | | | | |
| Genesig Easy RNA Internal extraction control (IEC) | 16 | 2.62 | Green | n/a |

** The projected volume once resuspended*

One kit of COVID-19 RT qPCR assay (HT-CE kit V2.0) kit from PrimerDesign LTD will enable testing of **1536 samples** (4 x 384-well qPCR plates) if using a 20µl assay, or 3072 samples (8 x 384-well qPCR plates) if using a 10µl assay and contains the following:

The COVID-19 Primer & Probe Mix contains the primers and FAM labelled probe specific to SARS-CoV-2 and the primers and HEX labelled probe specific to the Genesig Easy RNA Internal control (IC).

| **Reagent label** | **Number of vials (1536/3072**  **tests)** | **Volume mL/vial** | **Lid colour** | **Resuspended with?** |
| --- | --- | --- | --- | --- |
| **Pack 1** | | | | |
| **4x Precision Tough mix** | 1 | 9.5 | Orange | N/A |

| **COVID-19 Primer & Probe Mix (including IC primer/probe mix)** | 1 | 3.8 | Green | N/A |
| --- | --- | --- | --- | --- |
| **Pack 2** | | | | |
| **Genesig COVID-19 Positive control template** | 1 | 0.6* | Red or pink | Template preparation buffer |
| **Template preparation buffer** | 1 | 1.5 | Yellow | N/A |
| **Water (RNase/DNase free)** | 1 | 1.5 | Clear or white | N/A |
| **Pack 3** | | | | |
| **Genesig COVID-19 Internal control (IC)**** | 16 | 2.62 | Green | N/A |

**Projected volume once resuspended*

*******Note:*** *tube is labelled Internal Extraction Control. In direct to PCR this reagent is an Internal control as extraction is not performed*

## Sorting kit components

- - - 1. Collect all RNA Internal Extraction Control silver packets (packet 3) and transfer to -20°C storage in sample prep areas, station 1a and 1b.
      2. Collect all COVID-19 Positive Control Template silver packets (packet 2) and transfer to -20°C storage in PCR station 3c.
      3. Collect all primer/probe silver packets (packet 1, also contains Master Mix) and transfer to -20°C storage in PCR station 3.
      4. Complete stock inventory detailing number of kits, Lot numbers as detailed on the label on the outer silver packet (not the individual inner vial) and storage location located in the inventory: Cambridge Coronavirus test Centre Teams/3.PCR/Files/Inventory Master Mix.

## Preparation of the RT-qPCR (HT-CE assay kit)

- - - 1. If the 2X RT-qPCR Master Mix is frozen, thaw at room temperature until almost defrosted (ice crystals remain) and then move onto ice to complete the defrosting. If the COVID-19 Primer & Probe Mix is frozen, thaw on ice and keep protected from light.
      2. Prepare the reaction mix: Ensure you are using reagents from the same lot number. In a BSC, using a P1000 pipette or ‘Stripette’, add the primer/probe mix (3.8 mL) to the Master Mix in the Master Mix tube. Replace the orange cap and invert gently to mix. The volume of liquid can also be halved with remaining un- mixed reagents kept on ice or in the fridge (+4°C).
      3. Keep Falcon on ice if not dispensing into qPCR plates immediately.
      4. Register the batch number for the RT-PCR Master Mix in the P:/qPCRReagents/DailyPCRReagentsSheet Excel file on the PC’s associated with the BSC’s at station 3a.

## Preparation of the COVID-19 RT qPCR assay (HT-CE kit V2.0)

- - - 1. Collect packet 1 from -20°C storage and place Master Mix (orange lid) and Primer/probe mix (green lid) on ice. Ensuring the primer/probe mix is **protected from light.**
         1. Alternatively, the COVID-19 Primer & Probe Mix can also be defrosted overnight at 4°C in preparation for use by the next shift.

c. Primer/probe mix and Master Mix can be stored at 4°C. The date and time of defrosting will be written on the packet which also stated the expiry date.

- - - 1. Prepare the reaction mix: In a BSC measure all reagents based on the volumes in the table below using a stripette or pipette as appropriate into a 50mL falcon tube. HT-CE V2.0 toughmix is more viscous that HT-CE V1.0 and care should be taken to ensure complete mixing by inverting or pipetting several times. Avoid bubble formation. Do not vortex.

3.


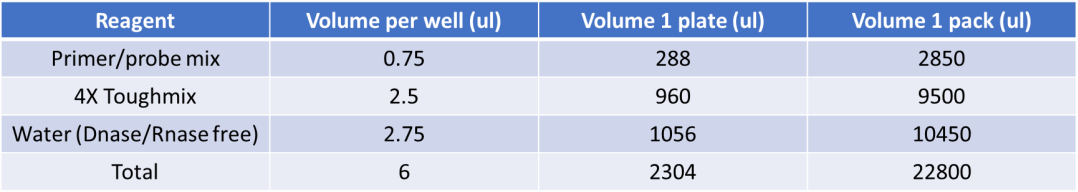


- - - - 1. If multiple packets are being used at once, combine each of the packets as described in step 2 before pooling the reaction mix in 1 x 50mL Falcon tube. Take care to only pool reagents of the **same lot number**. Falcon tubes should be labelled with Lot number, reconstitution date and the number of kits used.

**Note:** Do not add the primer/probe solution to the Master Mix until you are ready to use the reaction mix. If the components are being defrosted or stored at 4°C they should be kept as separate components.

- - - 1. Reconstituted reaction mix should be kept on ice for immediate use. Do not freeze thaw.

## Dispense of the RT-PCR mix into the 384-well PCR plate using MultiDrop

**At the beginning of the day, before first run:**

- - - 1. If not already in place, put and secure 8 tubes small cassette in a MultiDrop Combi machine located in a BSC. Check that the tubes are free of liquid.
      2. Switch on the MultiDrop Combi machine and set up the following parameters for the 20ul assay volume:
         1. 384-well Low Profile 10 mm plate
         2. Dispense 12ul using small cassette
         3. Dispense in Full plate (columns 1 to 24)
         4. Speed should be High (Medium speed for HT-CE V2.0 with z-axis height of 12mm)

Or for the 10ul assay volume:

- - - 1. Switch on the MultiDrop Combi machine and set up the following parameters for the 20ul assay volume:
         1. 384-well Low Profile 10 mm plate
         2. Dispense 6ul using small cassette
         3. Dispense in Full plate (columns 1 to 24)
         4. Speed should be High (Medium speed for HT-CE V2.0 with z-axis height of 12mm)
      2. For a half plate select columns rather than full plate on multidrop settings.
         1. Select all even numbered columns to fill quadrants Q1 and Q3. **This should be witnessed and settings returned back to full plate after.**
         2. Clearly label PCR plate as half plate and indicate which quadrants are filled.
      3. Clean the cassette by priming first with 70% Ethanol and then Rnase/Dnase- free water (around 25ml each). **Check that dispense tips are not blocked**.
      4. Run a test plate using Rnase/Dnase-free water to check head alignment and that dispensing performs correctly. To ensure correct catch, check that qPCR plate **position A1 is in the bottom right corner of the loader**.
      5. Empty tubes and release cassette. Cleaning of the multidrop cassettes is performed on the daily start up and directly after dispensing reaction mix.

**At each run:**

1. Remove the required number of qPCR plates from the packet in the BSC. Remove Roche plate barcode and cover with a PCR specific barcode starting ‘003’ on the north long side of the plate. Using the black marker pen also mark a black dot on the top left corner of the plate (near well A1) to orient the plate. Ensure that no other pen marks are on the plate or near any of the wells and the PCR plates are not misshapen.
2. Ensure that the multidrop tubes are free of liquid. If not, clean the head as described above.
3. Check that the dispense parameters are correct (see settings above).
4. Put the tubes at the bottom of the PCR Master Mix 50ml Falcon tube and prime the system. Check that liquid is dispensed from all 8 tips.
5. Once tubes are primed, put a 384-well PCR plate on the MultiDrop Combi machine and check that **position A1 is in the bottom right corner of the loader**. Dispense 12ul per well for 20ul protocol; dispense 6ul per well for 10ul protocol.
6. Repeat dispensing for the number of PCR plates to prepare. Ensure that enough volume of PCR Master Mix was used by checking that liquid is present in the tubes. Keep the qPCR plate(s) in the hood for NTC addition (see below).
7. After the run, wash the system first with 70% Ethanol and then with Rnase/Dnase-Free water (~ 25ml each).
8. Finally, prime extensively (~ 30 seconds) with air to dry, release the cassette and put the extremity of the tubes into an empty Falcon in the BSC.

## Add Rnase/Dnase-Free water to No Template Control wells

- - - 1. Collect one vial per qPCR plate of Rnase/Dnase Free water from the COVID-19 Genesig Real-Time PCR assay kit stored in the RT-PCR preparation room.
      2. Using either a single or multi dispense pipette: For the 20ul assay, dispense 8ul of Rnase/Dnase-Free water; or for the 10ul assay, dispense 4ul into the No Template Control wells of each 384-well PCR plates, corresponding to wells **C1, C2, D1 and D2** (see plate map below). Ensure a tip change per vial of water (per plate).
      3. Apply a non-PCR plate seal to the prepared qPCR plate.

**A WITNESS IS REQUIRED TO CHECK THE CORRECT ADDITION OF WATER INTO NTC WELLS**

|  | 1 | 2 | 3 | 4 | 5 | 6 | 7 | 8 | 9 | 10 | 11 | 12 | 13 | 14 | 15 | 16 | 17 | 18 | 19 | 20 | 21 | 22 | 23 | 24 |  |  |
| --- | --- | --- | --- | --- | --- | --- | --- | --- | --- | --- | --- | --- | --- | --- | --- | --- | --- | --- | --- | --- | --- | --- | --- | --- | --- | --- |
| A |  |  |  |  |  |  |  |  |  |  |  |  |  |  |  |  |  |  |  |  |  |  |  |  |  | **PCT** |
| B |  |  |  |  |  |  |  |  |  |  |  |  |  |  |  |  |  |  |  |  |  |  |  |  |  |  |
| C |  |  |  |  |  |  |  |  |  |  |  |  |  |  |  |  |  |  |  |  |  |  |  |  |  | **NTC** |
| D |  |  |  |  |  |  |  |  |  |  |  |  |  |  |  |  |  |  |  |  |  |  |  |  |  |  |
| E |  |  |  |  |  |  |  |  |  |  |  |  |  |  |  |  |  |  |  |  |  |  |  |  |  | **NEC** |
| F |  |  |  |  |  |  |  |  |  |  |  |  |  |  |  |  |  |  |  |  |  |  |  |  |  |  |
| G |  |  |  |  |  |  |  |  |  |  |  |  |  |  |  |  |  |  |  |  |  |  |  |  |  | **Samples** |
| H |  |  |  |  |  |  |  |  |  |  |  |  |  |  |  |  |  |  |  |  |  |  |  |  |  |  |
| I |  |  |  |  |  |  |  |  |  |  |  |  |  |  |  |  |  |  |  |  |  |  |  |  |  |  |
| J |  |  |  |  |  |  |  |  |  |  |  |  |  |  |  |  |  |  |  |  |  |  |  |  |  |  |
| K |  |  |  |  |  |  |  |  |  |  |  |  |  |  |  |  |  |  |  |  |  |  |  |  |  |  |
| L |  |  |  |  |  |  |  |  |  |  |  |  |  |  |  |  |  |  |  |  |  |  |  |  |  |  |
| M |  |  |  |  |  |  |  |  |  |  |  |  |  |  |  |  |  |  |  |  |  |  |  |  |  |  |
| N |  |  |  |  |  |  |  |  |  |  |  |  |  |  |  |  |  |  |  |  |  |  |  |  |  |  |
| O |  |  |  |  |  |  |  |  |  |  |  |  |  |  |  |  |  |  |  |  |  |  |  |  |  |  |
| P |  |  |  |  |  |  |  |  |  |  |  |  |  |  |  |  |  |  |  |  |  |  |  |  |  |  |

- - - 1. Discard Rnase/Dnase-Free water tube.
      2. Clean the BSC working area with Distel then with 70% Ethanol spray.

## PCR plate processing and storage

- - - 1. After adding the water to the NTC wells, seal PCR plates with e.g. Perkin Elmer Clear Adhesive Film. Plates can now be moved out of the BSC.
      2. For each prepared qPCR plate, record on the plate seal to confirm the addition of the NTC, the date and time of preparation.
      3. Open P:/MultidropHeadScanningSheet_V2 excel spreadsheet on the PC associated with the BSC at station 3a and for each plate record the qPCR plate barcode and the multidrop head used by scanning the barcode for that head.
      4. If not used immediately, the prepared qPCR plates can be stored at **+4°C in the dark up to 24 hours for HT-CE assay kits and 12 hours for HT-CE V2.0 assay kits. Where possible store thawed reagents at +4°C prior to mixing or plating.**

## Pre-automation procedure

- - - 1. Collect RNA samples source plates (96-well format) in the Station 3 RNA Input Fridge. Centrifuge at 1000rpm (Thermo Scientific Megafuge with swing out rotor TX1000) and at 9oC to pull liquid to the bottom of the wells. Scan plates into PCR tracking sheet.
      2. Collect 1 x RT-qPCR plate (384-well format) containing Master Mix and

NTC from station 3 PCR preparation fridge, The qPCR plate may need a short centrifugation if there is condensation on the plate seal – 1200 rpm for

1min. Scan plate into PCR tracking sheet (PCR tracking sheet can be found on the CBCTC – Internal, PCR Prep conversation area on Microsoft Teams).

- - - 1. **Remove tips in positions A1 and B1**, corresponding to positive and negative control wells, for 4 boxes (or less depending on the number of source plates to process) of 96 Sterile Filtered tips for use on the Bravo and dispose of them in a sealable Bio-bin. If any specific wells need to be avoided (e.g. because of sample viscosity/heavy beads from RNA extraction (see Appendix figure 9)) the corresponding tips for these wells are also removed from the tip box at this stage. Removal of these tips is always witnessed and double checked by a second scientist. Ensure that the remainder of the box contains tips and that they are seated correctly in the box. Make sure the Shift Lead is aware that this well needs to be voided on LIMs.

## Automation Procedure

The plate to plate transfer of the extracted RNA samples in 96-well plates into the 384-well RT-qPCR plate will be done using the Agilent Bravo fitted with a 96LT head. For the 20ul Assay, 8ul RNA is transferred into the RT- qPCR plate; and for the 10ul Assay, 4ul RNA is transferred.

**Before use, clean the Bravo deck and working area around the robot with Distell and 70% Ethanol and close the VWorks software if open**

- - - 1. Open Bravo VWorks software and login
      2. Open the correct protocol from the Desktop Folder:
      3. If prompted to initialize the device say “Yes”
      4. The “MANUAL LOADING INSTRUCTIONS” form should be displayed
      5. Load the robot to match the figure below:


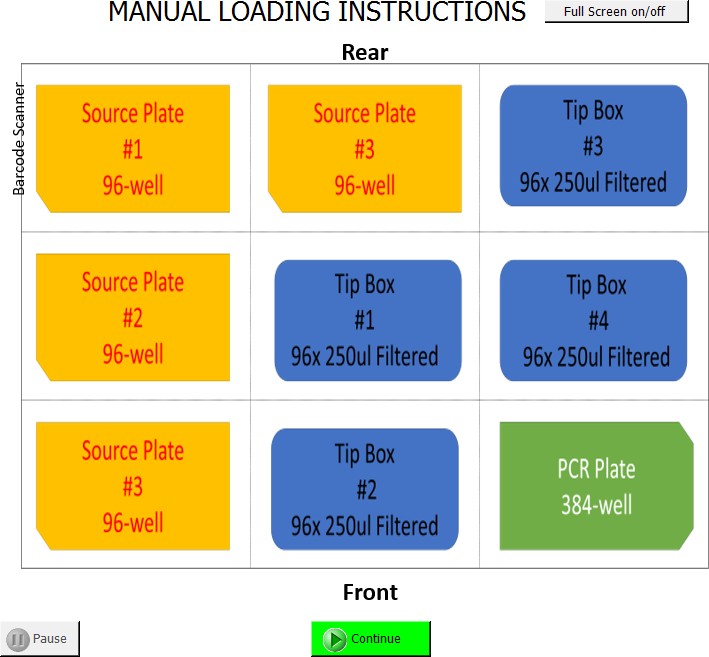


- - - 1. If x4 source plates are not required load a “DUMMY PLATE” onto empty positions. Treat dummy plates as normal source plates for rest of the method.
      2. When labware is loaded click the “Continue” button on the form
      3. Run configuration wizard will appear. Leave as defaults and click “Finish”
      4. Loading Confirmation Box will appear. Follow steps and click “Continue”
      5. Scan in the barcodes as prompted
      6. Check each barcode with your lab partner before continuing: Click “OK”
      7. If plate barcodes have been scanned more than once an error message will appear. Abort run and re-start protocol
      8. Enter the lead user ID: Click “OK”

**A WITNESS IS REQUIRED TO CHECK THE BARCODE SCANNIN AND FINAL LAYOUT BEFORE THE RUN**

**CHECK ALL PLATES AND TIPS ARE ORIENTATED WITH A1 POSITIONED TO THE TOP LEFT OF THE SILVER PLATE PADS,**

**CHECK TIP BOXES ARE DELIDDED AND TIPS REMOVED FROM A1 & B1, CHECK SEALS HAVE BEEN REMOVED FROM ALL PLATES**

- - - 1. Enter the checking user ID: Click “OK”
      2. The system will now run

## Post Automation Procedure

- - - 1. **Loosely apply optical grade LightCycler seal** on the 384-well RT-qPCR plate and transfer it to station 4a BSC in the RT-PCR analysis room for addition of the positive control template (see SOP CB 06 Station 3&4: Preparation and addition of Positive Control to PCR plate. Running of RT-qPCR and data export).
      2. Seal used source plates, clearly mark as used and the date and store them in the RNA output fridge in the annexe of PCR prep room (station 3).
      3. Remove tip boxes from the Bravo deck and re-lid before disposal.
      4. Clean the Bravo deck and working area with 70% ethanol on lint free tissue or Azowipes. Wipe Dry.

## Waste Management

- Dispose of all tips and tubes in the Bio-bin located in the BSC.
- Seal the Bio-bins and place in the autoclave bags outside the BSC

1. **RESPONSIBILITIES**
   - It is the responsibility of all personnel handling samples to follow this procedure.
   - It is the responsibility of personnel performing this procedure to ensure all equipment, chemicals, reagents, and solutions are properly labelled and that proper documentation is maintained.
   - It is the responsibility of the laboratory lead to ensure that this procedure is followed and that it is updated as necessary.

End of SOP CB 05 Version5.2

**SOP CB 06: STATION 3&4 PREPARATION AND ADDITION OF POSITIVE CONTROL TO PCR PLATE, RT-QPCR RUNNING AND DATA EXPORT (Version4)**

1. **PURPOSE**

The Cambridge COVID-19 Testing Centre (CCTC) based at the Anne McLaren Building is one of the National Testing Centres for COVID-19. The purpose of this facility is to test for the presence the SARS-CoV-2 virus. This procedure provides the method for preparing the Positive Control Template, dispensing it into

RT-qPCR plate, running the RT-qPCR plate and exporting the data.

1. **AUDIENCE**

This SOP applies to all on site trained personnel who are responsible for assessing samples for COVID-19 screening.

1. **SCOPE**

This document defines the required procedure for Station 3&4 for preparing the Primerdesign Ltd COVID-19 Genesig Real-Time PCR assay Positive Control Template and adding it into the PCT wells of the 384-well RT-qPCR plate. Performing the analysis run and interpreting the data with the purpose of detecting the presence of nucleic acid from SARS-CoV-2 in oropharyngeal and nasopharyngeal swabs. The assay can be run as a 20ul or 10ul total assay volume in a 384-well RT-qPCR plate.

1. **GLOSSARY**

| **Terms** | **Definition** |
| --- | --- |
| SARS-CoV-2 | The virus that causes COVID-19 |
| COVID-19 | Coronavirus disease 2019 |
| DNA | Deoxyribonucleic Acid |
| RNA | Ribonucleic Acid |
| PCR | Polymerase Chain Reaction |
| RT-PCR | Reverse Transcription - Polymerase Chain Reaction |
| PPE | Personal Protective Equipment |
| BSC | Biological Safety Cabinet |
| PCT | Positive Control Template |
| NTC | No Template Control |
| NEC | Negative Extraction Control |
| IEC | Internal Extraction Control |
| min | minute |

1. **SAFETY OVERVIEW**

# Hazards

| **Reagent** | **Hazard** | **Hazard Statement(s)** |
| --- | --- | --- |
| **Primerdesign Ltd COVID-19 Genesig Real-Time PCR**  **assay** | Non-hazardous |  |
| **70% Ethanol** | 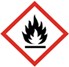 | H225, H319 |
| **Distel (1:10)** | Non-hazardous |  |

- 1. **Personal Protective Equipment**
     - Fully buttoned-up Howie-style laboratory coat
     - Standard nitrile gloves
     - Laboratory safety glasses or over-glasses

1. **PROCEDURE**

# General considerations

## Warnings and precautions

- - - - Treat all specimens as a biohazard.
      - Refer to the task-based risk assessment.
      - Discard all gloves, pipette tips, pipettes, vials, test tubes, or other disposable items into appropriately labelled sharps biohazard boxes or autoclave bags.
      - Use separated working areas for specimen preparation, reaction set up and amplification, with separate supplies and equipment.

## Preventing Contamination

- - - - Incorrect results could occur if either the clinical specimen or the real-time PCR reagents used in the amplification step become contaminated by accidental

introduction of amplification product (amplicon) or positive control reference material.

## The Genesig COVID-19 positive control template is provided in a sealed foil envelope and contains a high copy number of templates. It should be opened and processed away from test samples and kit components to avoid cross-contamination.

- - - - When handling/pipetting the positive control template wear double gloves – the outer pair is to be removed immediately after the pipetting procedure. Change all gloves between samples and whenever contamination is suspected.
      - Only disposable/paper lab coats are to be worn in station 3c (PCT room) and station 4 (RT-PCR analysis) which are to be disposed of at the end of the weekly shift.
      - Keep reagent and reaction tubes capped or covered as much as possible
      - Always check the expiration date prior to use. Do not use expired reagent. Do not substitute or mix reagent from different kit lots.
      - Work surfaces, Biological Safety Cabinet, pipettes and centrifuges should be cleaned and decontaminated with cleaning products (e.g. DNA/RNA remover, 70% ethanol, Distel 1:10) to minimize risk of nucleic acid contamination.
      - Use UV lighting in the BSC’s used for handling/dispensing Master Mix and PCT to decontaminate cabinets in the case that contaminated plates are identified during the data analysis or a spill of the PCT occurs within the BSC.
      - Change gloves whenever contamination is suspected and when changing work station.
      - Reagents and equipment should not be moved from a dirty area to a clean area. Should a case arise where a reagent or piece of equipment needs to be moved backwards, it must first be decontaminated with Distel and wiped down with 70% ethanol.

## Preventing DNase/RNAse contamination

- - - - Use DNase/RNase free disposable plasticware and pipettes reserved for DNA/RNA work to prevent cross-contamination with DNases/RNases from shared equipment.
      - Use DNase/RNase free filter tips throughout procedure to prevent aerosol and liquid contamination.
      - Wipe surfaces of workbenches and biological safety cabinets with RNAse away followed by 70% ethanol.
      - Where RNA samples are not able to be processed as soon as possible for example in the event of Bravo failure, maximum capacity reached, awaiting further RNA plates for processing onto the same PCR plate or during shift handovers, the sealed RNA plates may be stored at 4^0^C in a refrigerator for a maximum of 12 hours, prior to RT-qPCR set up. Storage will be in the ‘RNA Input’ fridge in PCR-Prep (station 3b), the plates will be fully sealed and the time of preparation clearly written on the seal.

# Procedure Schematic

Collect Genesig COVID-19 Positive control template and template preparation buffer vials


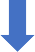


Resuspend lyophilised COVID-19 Positive control template at 1.7x10^5^ copies / ul in a dedicated room


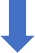


Transfer positive control template to station 4a BSC/PCR hood and keep vial on ice Add positive control template to PCT wells of the RT-qPCR plate


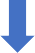

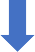


Firmly seal PCR plate and centrifuge plate to remove any air bubbles Generate qPCR file in LIMfinity using LightCycler laptop


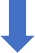

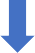


Open LightCycler software and populate run details Select destination for result file and run qPCR


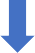

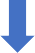


Export result .ixo file to L:/ drive for data analysis and archive .ixo file on LightCycler laptop

# Work Station Schematic


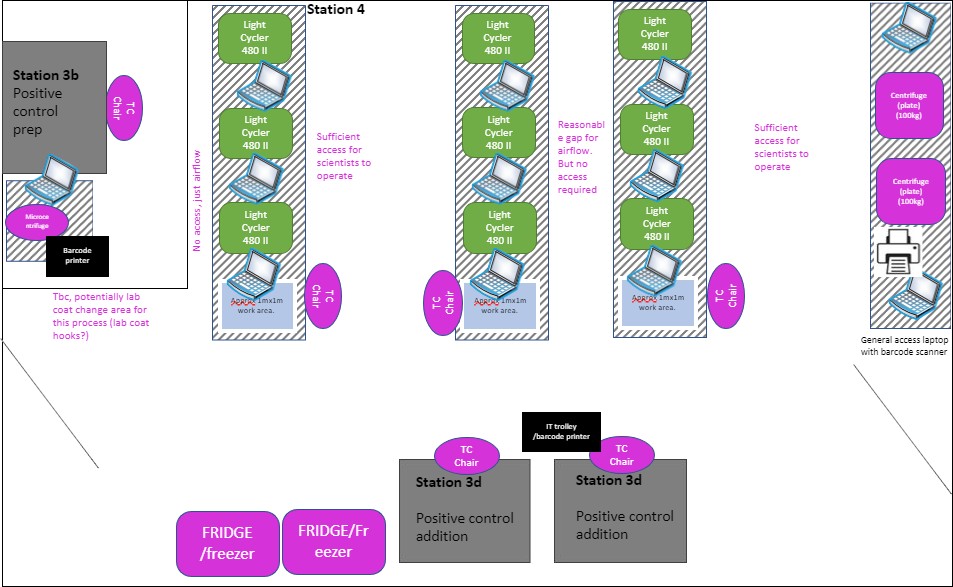


**Station 4a**

**Station 4a**

**Station 3c**

For reference, a typical layout of a Station 4 RT-PCR Analysis Laboratory

# Materials and Reagents

| **Reagent / Labware** | **Comment** |
| --- | --- |
| Genesig COVID-19 Positive control template (Lyophilised) | From PrimerDesign LTD COVID-19 RT qPCR assay kit |
| Template preparation buffer | From PrimerDesign LTD COVID-19 RT  qPCR assay kit |
| P1000 tips, sterile, filtered |  |
| P200 tips, sterile, filtered |  |
| Distel (1:10) |  |
| Ethanol 70% spray |  |

| **Equipment** | **Comment** |
| --- | --- |
| BSC hoods dedicated to the preparation and  addition of positive control template |  |
| Fridge (+4°C) |  |
| Freezer (-20°C) |  |
| Electronic single channel Multidispense pipette  P200 + carousel charging stand |  |
| Single channel pipette P1000 |  |
| Vortex |  |

| Benchtop centrifuge for microtubes |  |
| --- | --- |
| Waste container for tips and tubes | Biobin |
| Ice box containing ice |  |
| Secondary container for microtubes |  |

- 1. **General guidelines**
     - Perform all steps on ice (+4^o^C), unless otherwise noted
     - Perform all steps in a BSC to avoid contamination unless otherwise noted
     - Perform all steps wearing appropriate PPE
     - Avoid creating bubbles during mixing and aliquoting

# Procedure

- - - The Primerdesign Ltd COVID-19 Genesig Real-Time PCR assay is shipped at ambient temperatures but must be stored at -20°C upon arrival.
    - Always check the expiration date prior to use. Do not use expired reagents.
    - Protect fluorogenic primer/probe mix from light.
    - On receipt, Positive control template can be stored at -20ºC for up to six months or the expiry date, whichever occurs first.
    - A fresh stock of PCT is prepared on the morning of each working day and throughout the day as required and stored on ice in the dedicated BSC in station 4a. At the end of each day this stock is disposed of and not stored for re- use.
    - The RT-qPCR analysis will be done using a Roche LightCycler 480 instrument
    - Before use, clean the working area around the instrument with Distel and 70% Ethanol
    1. **Positive Control Template components**

Collect the lyophilised COVID-19 Positive control template from the PrimerDesign LTD COVID-19 RT qPCR assay kit **(red lid vial stored in sealed silver foil pouch)** and store at -20ºC in the positive control preparation room.

Template preparation buffer can also be stored in the positive control preparation room at -20ºC.

- - 1. **Reconstitution and dilution of the Positive Control Template**

**Work to be performed in a dedicated BSC in the positive control preparation room. The user handling this template should wear a separate lab coat and 2 pairs of gloves**

- - - 1. Collect the lyophilised Positive Control Template tube (red lid) in the dedicated fridge/freezer in the Positive Control room and pulse spin briefly to pull dried reagent to the bottom of the tubes.
      2. Record the PCT batch number and message to the PCR preparation lab (station 3a) to register it P:/qPCRReagents/ excel file that is accessed from PC’s in station 3a and 3b.
      3. Resuspend the lyophilised Positive Control Template with **600uL of Template preparation buffer**, mix gently and perform a quick spin to pull liquid to the bottom of the tubes. This will generate a PCT concentration of 1.7 × 10^5^ copies/uL.
      4. Put the PCT vial into a secondary container filled with ice and transfer out of the positive control preparation room into an ice bucket inside a dedicated BSC in the main laboratory (Station 4a, see map above).
      5. Clean all surfaces, pipettes and tip racks in the positive control preparation room BSC with Distel and 70% ethanol.
    1. **Add Positive Control Template to PCT wells of the 384well PCR plate**

**Work to be performed in a dedicated BSC in the RT-PCR Analysis room. The user handling this template should wear 2 pairs of gloves**

- - - 1. Following the addition of the RNA samples, the sealed 384-well RT-qPCR plate will be transported from station 3b (PCR prep) to station 4 (RT-qPCR). The PCR prep scientist from station 3b will not enter station 4.
      2. The witness scientist in station 4 collects the sealed 384-well RT-qPCR plate from the spill tray and transfers it to the dedicated BSC for positive control addition at station 4a in the RT-PCR Analysis room.
      3. The witness scientist will carefully peel off the top-left corner of the PCR plate corresponding to wells A1 to B2 and holds this in place whilst the second scientist, carefully dispenses Positive Control Template into the PCT wells **A1, A2, B1 and B2** of the 384-well PCR plate (see plate map below).
         1. 8 ul/well of PCT is used of the 20ul assay
         2. 4 ul/well of PCT is used for the 10ul assay

**Take care to not contaminate the adjacent wells with the Positive Control Template**. When completed, the witness scientist puts the plate sealer back in place and seals it firmly using a seal applicator or roller. Plate can now be safely moved out of the BSC.

- - - 1. The plate is then carried to the benchtop centrifuge in (after removal of the outer pair of gloves) and is centrifuged in a sealed bucket, for 1min at 1200rpm (142xg on thermo Megafuge 16R, M-20 rotor) to remove any air bubbles.

## A WITNESS IS REQUIRED TO CHECK THE ADDITION OF PCT INTO THE CORRECT WELLS

|  | 1 | 2 | 3 | 4 | 5 | 6 | 7 | 8 | 9 | 10 | 11 | 12 | 13 | 14 | 15 | 16 | 17 | 18 | 19 | 20 | 21 | 22 | 23 | 24 |  |  |
| --- | --- | --- | --- | --- | --- | --- | --- | --- | --- | --- | --- | --- | --- | --- | --- | --- | --- | --- | --- | --- | --- | --- | --- | --- | --- | --- |
| A |  |  |  |  |  |  |  |  |  |  |  |  |  |  |  |  |  |  |  |  |  |  |  |  |  | **PCT** |
| B |  |  |  |  |  |  |  |  |  |  |  |  |  |  |  |  |  |  |  |  |  |  |  |  |  |  |
| C |  |  |  |  |  |  |  |  |  |  |  |  |  |  |  |  |  |  |  |  |  |  |  |  |  | **NTC** |
| D |  |  |  |  |  |  |  |  |  |  |  |  |  |  |  |  |  |  |  |  |  |  |  |  |  |  |
| E |  |  |  |  |  |  |  |  |  |  |  |  |  |  |  |  |  |  |  |  |  |  |  |  |  | **NEC** |
| F |  |  |  |  |  |  |  |  |  |  |  |  |  |  |  |  |  |  |  |  |  |  |  |  |  |  |
| G |  |  |  |  |  |  |  |  |  |  |  |  |  |  |  |  |  |  |  |  |  |  |  |  |  | **Samples** |
| H |  |  |  |  |  |  |  |  |  |  |  |  |  |  |  |  |  |  |  |  |  |  |  |  |  |  |
| I |  |  |  |  |  |  |  |  |  |  |  |  |  |  |  |  |  |  |  |  |  |  |  |  |  |  |
| J |  |  |  |  |  |  |  |  |  |  |  |  |  |  |  |  |  |  |  |  |  |  |  |  |  |  |
| K |  |  |  |  |  |  |  |  |  |  |  |  |  |  |  |  |  |  |  |  |  |  |  |  |  |  |
| L |  |  |  |  |  |  |  |  |  |  |  |  |  |  |  |  |  |  |  |  |  |  |  |  |  |  |
| M |  |  |  |  |  |  |  |  |  |  |  |  |  |  |  |  |  |  |  |  |  |  |  |  |  |  |
| N |  |  |  |  |  |  |  |  |  |  |  |  |  |  |  |  |  |  |  |  |  |  |  |  |  |  |
| O |  |  |  |  |  |  |  |  |  |  |  |  |  |  |  |  |  |  |  |  |  |  |  |  |  |  |
| P |  |  |  |  |  |  |  |  |  |  |  |  |  |  |  |  |  |  |  |  |  |  |  |  |  |  |

1. Clean the BSC working area with Distel then with 70% Ethanol.
2. The vial of reconstituted Positive Control Template can be kept on ice in the dedicated BSC but must be discarded at the end of the day (single use).
   - 1. **Prepare the RT-qPCR plate for the analysis run**
        1. Collect the sealed 384 well RT-qPCR from BSC at station 4a after the addition of the positive control template.
        2. Firmly affix the seal on the PCR plate using a seal applicator or roller, paying extra attention to edges and remove the white edge of the seal.
        3. Centrifuge RT-qPCR plate in a sealed bucket, for 1min at 1200rpm (142xg on thermo Megafuge 16R, M-20 rotor) at room temperature to remove any air bubbles.


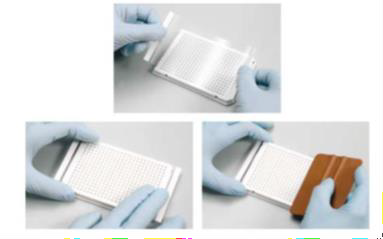


*Figure 1. How to seal PCR plate.*

- - - 1. Perform final checks on the plate before the run. Inspect the seal for creases and that each well is properly sealed. Inspect the plate for bubbles and for liquid level. Ensure there are no pen marks/writing on the plate or sticky lid, other than the dot denoting the position of well A1, and lines obscuring the underlying barcode on the north face of the plate.
    1. **Run RT-qPCR analysis**

**Follow instructions below on how to run the instrument:**

- - - 1. If not already, open the ‘PCRPlateTool’ app (icon on the desktop)
      2. Login to the 'Live system' Environment using your LIMfinity username and password. (If performing validation login to the UAT environment)

1. Use the barcode reader to scan the barcode of the prepared PCR plate into the 'Plate ID' field
2. Check the platemap of the plate along with the details in the right-hand side panel. Pay particular attention to the number of samples
3. In the **File** menu select **Generate PCR File**
4. A dialog will be shown confirming the PCR plate map has been generated and path copied to clipboard. Press **OK**
   - - 1. If not already, open LightCycler 480 SW software (icon on Desktop) and log in using specific credentials for that instrument (speak to Lab Leader for details)
5. Select Run protocol
6. Select ‘New experiment from template’
7. Select: Covid-19_qPCR_20ul_Protocol for the 20ul assay or Covid- 19_qPCR_10ul_Protocol for the lower volume 10ul assay
   - - 1. Apply subset template to distinguish between the 4 x 96 well quadrants
8. Go to ‘Subset Editor’ then “Apply template”
9. Select: Covid/Covid-19_Subset_Template
   - - 1. Add sample plate map generated from PCRPlateTool App
10. Go to ‘Sample Editor’ window and -press 'Import'
11. Paste the path to the platemap which you copied to clipboard earlier (step 2d), confirm plate ID in the filename
12. Click 'Scan File' & Accept
    - - 1. Go back to the ‘Experiment’ window, and load plate with well A1 in top left position. Close loader and **copy qPCR plate barcode which is now available in the ‘plate ID’ field of the software**
        2. Start Run and save .ixo file (LightCycler run file) in **Covid/Results** folder using the following naming convention: **“Plate barcode_LC#_initials_shift#”**. The plate barcode is pasted from step 6. Ensure Lightcycler protocol starts successfully
        3. After successful completion of the LightCycler run (approx. 1hr 25mins), close the experiment by clicking the **X** button on the right-hand menu
        4. Click the **Navigator** (compass) button and select the finished experiment in the Results folder
        5. Click: "**Export**" **and Save** the result into 'IXO files' folder (Found in 'Quick Links' or C:/CBCTC/PCRPlateTool/IXOFiles)
        6. Move .ixo file to the Results Archive folder (**Covid\Results**\Archive) found in the LightCycler software on the C: drive of the LightCycler laptop
        7. Go back to PcrPlateTool app. If not already open with the correct plate, eject and scan PCR plate barcode as in step 2a
        8. In the **File** menu, select **Process IXO File**
        9. This will perform checks within LIMs, push the IXO file to the data team and archive the PCR plate in LIMFinity
        10. Wait for confirmation that all is OK
        11. Remove plate from instrument
      1. **Waste Management**

- Dispose all tips and tubes in the 2L Bio-bin in the BSC.
- Seal the Bio-bins and place in the autoclave bags outside the BSC
  - 1. **Acceptance of a run**

In the following circumstances the qPCR plate may be recreated from the original RNA plates

- If the PCR plate was dropped during the preparation process
- If a reagent or positive control was not added during the PCR plate preparation or dispensed into the incorrect well
- If there was evidence that the PCR plate has been contaminated during preparation
- If the Bravo has a technical failure during the stamping out of RNA plates
- If an extra drop of liquid/increased volume is dispensed from the Bravo tips due to significant bead carryover
- In the event of instrument failure e.g. LightCycler not completing all of the cycles

The following actions need to be taken

- Report to Lab Lead & STOP plate immediately. If the LightCycler run has not started then DO NOT create the .ixo file. If the LightCycler run is

in progress then abort the run and do not export the .ixo file

- Return to station 3
- Cancel PCR plate in LIMfinity and include a comment to document the reason for cancellation
- Re-use the RNA plates and a new qPCR plate containing Master Mix to create a new qPCR plate with new barcode
- Always ensure Bravo has successfully executed before uploading to LIMfinity
- Register the new PCR plate using the new Bravo file (see SOP CB 05: station 3 use and storage of HT kit reagents for preparation of 384-well PCR plate and addition of negative control and RNA template.)
- Run new plate on LightCycler steps 6.6.3 to 6.6.5.

1. **RESPONSIBILITIES**

- It is the responsibility of all personnel handling samples to follow this procedure.
- It is the responsibility of personnel performing this procedure to ensure all equipment, chemicals, reagents, and solutions are properly labelled and that proper documentation is maintained.
- It is the responsibility of the laboratory lead to ensure that this procedure is followed and that it is updated as necessary.

End of SOP CB 06 Version4

**SOP CB 17 STATION 5A: RT-QPCR DATA ANALYSIS (Version6)**

1. **PURPOSE**

The Cambridge COVID-19 Testing Centre (CCTC) based at the Anne McLaren Building is one of the National Testing Centres for COVID-19. The purpose of this facility is to test for the presence of the SARS-CoV-2 virus. This procedure provides the method for uploading the RT-PCR data to the analysis software, the quality control/analysis itself and its interpretation.

1. **AUDIENCE**

This SOP applies to all on site trained personnel who are responsible for assessing samples for COVID-19 screening.

1. **SCOPE**

This document defines the required procedure for Station 5 for the RT-qPCR quality control/analysis and interpreting the data with the purpose of detecting the presence of nucleic acid from SARS-Cov-2 in oropharyngeal and nasopharyngeal swabs.

St at ion 0: Sample receipt & initial unpacking St at ion 1: Sample preparation

+

+

St at ion 2: RNA extraction by Beckman iS/ i7 Station 3: PCR preparation

+

+

St at ion 4: RT-PCR analysis


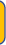


Station Sa: QC of RT-PCR data

St at ion Sb : Report of results

+

1. **GLOSSARY**

| **Terms** | **Definition** |
| --- | --- |
| SARS-CoV-2 | The virus that causes COVID-19 |
| COVID-19 | Coronavirus disease 2019 |
| RNA | Ribonucleic Acid |
| PCR | Polymerase Chain Reaction |
| RT-PCR | Real Time - Polymerase Chain Reaction |
| PCT | Positive Control Template |
| NTC | No Template Control |
| NEC | Negative Extraction Control |
| IEC | Internal Extraction Control |
| Ct / Cq | Ct means cycle threshold, Cq quantification cycle, they are interchangeable. |
| QC | Quality control |

1. **SAFETY OVERVIEW**

# Hazards

No hazardous materials used

# Personal Protective Equipment

No protective equipment needed

1. **PROCEDURE**

# General considerations

## Hardware requirements:

- - - - Processor: 2 GHz, 2 GB RAM
      - Disk space: 10 GB
      - Internet Connection Cable or DSL
      - Screen resolution: min. 1366 x 768 pixels, max. 1920 x 1080 pixels
    - **Operating system:** Windows 7 or higher

## Supported browsers:

- - - - Internet Explorer 11 or newer
      - Microsoft Edge 25 or newer
      - Firefox 45 or newer
      - Google Chrome 47 or newer

# User Levels and new user addition

To add new users to Fastfinder the DSM should contact the change control officer or the BSO (Data). User access is assigned based on job as per the table below.


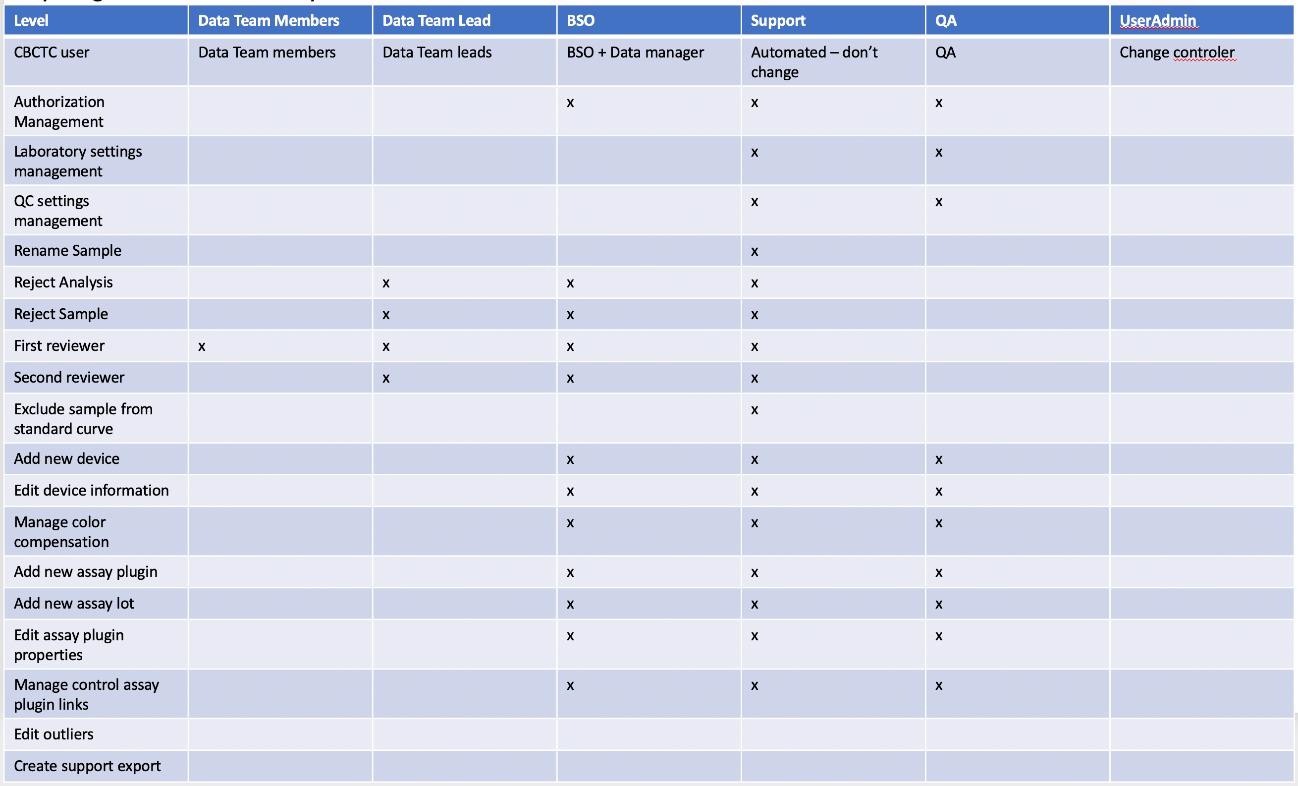


Before starting on the data team a new team member will have training on Fast Finder, with Ugentec and the Data BSO. They will then receive on-shift training from their Data Team Lead.

- 1. **Procedure Schematic**


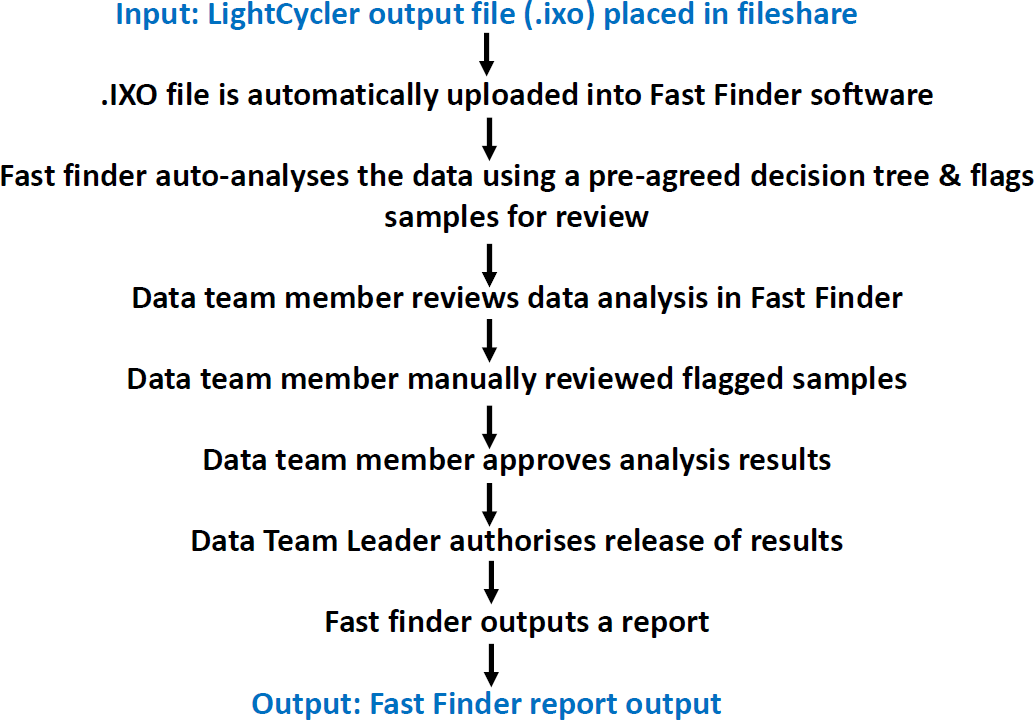


- 1. **Materials and Reagents**

| **Equipment** | **Comment** |
| --- | --- |
| Asus laptop X509JA-EJ031T |  |
| Software UgenTec Fastfinder v3.300.4 |  |

- 1. **General guidelines**
- The data scientist checks regularly for incoming data during the shift
- There will be 2- step validation of all data with the first and second approver both checking the plate before final approval of the data.
  - First approval can be done by anyone on the data team.
  - Second approval can only be done by persons who have received extra training and as authorised by the BSO. In addition, the second approver cannot be the same person as the first approver.
- Data analysis will be split into subsets in the UgenTec software, allowing the four 96 well plates on one 384 well plate to be matched to their original controls, e.g. If controls fail for one 96 well plate, only the samples on that original 96 well plate will be affected as per the decision tree.
- QC criteria are pre-set in UgenTec software and can be viewed through “Analysis” 🡪 plugin name. This is included in the decision tree in the appendix to this SOP

# Procedure

- The QC of RT-qPCR data will be completed using the software UgenTec FastFinder
- Login to UgenTec FastFinder software
- Check under “Start” for new data to be assessed under “in progress/to be reviewed”
  - Select data of interest
- 1^st^ Approver assesses analysis results. All the steps below must be carried out.
  - On the overview tab check the top grey box. If there are no identified issues on the plate all 3 will have green ticks. If not, then a red cross will be present.


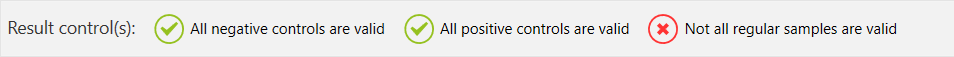


- - First select the resolve tab. Here any results Fastfinder has marked for review are highlighted.
  - Within each sample the curve to review will be highlighted by a tick mark.


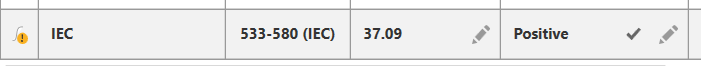


- - If the user agrees with the interpretation press the tick mark, if not the result for the curve is changed using the pencil button and then the tick pressed. If a change is performed then a comments box will appear. This forms the audit trail. In the case of changing a sample from negative to inconclusive the user will be required to enter a Cq value. In this case a value of 45 is used.


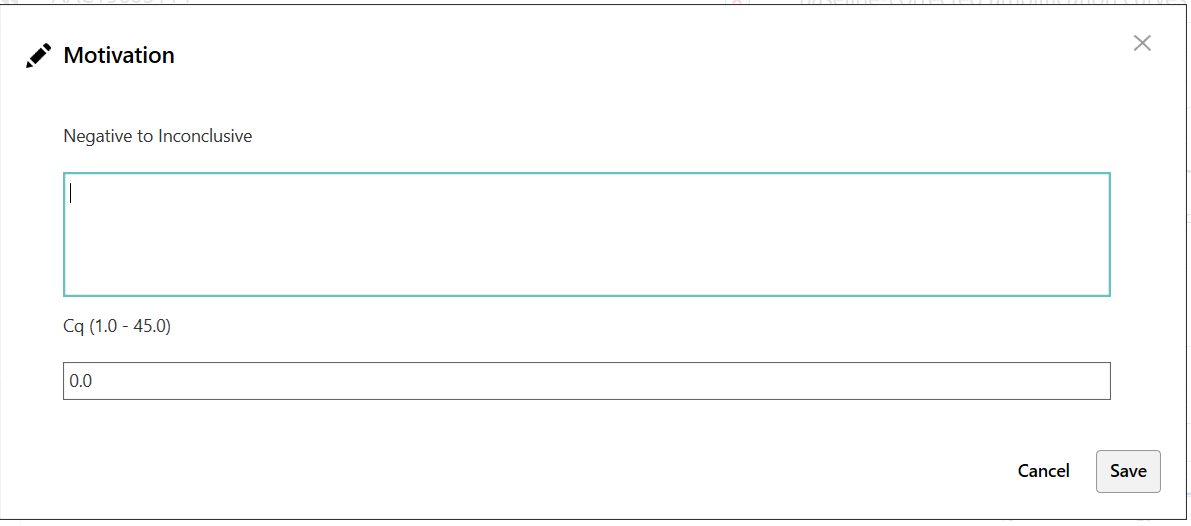


- - Note if the user is unsure of any curve for a sample it is always changed to inconclusive and never to positive or negative.
  - Note at no point (apart from changing a negative to an inconclusive and setting the Cq to 45) must the reviewer estimate or alter the Cq value.
  - Once all highlighted samples have been resolved move on to the Well details tab.
  - In the well details tab the user must look at the plate heatmap for any obvious contamination patterns, patterns in the results or excessive numbers of voids. If there is an issue follow the guidance in the production guidance (Found on Teams in CBCTC internal – General, this document is maintained by the BSOs). The colours in the heatmap are as follows
    - Green – Negative sample
    - Pink – Weak positive sample
    - Red – Positive sample
    - Black – Strong positive sample
    - Yellow – invalid well
  - Next the user clicks on the Target details tab. The user then clicks on “Select all positives” and “Select all negatives” in turn.


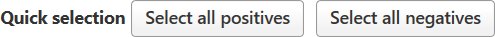


This overlays all the positives and negatives on the PCR plate. If there are any outliers these must be looked at in closer detail by the user.

- - If any curves are identified which the user disagrees with they may either change the curve to “inconclusive” using the “Edit sample” tag or the user can use the eyemark tool to force the second reviewer to look at this

sample.
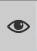


- - Once happy with the plate the user presses Approve results to send the plate to the second reviewer.


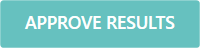


- - The user then has the opportunity to add comments. In this section an overview of any changes or issues on the plate should be given and finally it must be confirmed that the reviewer agreed with “all (remaining) plugin decisions” as appropriate.
- Second approver selects the plate in the “To be reviewed” section
  - Initially second approver opens the “To be reviewed” tab. Here will be the comments from the first reviewer along with any samples which were reviewed by the first reviewer plus any additional samples which they marked for review or adjusted. Second approver must review each well and press the green tick to confirm or adjust the results and then confirm.
  - Once all wells which have been highlighted to review have been confirmed the second reviewer then follows the same process as for the first reviewer detailed above.
  - Once happy with the plate the second reviewer presses authorize analysis. The plate will then disappear from the start screen and can be retrieved from the archive.
- Fast finder automatically outputs a report (.csv file, contains sample names and results (pos, neg, void)) to LIMfinity. Note: LIMfinity only accepts the first result received.

# RT-qPCR interpretation and decision tree

- - - The samples and controls are processed and the result assigned as per the decision tree held within the plugin in Fastfinder. The current version is V3.0. This is attached in the appendix.
    - For changes to the decision tree changes must be authorised at the CAB and approved by the NHS if necessary.
    - Under the current decision tree, a sample passes if the sample IEC is < X. The value of X is calculated by one of the options below:
      - If 1 month or more of data are available, then the mean of all sample IEC + 3SD is used. This is to be checked on a monthly basis by the BSO (Data) and updated if necessary.
      - If less data are available or a significant change in assay chemistry has taken place the range is calculated as the mean

+3SD of 5-10 validation plates. This is then checked again after

1 day, 1 week and 1 month with adjustments made as necessary.

- - - - For a change in this threshold (or any other user changeable thresholds in the decision tree) this must be agreed and checked by the BSO (Data), Product owner and QA manager. This must be then updated in the appropriate table in the Appendix and signed by the BSO.
    - On occasion it may be necessary to adjust results outside of the decision tree, for example in cases of contamination or other failure in the process. Details for this can be found in the production guidance.

# RESPONSIBILITIES

- - - It is the responsibility of all personnel handling data to follow this procedure.
    - It is the responsibility of personnel performing this procedure to ensure that proper documentation is maintained.
    - It is the responsibility of the BSO (Data) to oversee all training of staff in this SOP and Fastfinder software.
    - It is the responsibility of the BSO (Data) and Data strategy manager to ensure that this procedure is followed and that it is updated as necessary.

End of SOP CB 17 Version6

**SOP CB 33: Oropharyngeal and nasopharyngeal swab sample manual preparation and viral deactivation prior to PCR preparation for ‘Direct to PCR’ (Version2 abridged)**

1. **PURPOSE**

The Cambridge COVID-19 Testing Centre (CCTC) based at the Anne McLaren Building is one of the National Testing Centres for COVID-19. The purpose of this facility is to test for the presence of the SARS-CoV-2 virus. This procedure provides the method for preparing and deactivating human swab samples prior to further PCR preparation in a safe and consistent manner.

1. **AUDIENCE**

This SOP applies to all on site trained personnel who are responsible for assessing samples for COVID-19 screening.

1. **SCOPE**

This document defines the required procedure for Station 1, incorporating the sorting and inactivation of viral particles from swab samples with the purpose of detecting the presence of SARS-CoV-2 viral RNA using the Primerdesign Ltd COVID-19 Genesig Real-Time PCR (HT 2.0 V1) assay kit.

1. **GLOSSARY**

| **Terms** | **Definition** |
| --- | --- |
| CB | Cambridge |
| SOP | Standard Operating Procedure |
| SARS-CoV-2 | The virus that causes COVID-19 |
| COVID-19 | Coronavirus disease 2019 |
| DNA | Deoxyribonucleic Acid |
| RNA | Ribonucleic Acid |
| PCR | Polymerase Chain Reaction |
| PoU | Point of use |
| RT-PCR | Reverse Transcription - Polymerase Chain Reaction |
| PPE | Personal Protective Equipment |
| BSC | Biological Safety Cabinet |
| PCT | Positive Control Template |
| NTC | No Template Control |

1. **SAFETY OVERVIEW**

# Hazards

| **Reagent** | **Hazard** | **Hazard Statement(s)** |
| --- | --- | --- |
| **RNase-free water** | Non-hazardous | N/A |
| **Distel (1:10)** | Non-hazardous | N/A |
| **70% Ethanol** | 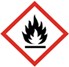 | H225 Highly flammable liquid and vapour, H319 Causes serious eye irritation |

- 1. **Personal Protective Equipment**
     - Fully buttoned-up Howie-style laboratory coat in the general facility
     - Extended-cuff nitrile gloves – double gloved when working within the BSC
     - Disposable sleeves for non-heat inactivated samples (required if lab coat does not have elasticated cuffs, or if lab coat is non-disposable or not liquid- impermeable)
     - Laboratory safety glasses or over-glasses (Where vision is impeded when used with masks and the operator is positioned at a BSC and protected, they may temporarily work without glasses)
     - All PPE should be removed before exiting the sample prep rooms

1. **PROCEDURE**

# General considerations

## Warnings and precautions

- - - - Handle all specimens as if infectious
      - Refer to task-based risk assessment for handling infectious specimens
      - Discard all gloves, pipette tips, vials, test tubes, or other disposable items into appropriately labelled biohazard boxes or autoclave bags.
      - Use separated working areas for specimen preparation, reaction set up and amplification, with separate supplies and equipment

## Preventing Contamination

- - - - Change gloves whenever contamination is suspected and change outer glove when changing workstation (e.g. station 1a to station 1b)
      - BSC, workbenches and pipettes should be cleaned and decontaminated with 1:10 Distel (v:v), and 70% (v:v) ethanol to minimize risk of contamination. Spray and wipe the surfaces with Distel followed with 70% ethanol.
      - Reagents and equipment should not be moved from a dirty area to a clean area. Should a case arise where a reagent or piece of equipment needs to be moved backwards (dirty to clean), or contamination is suspected, it must first be decontaminated with Distel and wiped down with 70% ethanol.

## Preventing DNase/RNAse contamination

- - - - Use DNase/RNase-free disposable plasticware and pipettes reserved for DNA/RNA work to prevent cross-contamination with DNases/RNases from shared equipment
      - Use DNase/RNase-free filter tips throughout procedure to prevent aerosol and liquid contamination
      - Wipe surfaces of workbenches and BSC with RNAse Away followed by 70% ethanol

## Exposure control plan

In case of sample spillage or any other risk of exposure to SARS-CoV-2 arising, the following measures need to be taken.

#### 6.1.4.1 Spillage on bench or in BSC for **heat inactivated** samples

- Mop up the liquid using absorbent pads/paper and discard in the biohazard bin on the workbench
- Treat area of spillage with Distel (1:10), RNase away and 70% ethanol
- Remove top layer of gloves and dispose of in biohazardous waste.
- All spills should be reported to the lab lead

#### 6.1.4.2 Spillage outside the BSC for non-heat inactivated samples

- All scientists discard any PPE that may have been in contact with contaminated sample as a hazardous waste and leave the room.
- Immediately contact team leader or shift leader and await further instruction, scientists are to remain in the vicinity and not disperse into other rooms.
- The team leader/shift leader will enact the “Uncontained Spill Procedure” as outlined in SOP CB 09.

#### 6.1.4.3 Spillage inside the BSC for non-heat inactivated samples

- Mop up the liquid using absorbent pads/paper and discard in the biohazard bin in the BSC
- Treat area of spillage with Distel (1:10) for 30 min
- Remove top layer of gloves and sleeves inside the BSC
- After 30 min follow with a wipe with 70% ethanol. Discard waste tissue / paper towel in the biohazardous bin in the biosafety cabinet
- Remove top layer of gloves and sleeves inside the BSC
- All spills should be reported to the lab lead

#### 6.1.4.4 Contamination of PPE with any biological sample

- Immediately encapsulate all potentially contaminated PPE

Wash hands/exposed skin according to best practice

# Procedure Schematic


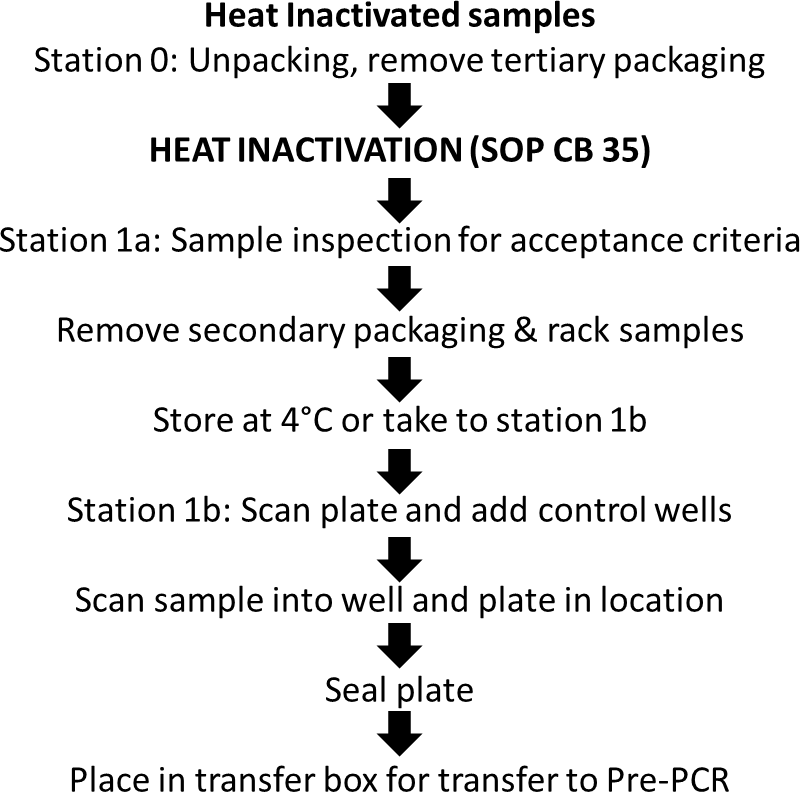


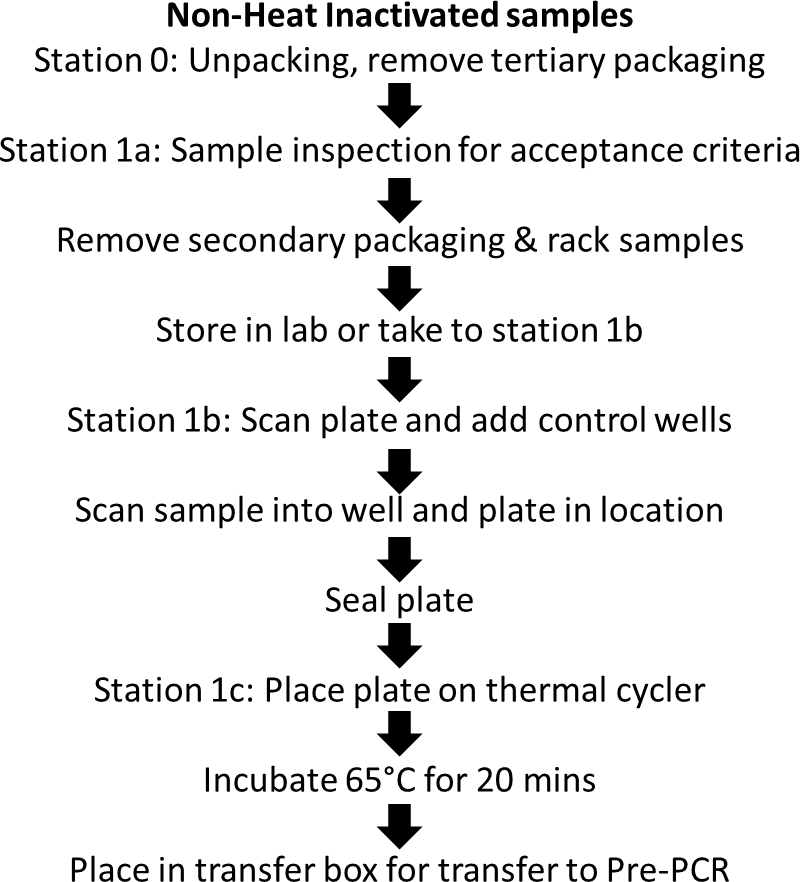


- 1. **Workstation Schematic**


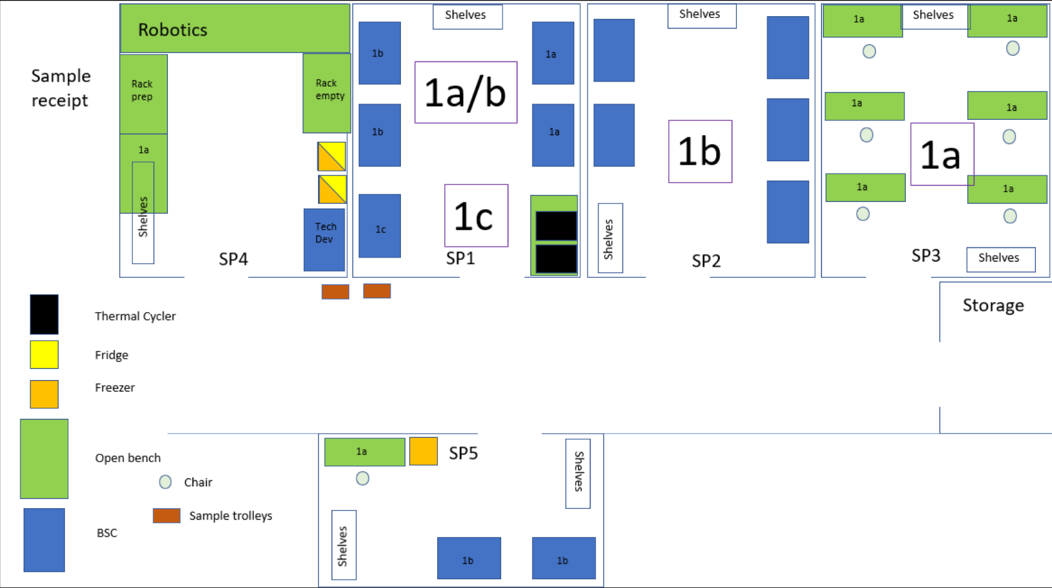


For reference, the layout of the Sample Preparation Laboratory

# Materials and Reagents

| **Reagent / Labware** | **Vendor and article#** | **Comment** |
| --- | --- | --- |
| Bio-rad PCR plate | Bio-Rad | Supplied by Avantor via PoU |
| Foil plate seal | Beckman | Supplied by Avantor via PoU |
| 50mL Reservoir | Corning, 4870 | Supplied by Avantor via PoU |
| P1000 extended tips, filtered | Sartorius, Thermo Fisher, Jet Bio | Supplied by Avantor via PoU |

| **Equipment** | **Comment** |
| --- | --- |
| Single channel pipette P1000 | Sartorius, 613-5483, Eppendorf,  Thermo Scientific |
| Roller for adhesive tape | Found in lab |
| Barcode scanner | To scan each barcoded swab vial and plate |
| Waste container for tips and tubes | Biobins |
| LIMS laptop | To log samples and plate |
| Thermal cycler | PCR Max Alpha Cycler AC4 |

- 1. **General guidelines**
     - Perform all steps at room temperature (20-25^o^C), unless otherwise noted.
     - Perform all steps wearing appropriate PPE
     - Avoid creating bubbles during mixing and aliquoting

# Procedure

- - - Perform all actions with non-heat inactivated samples in a Class 2 BSC
    - To avoid confusion, all work with non-heat inactivated samples is carried out in a dedicated room
    - Refer to plate map for guidance
    - All waste including tips should be placed in Bio-bins, sealed and double contained in an autoclave bag if necessary, before leaving the BSC or workbench. Refer to Risk Assessments and Waste Management Section for guidance.
    1. **Sample preparation: Station 1a for non-heat inactivated samples**

*Note: Samples that have* ***not been successfully heat treated in the oven*** *will be clearly labelled and processed in a BSC. All samples that come out of this hood will also be clearly labelled for a second heat inactivation (Section 6.6.5)*

- - - 1. Ensure the BSC has been tested in the last 12 months and is within operational safe working limits. Airflow information on the front of the cabinet should not be in red and no alarms should be sounding. If hood is non-compliant do not proceed and report to the shift lead.
      2. Wipe down inside of biological safety cabinet with Distel (1:10) followed by 70% ethanol, discarding wipes in biohazard waste container in the BSC.
      3. Obtain the delivered container of samples from the Input zone. Remove sample bags from the container (tertiary packaging), placing them in the BSC.
      4. Inspect the vials for leaks and ensure they meet the acceptance criteria.
         1. The volume of transport media should be sufficient*
         2. The sample tube should be intact
         3. The sample should contain a single swab (with the exception of samples from University of Cambridge which will contain multiple swabs)
         4. A barcode should be present on the swab tube
         5. The sample should not contain visible contamination with mould

**Volume should be greater than 100 µl but not fill the sample tube as this is an error.*

- - - 1. If samples fail inspection, the operator should ensure the sample is double contained. An additional bag should be used if needed. Then mark the bag with a number corresponding to the spoil reason (see Section 10 Appendix).
      2. The operator should place the sample in a plastic container labelled ‘Void’ whilst still in the BSC to triple contain the samples. Set aside the box and inform lab or shift lead for recording in LIMfinity and disposing as per SOP CB 10.
      3. If the sample is accepted, remove the vial from the secondary packaging, discard packaging in an autoclave bag placed within the BSC.
      4. Place the vial in the racking contained in a Tupperware container lined with absorbent mat for secondary containment.
      5. Once a rack is full, secure the lid, wipe down the external surfaces with Distel (1:10) and 70% ethanol. The Tupperware is then taken to Station 1b and placed in the pre-processing input area (fridge/shelves).
      6. At end of working period, repeat 6.6.1.2 to clean the BSC.

## Sample preparation: Station 1a for heat inactivated samples

- - - 1. Wipe down the work area with Distel (1:10) followed by 70% ethanol, discarding wipes in biohazard waste container next to the workbench.
      2. Obtain the delivered container of samples from the input zone. Remove sample bags from the container (tertiary packaging), placing them on the work area.
      3. Inspect the vials to ensure they meet the acceptance criteria:
         1. The volume of transport media should be sufficient*
         2. The sample tube should be intact
         3. The sample should contain a single swab (with the exception of samples from University of Cambridge which will contain multiple swabs)
         4. A barcode should be present on the swab tube
         5. The sample should not contain visible contamination with mould

**Volume should be greater than 100 µl but not fill the sample tube as this is an error.*

- - - 1. Inspect the sample bags for moisture and leaks:
         1. If the outer bag is damp from the heat inactivation, there is no change to procedure, continue to process samples as normal
         2. If moisture is seen between the inner and outer bag, carefully open outer bag and remove inner bag to check if moisture is also visible within the inner bag. If no moisture is visible, process as normal. If moisture is present within the inner bag follow point c.
         3. If moisture is seen within the inner bag: Handle the sample vial carefully, avoiding contact with liquid, wipe the tube with a Distel (1:10) tissue and

rack as normal. Dispose of all used tissue in autoclave waste bag after each vial is wiped. Change outer gloves.

- - - - 1. *Note: Use the void process for significant leaks where outer vial contamination with liquid cannot be effectively wiped*
      1. If samples fail inspection, the operator should ensure the sample is double contained. An additional bag should be used if needed. Then mark the bag with a number corresponding to the spoil reason (see Section 10 Appendix). Place the sample in a plastic container labelled ‘Void’ whilst still working at the workbench. Set aside the box and inform lab or shift lead for recording in LIMfinity and disposing as per SOP CB 10.
      2. If the sample is accepted, remove the vial from the secondary packaging, discard packaging in an autoclave bag placed next to the workbench.
      3. Place the vial in the racking contained in a Tupperware container lined with absorbent mat for secondary containment.
      4. Once a rack is full, secure the lid and take to Station 1b and place in the pre- processing input area (fridge/shelves).
      5. At end of working period, repeat 6.6.2.1 to clean the workbench.

## Sample preparation: Station 1b

*Note: This procedure is conducted by two operators. Operator One will conduct the sample work within the BSC. Operator Two will shadow, checking samples and reagents are added to the correct wells and operating the electronic LIMS system ensuring correct recording. Where the operator is not defined, this step may be completed by either.*

*Note: For full technical details on working with the LIMS system LIMfinify and Viral Prep Tool App see user guide COVID19 IT training.*

*Note: Take note of Tupperware containers coming out of 1a. If it states that the samples have not been heat inactivated then outer sleeves and doubles gloves should be worn by Operator 1 through the procedure, with all items that are removed from the hood being thoroughly cleaned with Distel and 70% ethanol. If samples have been heat inactivated, then Operator 1 will require double gloves but not outer sleeves and will only be required to wipe items out of the hood with Distel and 70% ethanol if they are suspected to be contaminated.*

- - - 1. Operator 1 will sign into the Viral Prep Tool App and with their credentials as they will be the one conducting the wet work in the BSC.
      2. Ensure BSC is compliant and within operational safe working limits. All work to be conducted in the BSC unless otherwise stated.
      3. Sequentially wipe down inside of BSC with Distel (1:10), RNAse Away and 70% ethanol. Clear up residue at each step with tissue / paper towel and discard in biohazard waste container in the hood.
      4. Waste containers consisting of Bio-bins in an autoclave bag should be placed in the hood, ensuring the airflow is not obstructed, ready for sample and waste discarded during the process.
      5. Remove a Qnostic Positive Control from the -20°C freezer in Sample Prep (aliquots of working stock will be stored at -20°C with bulk stock kept at -80°C). Allow to defrost at room temperature. Ensure a barcode is taken from the box when collecting the vial.

*Note: The Qnostic positive control is to be added to every sample prep plate when possible. Its position is to be randomly placed in the plate during steps 9- 11 of Section 6.6.4. The app will suggest a well for the Qnostic (in yellow), where possible pipette into the suggested well.*

- - - 1. Collect a Tupperware containing racked samples from the pre-processing input area (fridge or shelves) or station 1a.
      2. Once Operator 1 is positioned in the hood with the correct PPE, Operator 2 is to pass an empty Hard-Shell Low-Profile Skirted 96-Well PCR Plate into the BSC and a barcode. Operator 1 will attach a barcode to the left-hand side of the plate. Lifting it to the glass, Operator 2 will scan the plate barcode into the top box on the app which is selected in blue.

*Note: if any problems should occur at the start and the app does not work, inform the lab lead and switch to the previous excel sheets as detailed in the appendix.*

- - - 1. Operator 1: Next using a P1000 Pipette, add 100 µL nuclease free water to wells A1-B1 for the controls as shown in the plate layout.
      2. Operator 1 will remove the first sample vial and hold to the glass for operator 2 to scan into the sample box highlighted in blue. Sample will be assigned a well location by the app. Operator 2 will call the well location.
      3. Operator 1 will remove the lid over a Bio-bin, discarding it in the waste, and using a P1000 pipette with extended tips, transfer 100 µl to the designated well. This will be verified by operator 2. The vial will be placed in the waste.
      4. Repeat for the remaining sample vials, take column-wise from input rack, transfer to sample plate column-wise, scanning each vial in and discarding them in the Bio-bin / autoclave bag. One sample space should be used for the Qnostic positive control as stated in step 5 of section 6.6.4.

*Note: Should any problem occur with either the sample or pipetting of the sample, immediately alert the lab lead or designated super user of the sample prep tool app. Lab lead will follow instructions as stated in SOP CB*

*10. This is not to be conducted by the operators.*

*If barcodes do not scan correctly, manually enter the sample ID with verification from the other operator and hit enter. Inform lab lead of any issues.*

- - - 1. Operator 1: Seal plates with Beckman foil seal.
      2. For non-heat inactivated samples transfer the plate into two sample bags to double contain. Wipe with Distel (1:10) and 70% ethanol and remove from the hood and transfer to Station 1c in the non-heat inactivated input area. Inform the operator manning Station 1c to continue with Section 6.6.5.
      3. For heat inactivated samples the plate should be contained in a labelled transfer box to be taken to the plate output area for transfer to Pre-PCR, without the necessity to wipe the box with Distel and 70% ethanol.
      4. Operator 1: Discard any reservoirs or waste into the waste bin in the BSC avoiding any spillage as defined in 6.6.6 Waste Management. Clear working area and wipe down surface and pipettes with Distel (1:10) and 70% ethanol as required. Seal the waste bins taking particular note of the biobins containing the remaining sample liquid, close the autoclave bag and pass to Operator 2 who will ensure bins are correctly sealed and aid placement into a second bag. Remove outer gloves and sleeves as bins are removed or place in a new Bio- bin in the BSC.

## For Non-Heat Inactivated samples: 1c Alpha Cycler 4 PCRmax

- - - 1. To start the thermal cycler, should it have been switched off, press the rocker switch on the right-hand side of the machine to the “on” position. Allow the unit to boot up to the Home Screen. Select the Run Program button and choose program “Sample Prep 1c”. Select all the blocks required (Quadrant A, B, C, D); one block/plate.
      2. Start the programme. The unit will indicate “Preparing” - this is for heating the lid. The program will then start heating the block to 65°C. This will take ~ 1 min.
      3. When the display indicates “Preparing” for a second time, select the block required.
      4. The heat inactivation program will be displayed. Select Pause and confirm Pause.
      5. Press backspace, this can be found on the bottom left corner of the screen.
      6. Repeat section 1 for each block.
      7. Before removing plate from double containment ensure that the plate seal has been applied correctly. Scan in the plate ID on the Viral Plate Tracker and initial to confirm seal is correctly applied.
      8. Remove plate from double containment in designated input area at the 1c/Heat Inactivation station.
      9. Open the lid by pressing the smaller blue button on the lid. Insert the plate making sure that it is properly seated and close the lid.
      10. On the home screen select the block you have just closed and press the resume button at the bottom of the screen. Repeat for each block needed.

*Note: The status of each block can be observed by selecting it in the home screen. If a block is indicating “Idle” it is not at a set temperature or running a program.*

- - - 1. When the program reaches the “Final Store” portion, press the finish button. This portion of the program will run continuously unless manually stopped. Remove the plate from the thermal cycler and place in the plate output area for transfer to Pre-PCR (SOP CB 27).

*Note: The thermal cycle operator will sign off, using the tracking sheet provided, that the plate has successfully completed the Heat Inactivation cycle.*

- - - 1. If all programs have finished the unit can be powered down. No shutdown sequence is required.

## Plate Layout

|  | **1** | **2** | **3** | **4** | **5** | **6** | **7** | **8** | **9** | **10** | **11** | **12** |
| --- | --- | --- | --- | --- | --- | --- | --- | --- | --- | --- | --- | --- |
| **A** | PCT | S06 | S14 | S22 | S30 | S38 | S46 | S54 | S62 | S70 | S78 | S86 |
| **B** | NTC | S07 | S15 | S23 | S31 | S39 | S47 | S55 | S63 | S71 | S79 | S87 |
| **C** | S01 | S08 | S16 | S24 | S32 | S40 | S48 | S56 | S64 | S72 | S80 | S88 |
| **D** | S02 | S09 | S17 | S25 | S33 | S41 | S49 | S57 | S65 | S73 | S81 | S89 |
| **E** | S03 | S10 | S18 | S26 | S34 | S42 | S50 | S58 | S66 | S74 | S82 | S90 |
| **F** | S04 | S11 | S19 | S27 | S35 | S43 | S51 | S59 | S67 | S75 | S83 | S91 |
| **G** | S05 | S12 | S20 | S28 | S36 | S44 | S52 | S60 | S68 | S76 | S84 | S92 |
| **H** | CBIQA | S13 | S21 | S29 | S37 | S45 | S53 | S61 | S69 | S77 | S85 | S93 |

**Key:**

PCT: Positive control template NTC: No template control

S: Sample well

## Note:

A random well designated for Qnostic control is highlighted in “gold” as a suggestion (for example, well H1 in this diagram).

## Waste Management

- - - 1. Station 1a
         - All plastic packaging waste will be discarded in the plastic biohazard autoclave bags
         - Clean outer gloves with Distel (1:10) followed by 70% ethanol and dispose of into the autoclave bag
         - Remove the sleevelet and dispose of into the autoclave bag
         - Seal the autoclave bag with an autoclave tape ready for autoclaving
      2. Station 1b
         - Lids may be secured on sample tubes dependant on style and swab placement or left off. Place in the 2L Bio-bin in the BSC. Before removal from the BSC, ensure everything is sealed and double contained using autoclave bag
         - Decontaminate the accessible surface of the 96-tube rack with Distel (1:10) followed by 70% ethanol
         - Decontaminate any tip boxes for disposal with Distel (1:10) and 70% ethanol and place in the autoclave bag if passed out the hood.
         - Any small quantities of waste liquid (less than 50 ml) should be placed in a falcon tube, sealed and disposed of into a Bio-bin within the BSC. Larger volumes of liquid waste from buffers should be poured into a suitable container for autoclaving
         - Dry reservoirs should be disposed of in the Bio-bin in the BSC
         - Seal the Bio-bins and close the autoclave bag, pass outside the BSC to be placed in a second autoclave bag.
         - Clean the outer gloves with Distel (1:10) followed by 70% ethanol and place them into the autoclave bag when being discarded.
         - Sleeves to be discarded into an autoclave bag.
         - Using clean gloves or a second operator, seal the autoclave bags with an autoclave tape ready for autoclaving.
      3. Station 1c
         - Operator 1 will remove outer gloves and place in autoclave bag at workstation.
         - Operator 1 will place bags previously containing samples plates prior to heat inactivation into an autoclave bag at workstation.

1. **RESPONSIBILITIES**

- It is the responsibility of all personnel handling samples to follow this procedure
- It is the responsibility of personnel performing this procedure to ensure all equipment, chemicals, reagents, and solutions are properly labelled and that proper documentation is maintained
- It is the responsibility of the laboratory lead to ensure that this procedure is followed and that it is updated as necessary

End of SOP CB 33 (Version2)

**SOP CB 34: STATION 3 DIRECT TO PCR; ARRIVAL, STORAGE, AND RECONSTITUTION OF REAGENTS (Version2 abridged)**

1. **PURPOSE**

The Cambridge COVID-19 Testing Centre (CCTC) based at the Anne McLaren Building is one of the National Testing Centres for COVID-19. The purpose of this facility is to test for the presence the SARS-CoV-2 virus. This procedure provides the method for use and storing of the different components of the Genesig Real Time PCR COVID-19 High Throughput (HT-CE kit V2.0) assay kit prior to preparing the PCR plates and the addition of the Internal Control (IEC) during the sample preparation step.

The assay will be run using a 10µl total reaction volume in a 384-well qPCR plate.

1. **AUDIENCE**

This SOP applies to all on site trained personnel who are responsible for assessing samples for COVID-19 screening.

1. **SCOPE**

This document defines the required procedure for Station 3 for use and storing of the different components of the Primerdesign Ltd COVID-19 Genesig Real-Time PCR (HT-CE kit V2.0) assay kit. This method describes the method of preparing and dispensing the RT-PCR Mastermix into the 384-well PCR plate. Addition of No Template Control (NTC), Internal control (IEC) and sample addition. See SOP for addition of Positive Control to the PCR plate and running of the LightCycler 480 II. With the purpose of detecting the presence of nucleic acid from SARS-Cov-2 in oropharyngeal and nasopharyngeal swabs.

1. **GLOSSARY**

| **Terms** | **Definition** |
| --- | --- |
| SARS-CoV-2 | The virus that causes COVID-19 |
| COVID-19 | Coronavirus disease 2019 |
| DNA | Deoxyribonucleic Acid |
| RNA | Ribonucleic Acid |
| PCR | Polymerase Chain Reaction |
| RT-PCR | Reverse Transcription - Polymerase Chain Reaction |
| PPE | Personal Protective Equipment |
| BSC | Biological Safety Cabinet |
| PCT | Positive Control Template |
| NTC | No Template Control |
| IEC | Internal Extraction Control |
| min | Minute |

1. **SAFETY OVERVIEW**

# Hazards

| **Reagent** | **Hazard** | **Hazard Statement(s)** | **Reference** |
| --- | --- | --- | --- |
| **Primerdesign Ltd COVID-19**  **Genesig Real- Time PCR (HT-CE**  **kit V2.0) assay** | Non- hazardous |  |  |
| **70% Ethanol** | 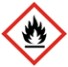 | H225, H319 | MDC COSHH 048 |
| **Distel (1:10)** | Non- hazardous | H315, H317, H318, H351, H373, H412 | MDC COSHH 004 |

- 1. **Personal Protective Equipment**
     - Pre-PCR: Fully buttoned-up Howie-style laboratory coat
     - Standard nitrile gloves
     - Laboratory safety glasses or over-glasses

1. **PROCEDURE**

# General considerations

## Warnings and precautions

- Treat all specimens as a biohazard.
- Refer to the task-based risk assessment.
- Discard all gloves, pipette tips, pipettes, vials, test tubes, or other disposable items into appropriately labelled sharps biohazard boxes or autoclave bags.
- Use separated working areas for specimen preparation, reaction set up and amplification, with separate supplies and equipment

## Preventing Contamination

- Incorrect results could occur if either the clinical specimen or the real-time PCR reagents used in the amplification step become contaminated by accidental introduction of amplification product (amplicon) or positive control reference material.
- The Genesig COVID-19 positive control template is provided in a sealed foil envelope and contains a high copy number of templates. It should be opened and processed away from test samples and kit components to avoid cross- contamination.
- Change gloves between samples and whenever contamination is suspected.
- Keep reagent and reaction tubes capped or covered as much as possible
- Always check the expiration date prior to use. Do not use expired reagent. Do not substitute or mix reagent from different kit lots.
- Work surfaces, Biological Safety Cabinet, pipettes, and centrifuges should be cleaned and decontaminated with cleaning products (e.g. DNA/RNA remover, 70% ethanol, Distel 1:10) to minimize risk of nucleic acid contamination.
- In the situation that contaminated plates are identified during the data analysis or a spill of the PCT occurs within the BSC, decontaminate the BSC used for handling/dispensing Mastermix and PCT using UV lighting.
- Change gloves whenever contamination is suspected and when changing work station
- Reagents and equipment should not be moved from a dirty area to a clean area. Should a case arise where a reagent or piece of equipment needs to be moved backwards, it must first be decontaminated with Distel 1:10 and wiped down with 70% ethanol.

## Preventing DNase/RNase contamination

- Use DNase/RNase free disposable plasticware and pipettes reserved for DNA/RNA work to prevent cross-contamination with DNases/RNases from shared equipment.
- Use DNase/RNase free filter tips throughout procedure to prevent aerosol and liquid contamination.
- Wipe surfaces of workbenches and biological safety cabinets with RNase away followed by 70% ethanol

# Procedure Schematic


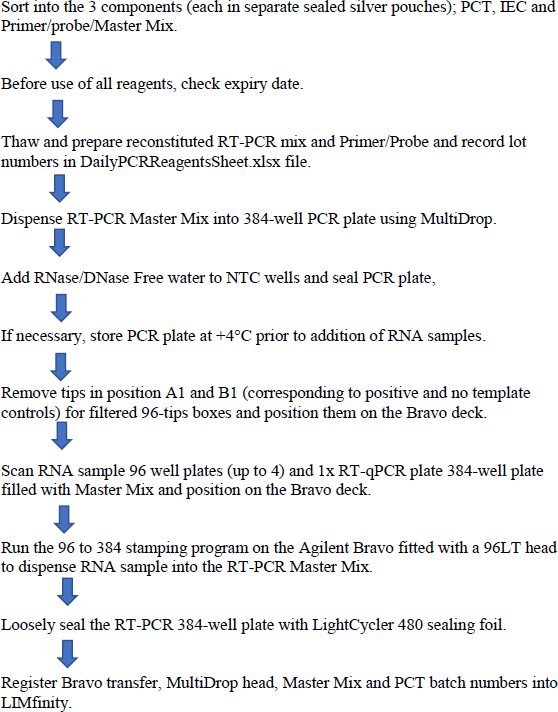


- 1. **Work Station Schematic**


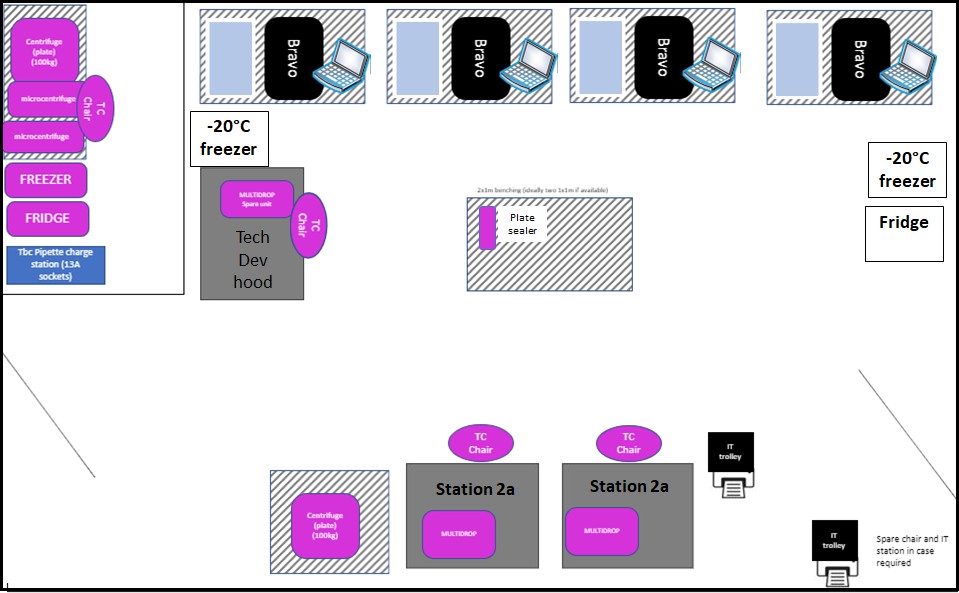


For reference, a typical layout of a Station 3 RT-PCR Preparation Laboratory

# Materials and Reagents

| **Reagent / Labware** | **Comment** |
| --- | --- |
| COVID-19 RT qPCR assay (HT-CE kit V2.0) kit  from PrimerDesign LTD |  |
| P1000 tips, sterile, filtered |  |
| P200 tips, sterile, filtered |  |
| Distel |  |
| Ethanol 70% spray |  |
| Aluminium foil | To protect RT-PCR probes from light |
| RNase Away |  |
| 10 mL strippettes |  |
| 5 mL stripettes |  |
| Falcon tubes (50 and 15 mL) |  |

| **Equipment** | **Comment** |
| --- | --- |
| BSC hoods dedicated for PCR Master Mix and  IEC preparation |  |
| Fridge (+4°C |  |
| Freezer (-20°C) |  |
| Single channel pipette P1000 |  |
| Single channel pipette P200 |  |
| Vortex |  |
| Benchtop centrifuge for microtubes |  |
| Containers for microtube | e.g. NUNC Cryobox or plastic bag |
| Waste container for tips and tubes |  |
| Ice box containing ice |  |

- 1. **General guidelines**
     - Perform all steps in a BSC to avoid contamination unless otherwise noted
     - Perform all steps wearing appropriate PPE
     - Avoid creating bubbles during mixing and aliquoting

# Reagent arrival, storage, and preparation

- - - The Primerdesign Ltd COVID-19 Genesig Real-Time PCR (HT-CE kit V2.0) assay is shipped on **dry ice**, store at **-20°C** on arrival.
    - Always check the expiration date prior to use. Do not use expired reagents.

## Protect fluorogenic primer/probe mix from light.

- - - The kit contains 3 packages.
      - Pack 1: Primer/probe mix and the enzyme Mastermix
      - Pack 2: Lyophilised positive control template and template preparation buffer
      - Pack 3: Internal extraction controls are all delivered ready to use in solution.
- Once resuspended, components are stable for up to six months if stored at - 20°C and for at 4°C or the expiry date, whichever occurs first.
- Whilst in use, the kit components should be kept on ice or stored at 4°C in the dark to minimize the time at room temperature.

## The reagents should not be freeze/thawed.

- Once defrosted the components are stable at 4°C for up to 3 months according to manufacturer's specifications.
- Each time a component is defrosted, the date will be written on the outer packaging/container if it is not used immediately. There is a Mastermix inventory located in the internal Teams: Cambridge Coronavirus test Centre Teams/3.PCR/Files/Inventory Mastermix where all current lab stock is logged along with its location, Lot number and expiry date. As a kit is used, the stock will be deleted from the inventory.

## Kit components

One kit of COVID-19 RT qPCR assay (HT-CE kit V2.0) kit from PrimerDesign LTD will enable testing of **1536 samples** (4 x 384-well qPCR plates) and contains the following:

The COVID-19 Primer & Probe Mix contains the primers and FAM labelled probe specific to SARS-CoV-2 and the primers and HEX labelled probe specific to the Genesig Easy RNA Internal extraction control (IEC).

| **Reagent label** | **Number of vials**  **(1536 tests)** | **Volume mL/vial** | **Lid colour** | **Resuspended with?** |
| --- | --- | --- | --- | --- |
| **Pack 1** | | | | |
| **4x Precision Tough mix** | 1 | 9.5 | Orange | N/A |
| **COVID-19 Primer & Probe Mix (including IEC primer/probe mix)** | 1 | 3.8 | Green | N/A |
| **Pack 2** | | | | |
| **Genesig COVID-19 Positive control template** | 1 | 0.6* | Red or pink | Template preparation buffer |
| **Template preparation buffer** | 1 | 1.5 | Yellow | N/A |
| **Water (RNase/DNase free)** | 1 | 1.5 | Clear or white | N/A |
| **Pack 3** | | | | |
| **Genesig COVID-19 Internal extraction control (IEC)**** | 16 | 2.62 | Green | N/A |

*Projected volume once resuspended

****Note:** tube is labelled Internal Extraction Control. In direct to PCR this reagent is an Internal control as extraction is not performed.

## Collect assay kits

Collect all RNA Internal Extraction Control silver packets (packet 3) and transfer to -20°C storage.

Collect all COVID-19 Positive Control Template silver packets (packet 2) and transfer to

-20°C storage in PCR station 3b.

Collect all primer/probe silver packets (packet 1, also contains Mastermix) and transfer to -20°C storage in PCR station 3a.

Complete stock inventory detailing number of kits, lot numbers as detailed on the label on the outer silver packet (not the individual inner vial) and storage location.

## Reconstitution of 4x Master Mix and Primer/Probe mix

1. Collect packet 1 from -20°C storage and place Mastermix (orange lid) and Primer/probe mix (green lid) on ice. Ensuring the primer/probe mix is **protected from light.**
   1. Alternatively, the COVID-19 Primer & Probe Mix can also be defrosted overnight at 4^°^C in preparation for use by the next shift.
   2. One pack contains sufficient prime/probe mix and Mastermix to prepare 9 x 384-well qPCR plates (including excess) adding 3.5µl/ well. Two packets contain sufficient reagent to prepare 19 x 384-well qPCR plates (including 1ml excess)
   3. Primer/probe mix and Mastermix can be stored at 4°C the date and time of defrosting will be written on the packet which also stated the expiry date.
2. Prepare the reaction mix: Prepare the reaction mix: In a BSC measure all reagents based on the volumes in the table below using a stripette or pipette as appropriate into a 50mL falcon tube. Replace the cap and invert the tube to mix.


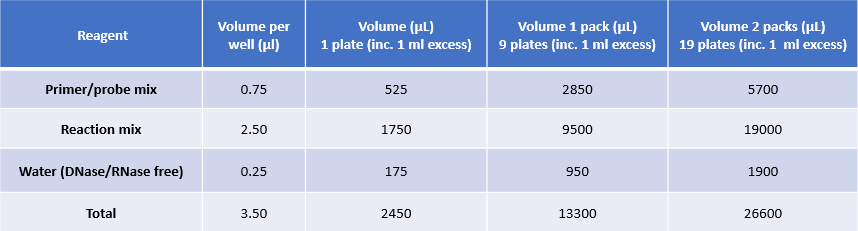


- 1. If multiple packets are being used at once, combine each of the packets as described in step 2 before pooling the reaction mix in 1 x 50mL Falcon tube. Take care to only pool reagents of the **same lot number**. Falcon tubes should be labelled with Lot number, reconstitution date and the number of kits used.

**Note:** Do not add the primer/probe solution to the Mastermix until you are ready to use the reaction mix. If the components are being defrosted or stored at 4°C they should be kept as separate components.

1. Reconstituted reaction mix should be kept on ice for immediate use. Do not freeze thaw.
2. Proceed to prepare the 384-well qPCR plates.
3. Clean all surfaces, pipettes, and tip racks in the BSC with Distel and 70% ethanol.

# Preparation of 384-well PCR plate and addition of negative control

## Protect fluorogenic primer/probe mix from light

- - - When not in use the thawed Mastermix and primer/probe mix should be stored on ice

## Dispense of the RT-PCR mix into the 384-well PCR plate using MultiDrop At the beginning of the day, before first run:

- - - 1. If not already in place, put and secure 8 tubes small cassette in a MultiDrop Combi machine located in a BSC. Check that the tubes are free of liquid.
      2. Switch on the MultiDrop Combi machine and set up the following parameters for the 10ul assay volume. **The witness will confirm correct settings.**
         1. 384 well Low Profile 10 mm plate
         2. Dispense 3.5ul using small cassette
         3. Dispense in Full plate (columns 1 to 24)
         4. Speed should be Medium with z-axis height of 12mm
         5. **Note:** It is acceptable to generate half plates by filling even number columns and adding sample to quadrants (Quadrant 1 and Quadrant 3). **This should be witnessed and Multidrop returned to standard settings after use**.
      3. Clean the cassette by priming first with 70% Ethanol and then RNase/DNase free water (around 25 mL each). **Check that dispense tips are not blocked**.
      4. Run a test plate using RNase/DNase free water to check head alignment and that dispensing performs correctly. To ensure correct catch, check that qPCR plate **position A1 is in the bottom right corner of the loader**.
      5. Empty tubes and release cassette. Cleaning of the multidrop cassettes is signed off on the daily start up and shut down lists for station 3a and 3b.

**At each run:**

1. Remove the required number of qPCR plates from the packet in the BSC. Remove Roche plate barcode and cover with a PCR specific barcode starting ‘003’ on the north long side of the plate. Using the black marker pen also mark a black dot on the top left corner of the plate (near well A1) to orient the plate. Ensure that no other pen marks are on the plate or near any of the wells and the PCR plates are not misshapen.
2. Ensure that the multidrop tubes are free of liquid. If not, clean the head as described above.
3. Check that the dispense parameters are correct (see settings above).
4. Put the tubes at the bottom of the PCR Master Mix 50 mL Falcon tube and prime the system. Check that liquid is dispensed from all 8 tips.
5. Once tubes are primed, put a 384-well PCR plate on the MultiDrop Combi machine and check that **position A1 is in the bottom right corner of the loader**. Dispense 3.5ul for 10ul protocol.
6. Repeat dispensing for the number of PCR plates to prepare. Ensure that enough volume of PCR Master Mix was used by checking that liquid is present in the tubes. Keep the qPCR plate(s) in the hood for NTC addition (see below).
7. After the run, wash the system with first 70% Ethanol and then with RNase/DNase Free water (~ 25 mL each).
8. Finally, prime extensively (~ 30 seconds) with air to dry, release the cassette and put the extremity of the tubes into an empty Falcon in the BSC.

## Add RNase/DNase Free water to No Template Control and positive control template wells.

- - - 1. Collect one vial per qPCR plate of RNase/DNase Free water from the COVID-19 Genesig Real-Time PCR (HT-CE kit V2.0) assay kit stored in the RT-PCR preparation room.
      2. Using either a single or multi dispense pipette: dispense 2.5ul water into the PCT wells of each 384-well PCR plates, corresponding to wells **A1, A2, B1 and B2** (see plate map below).
      3. Using either a single or multi dispense pipette: 10uL assay: dispense 6.5ul water into the No Template Control wells of each 384-well PCR plates, corresponding to wells **C1, C2, D1 and D2** (see plate map below).
      4. Ensure a tip change per vial of water (per plate).
      5. Apply a non-PCR plate seal to the prepared qPCR plate.

**A WITNESS IS REQUIRED TO CHECK THE CORRECT ADDITION OF WATER INTO NTC WELLS**


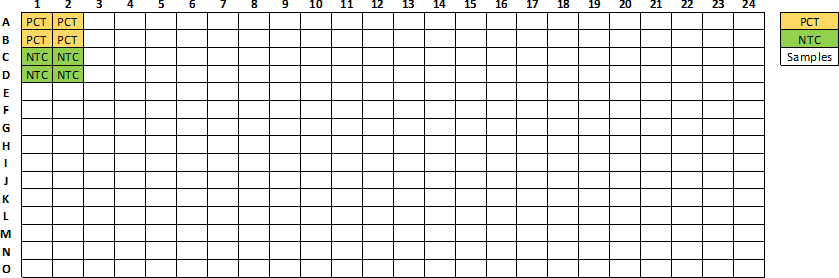


- - - 1. Discard RNase/DNase Free water tube.
      2. Clean the BSC working area with Distel then with 70% Ethanol spray.

## PCR plate processing and storage

- - - 1. After adding the water to the NTC wells, seal PCR plates with e.g. Perkin Elmer Clear Adhesive Film. Plates can now be moved out of the BSC.
      2. For each prepared qPCR plate, record on the plate seal to confirm the addition of the NTC, the date and time of preparation.
      3. Open P:/MultidropHeadScanningSheet_V2 excel spreadsheet on the PC associated with the BSC at station 3a and for each plate record the qPCR plate barcode and the multidrop head used by scanning the barcode for that head.

# Addition of internal extraction control to 384-well RT-qPCR master mix plate

The plate-to-plate transfer of the internal extraction control in 96-well plates into the RT- qPCR 384-well PCR will be done using the Agilent Bravo fitted with a 96LT head. IEC will be added first followed by samples.

## Pre automation: Internal extraction control preparation

1. Remove IEC from -20°C storage and store on ice until required.
2. Dilute sufficient IEC from the number of plates to be prepared.
3. Add IEC as per table below of diluted IEC to a reservoir
   1. This IEC plate can be stored at 4°C for 12 hours. Mark the lid of the plate with the IEC expiry time.


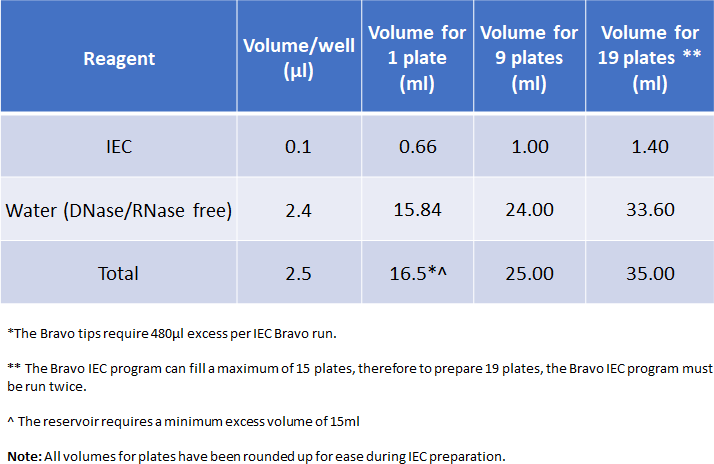


1. Collect sufficient RT-qPCR plate (384-well format) containing Mastermix, NTC and from station 3b PCR preparation fridge. The qPCR plate may need a short centrifugation if there is condensation on the plate seal – 1200rpm for 1 min. Scan plate into PCR tracking sheet (PCR tracking sheet can be found on the CBCTC – Internal, PCR Prep conversation area on Microsoft Teams).

## Automation procedure: Internal extraction control addition

The plate-to-plate transfer of the IEC in a reservoir into the qPCR 384-well PCR will be done using the Agilent Bravo fitted with a 96LT head.

## Before use, clean the Bravo deck and working area around the robot with suitable alcohol wipes or 70% Ethanol and close the VWorks software if open

- Following addition of reaction mix, add IEC to the PCR plate.

REMOVE TIPS FROM A1 & B1

- - 10µl assay: Desktop\Covid Method\D2PCR\IEC_10ul_reservoir
    - Protocol will add 2.5µl IEC to all 4 quadrants up to max 15 plates in one run, Bravo will prompt user to add qPCR plate.
    - See Figure 1 for Bravo set up

## Bravo procedure

- - - 1. Open Bravo VWorks software and login. (Appendix Figure 2)
         - Username = Speak to Lab Leader
         - Password = Speak to Lab Leader
      2. Open the correct protocol from the Desktop Folder
         - **IEC_10ul_reservoir** *(Figure 1)*
      3. If prompted to initialize the device say **“Yes”.** (Appendix Figure 3)
      4. The “MANUAL LOADING INSTRUCTIONS” form should be displayed (Appendix, Figure 4)
         - **IEC addition**: Add IEC reservoir plate to Bravo to match *(Figure 1).*

Bravo will ask user to enter number of qPCR plates to be filled (up to a maximum of 15 plates)

- - - 1. When labware is loaded click the “Continue” button on the form. (Appendix Figure 4)
      2. Run configuration wizard will appear. Enter number of **qPCR plates to be filled**

and click “Finish”. (Appendix Figure 5)

- - - 1. Loading Confirmation Box will appear. Follow steps and click “Continue”. (Appendix Figure 6)
      2. Scan in the barcodes as prompted. (Appendix Figure 7)
      3. Check each barcode with your lab partner before continuing: Click “OK”.
      4. If plate barcodes have been scanned more than once an error message will appear. Abort run and re-start protocol.
      5. Enter the lead user ID: Click “OK” protocol will begin. (Appendix figure 8)
      6. Enter the checking user ID: Click “OK”.
      7. The system will now run.
         - Bravo will pause and prompt user to add next qPCR plate.
      8. Proceed to **sample addition.**

If not used immediately, the prepared qPCR plates (containing IEC) can be stored at **+4°C in the dark up to 12 hours.** Mark plate as IEC added, centrifuge qPCR plates at 1200rpm for 1min and seal with appropriate plastic seal e.g. Perkin Elmer Clear Adhesive Film prior to storage at +4°C.


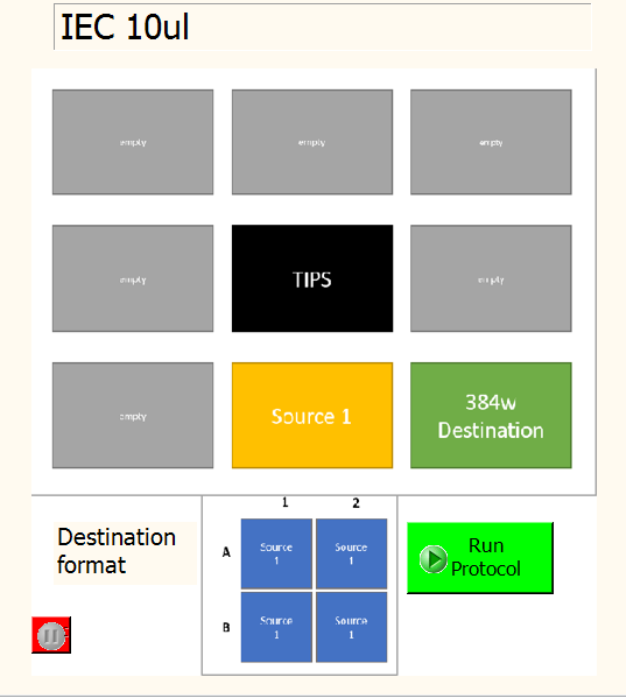


*Figure 1. Agilent Bravo 96LT deck layout for sample addition (Protocol: IEC_10ul_reservoir)*

**A WITNESS IS REQUIRED TO CHECK THE BARCODE SCANNING AND FINAL LAYOUT BEFORE THE RUN**

**CHECK ALL PLATES AND TIPS ARE ORIENTATED WITH A1 POSITIONED TO THE TOP LEFT OF THE SILVER PLATE PADS**

**CHECK TIP BOXES ARE DELIDDED**

**ENSURE TIPS IN POSTIONS A1 AND B1 ARE REMOVED CHECK SEALS HAVE BEEN REMOVED FROM ALL PLATES**

## Note: The protocol is designed to aspirate excess liquid, therefore approximately 5µl liquid will remain in the tips on completion of the run.

## Pre-automation procedure: Sample addition

- - - 1. Collect up to 4 sample source plates (96-well format) in the Station#3b Sample Input Fridge. Perform a quick spin at 1200rpm (in Heraues Multifuge3 S-R with rotor 75006445 or equivalent) and at 4°C to pull liquid to the bottom of the wells. Scan plates into PCR tracking sheet.
      2. Collect 1 x RT-qPCR plate (384-well format) containing Mastermix, NTC and IEC, confirm NTC and IEC addition and plate has been scanned into PCR tracking sheet. (PCR tracking sheet can be found on the CBCTC – Internal, PCR Prep conversation area on Microsoft Teams).
      3. **Remove tips in position A1 and B1**, corresponding to positive and negative control wells, for 4 boxes (or less depending on the number of source plates to process) of Agilent 96 Sterile Filtered tips for use on the Bravo and dispose them in a sealable Bio-bin. Removal of these tips is always witnessed and double checked by a second scientist. Ensure that the remainder of the box contains tips and that they are seated correctly in the box.

## Automation procedure: Sample addition

The plate-to-plate transfer of the heat inactivated samples in 96-well plates into the qPCR 384-well PCR will be done using the Agilent Bravo fitted with a 96LT head.

## Before use, clean the Bravo deck and working area around the robot with suitable alcohol wipes or 70% Ethanol and close the VWorks software if open

- Following addition of IEC, add **samples** to the PCR plate.

**REMOVE TIPS FROM A1 & B1**

- - 10µl assay: Desktop\Covid Method\D2PCR\DirectToPCR_10ul
    - Protocol will add 4µl samples to all 4 quadrants.
    - See Figure 2 for Bravo set up

## Bravo procedure

- - - 1. Open Bravo VWorks software and login. (Appendix Figure 2)
         - Username = Speak to Lab Leader
         - Password = Speak to Lab Leader
      2. Open the correct protocol from the Desktop Folder
         - **DirectToPCR_10** *(Figure 2)*
      3. If prompted to initialize the device say **“Yes”.** (Appendix Figure 3)
      4. The “MANUAL LOADING INSTRUCTIONS” form should be displayed (Appendix, Figure 4) Load the robot to match Figure 2: Sample addition.
         - If 4x source plates are not required load a “DUMMY PLATE” onto empty positions. Treat dummy plates as normal source plates for rest of the method. *(Figure 2)*
      5. When labware is loaded click the “Continue” button on the form. (Appendix Figure 4)
      6. Run configuration wizard will appear. Leave as defaults and click “Finish”. (Appendix Figure 5)
      7. Loading Confirmation Box will appear. Follow steps and click “Continue”. (Appendix Figure 6)
      8. Scan in the barcodes as prompted. (Appendix Figure 7)
      9. Check each barcode with your lab partner before continuing: Click “OK”.
      10. If plate barcodes have been scanned more than once an error message will appear. Abort run and re-start protocol.
      11. Enter the lead user ID: Click “OK”. (Appendix figure 8)
      12. Enter the checking user ID: Click “OK”.
      13. The system will now run.


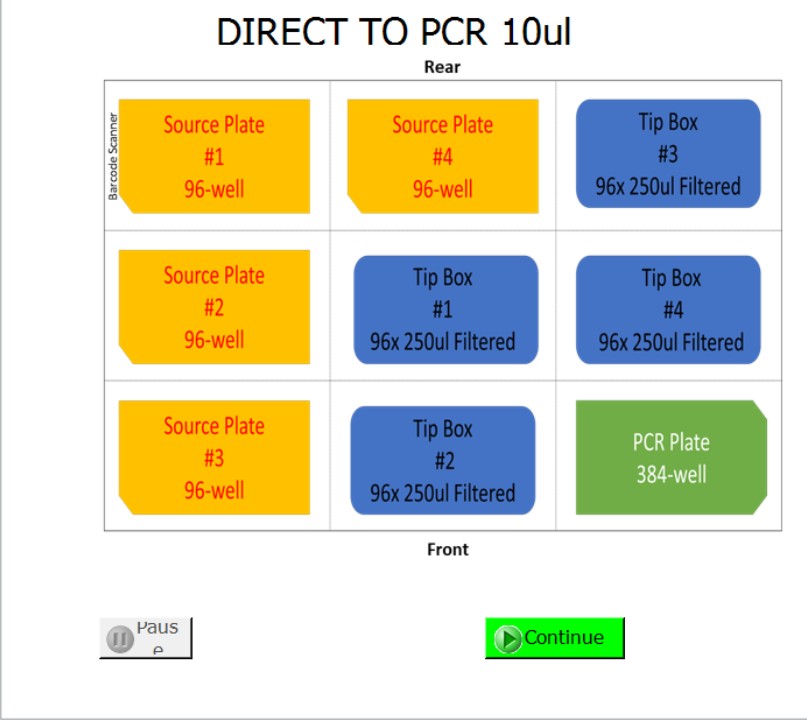


*Figure 2. Agilent Bravo 96LT deck layout for sample addition (Protocol: DirectToPCR_10)*

**A WITNESS IS REQUIRED TO CHECK THE BARCODE SCANNING AND FINAL LAYOUT BEFORE THE RUN**

**CHECK ALL PLATES AND TIPS ARE ORIENTATED WITH A1 POSITIONED TO THE TOP LEFT OF THE SILVER PLATE PADS**

**CHECK TIP BOXES ARE DELIDDED**

**ENSURE TIPS IN POSTIONS A1 AND B1 ARE REMOVED CHECK SEALS HAVE BEEN REMOVED FROM ALL PLATES**

## Note: The protocol is designed to aspirate excess liquid, therefore approximately 2µl liquid will remain in the tips on completion of the run.

- - 1. **Post Automation Procedure**
       1. Loosely apply optical grade LightCycler seal on the 384-well RT-qPCR plate and transfer it to station 3a BSC in the RT-PCR analysis room for addition of the positive control template (see SOP CB 06).
       2. Seal used source plates, clearly mark as used and the date and store them in the RNA output fridge in the annexe of PCR prep room (station 3b).
       3. Remove tip boxes from the Bravo deck and re-lid before disposal.
       4. Clean the Bravo deck and working area with 70% ethanol on lint free tissue or Azowipes. Wipe Dry.

## Data Tracking

Output files for the transfer are saved on the Bravo laptop as a .txt file in the following location: C:\Users\admin\Desktop\OutputFiles\ using the destination PCR plate barcode as file name.

**After the Bravo run:**

1. In LIMfinity, open the create a new PCR plate option. Into this, load the Bravo output file (.txt file, see above) scan the Bravo head used for the transfer, enter the MultiDrop head used for the MasterMix dispense as well as the Master Mix and PCT batch numbers as described in LIMS User Guide. All this information is copied from the saved excel files generated in SP CB 05 Station 3 Preparation of 384-well PCR plate and addition of negative control.

## Move the Bravo transfer output file and the plate reagent file to the Archive Folder

## Waste Management

- Dispose all tips and used tubes in the 2L Bio-bin in the BSC
- Seal the Bio-bins and place in the autoclave bags outside the BSC

1. **RESPONSIBILITIES**

- It is the responsibility of all personnel handling samples to follow this procedure.
- It is the responsibility of personnel performing this procedure to ensure all equipment, chemicals, reagents, and solutions are properly labelled and that proper documentation is maintained.
- It is the responsibility of the laboratory manager to ensure that this procedure is followed and that it is updated, as necessary.

End of SOP CB 34 (Version2)
